# Coordination Behavior of [Cp″_2_Zr(µ^1:1^-As_4_)] towards Lewis Acids

**DOI:** 10.3390/molecules26102966

**Published:** 2021-05-17

**Authors:** Veronika Heinl, Gábor Balázs, Sarah Koschabek, Maria Eckhardt, Martin Piesch, Michael Seidl, Manfred Scheer

**Affiliations:** Faculty for Chemistry and Pharmacy, Institute of Inorganic Chemistry, University of Regensburg, Universitätsstraße 31, 93053 Regensburg, Germany; veronika.heinl@ur.de (V.H.); gabor.balazs@ur.de (G.B.); sarah.koschabek@ur.de (S.K.); maria.parzefall83@gmail.com (M.E.); martin.piesch@ur.de (M.P.); michael1.seidl@ur.de (M.S.)

**Keywords:** arsenic, coordination chemistry, DFT calculations, Lewis acids, zirconium

## Abstract

The functionalization of the arsenic transfer reagent [Cp″_2_Zr(η^1:1^-As_4_)] (**1**) focuses on modifying its properties and enabling a broader scope of reactivity. The coordination behavior of **1** towards different Lewis-acidic transition metal complexes and main group compounds is investigated by experimental and computational studies. Depending on the steric requirements of the Lewis acids and the reaction temperature, a variety of new complexes with different coordination modes and coordination numbers could be synthesized. Depending on the Lewis acid (LA) used, a mono-substitution in [Cp″_2_Zr(µ,η^1:1:1:1^-As_4_)(LA)] (LA = Fe(CO)_4_ (**4**); B(C_6_F_5_)_3_ (**7**)) and [Cp″_2_Zr(µ,η^3:1:1^-As_4_)(Fe(CO)_3_)] (**5**) or a di-substitution [Cp″_2_Zr(µ_3_,η^1:1:1:1^-As_4_)(LA)_2_] (LA = W(CO)_5_ (**2**); CpMn(CO)_2_ (**3**); AlR_3_ (**6**, R = Me, Et, *^i^*Bu)) are monitored. In contrast to other coordination products, **5** shows an η^3^ coordination in which the butterfly As_4_ ligand is rearranged to a *cyclo*-As_4_ ligand. The reported complexes are rationalized in terms of inverse coordination.

## 1. Introduction

**Scheme 1:**
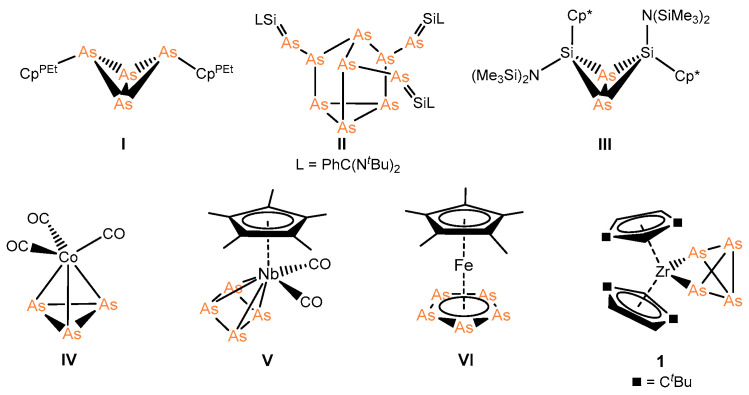
Selected examples of As_n_-containing compounds.

The interest in the activation of small molecules, such as H_2_, N_2_, NH_3_, etc., has increased notably in recent years [1,2,3,4,5,6,7,8]. A special focus lies on the activation of cage compounds such as white phosphorus and yellow arsenic by transition metals and main group compounds [9,10,11,12]. While the synthesis and isolation of polyphosphorus complexes have been intensively investigated [9,10,11], polyarsenic complexes are much less known, and hence the number of comparable polyarsenic complexes is quite limited [12]. This can be attributed to the very challenging handling of As_4_ due to its pronounced air- and light-sensitivity, time-consuming preparation, and to the lack of knowledge regarding its toxicitiy. Nevertheless, by now, several examples of As_n_-containing main group compounds and transition metal complexes are known [12]. Selected representatives are depicted in Scheme 1. A remarkable example is the reaction of yellow arsenic towards the Cp^PEt^ radical (Cp^PEt^ = C_5_(4-EtC_6_H_4_)_5_), which leads to the formation of the first organo-substituted As_4_ butterfly compound [Cp^Pet^_2_As_4_] (**I**, Scheme 1) [13]. Furthermore, the reaction of the silylene [PhC(N*^t^*Bu)_2_SiN(SiMe_3_)_2_] with yellow arsenic results in the aggregation of the As_4_ tetrahedron and compound **II** that contains a heptaarsa-nortricyclane unit can be isolated [14]. Surprisingly, by using the disilene [Cp*(Me_3_Si)_2_NSi = SiN(Me_3_Si)_2_Cp*] (Cp* = C_5_Me_5_), the butterfly-like compound [Cp*{(SiMe_3_)_2_N}SiAs]_2_ (**III**, Scheme 1) is formed [14].

Compared to the As-containing main group compounds, As_n_ ligand complexes of transition metals have been investigated in more detail and show a more extensive chemistry. Dahl et. al. described the first As_n_ ligand complex [(CO)_3_Co(η^3^-As_3_)] (**IV**, Scheme 1) in the late 1960s obtained by the reaction of [Co_2_(CO)_8_] with [AsCH_3_]_5_ [15]. Many of the described syntheses use in situ prepared yellow arsenic and transition metal compounds with labile ligands [12]. For instance, the photolysis of [Cp*Nb(CO)_4_] in the presence of yellow arsenic leads to the formation of **V**, containing a tetraarsacyclobutadiene ligand [16]. Another remarkable example is the pentaarsaferrocene [Cp*Fe(η^5^-As_5_)] (**VI**, Scheme 1) [17]. After its discovery by Scherer et al. in 1990, a versatile chemistry emerged [18,19,20,21,22,23], including, inter alia, redox- [19,20] and coordination chemistry [21,22,23]. Recently, our group reported the synthesis of [Cp″_2_Zr(η^1:1^-As_4_)] (**1**, Scheme 1) by the thermolysis of As_4_ with [Cp″_2_Zr(CO)_2_] (Cp″ = 1,3-di-tertbutyl-cyclopentadienyl) [24]. Further investigations illustrate the high potential of **1** as an arsenic transfer reagent [24,25,26]. Additionally, the phosphorus congener of **1**, i.e., [Cp″_2_Zr(η^1:1^-P_4_)], shows diverse reactivity patterns towards Lewis acids [27]. These results led us to investigate the reaction behavior of **1** towards the same Lewis acids to possibly functionalize the As_4_ core in **1**. That way, compounds with a higher molecular mass should be accessible, resulting, in the case of a transfer of the functionalized As_4_ ligand, in a better solubility of the products.

Herein, we report the coordination behavior of **1** towards different Lewis-acidic transition metal complexes and main group compounds, leading among others to hetero bi- and trimetallic complexes. The structure of these complexes can be rationalized also by means of the concept of inverse coordination [28,29,30,31,32,33]. Within this concept, the structure of complexes is rationalized in the sense that the ligand represents the central entity to which the metal centers are connected. In this type of complex, the distribution of donor and acceptor sites is opposed to the conventional complexes.

## 2. Results and Discussion

### 2.1. General Consideration

**Figure 1:**
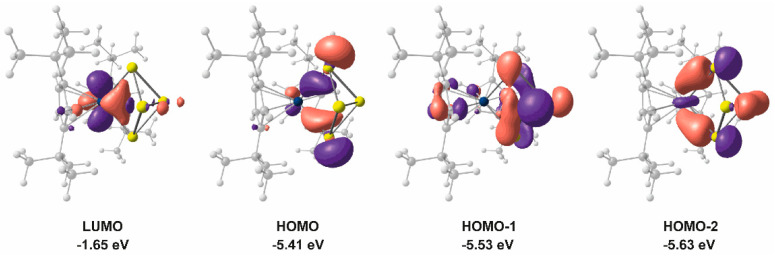
Frontier molecular orbitals (canonical orbitals) of [Cp″_2_Zr(η^1:1^-As_4_)] (**1**) at the B3LYP/def2TZVP level of theory.

**Figure 2:**
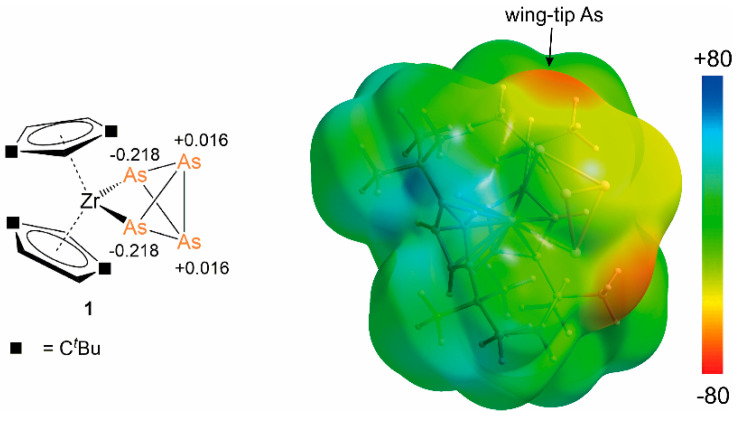
Natural charge distribution (left) of **1** and electrostatic potential mapped on electron density (right). Iso-value 0.001. Color ranges in kJ·mol^−1^.

To obtain a better insight into the electronic structure and to determine the favored coordination site of **1** to Lewis acids, DFT calculations were carried out. The frontier molecular orbitals of **1** at the B3LYP/def2TZVP level of theory are depicted in Figure 1 (see also Supplementary Materials). The highest occupied molecular orbital (HOMO) represents the Zr-As bonding, while the lone pairs of electrons of the bridgehead and wingtip arsenic atoms (arsenic atoms bonded to Zr) are the HOMO-1 and HOMO-2, respectively. Both are energetically close to each other, but the HOMO-2 has a considerably higher arsenic atomic orbital contribution from the wingtip arsenic atoms (64%) compared to the HOMO-1 orbital (26% for the wing tip and 34% from the bridgehead As atoms). This would favor a more effective orbital overlap of the wingtip atoms with a potential acceptor orbital of a Lewis acid. Furthermore, the natural charge distribution shows a negative charge concentration on the wingtip arsenic atoms (nat. charge: −0.218) compared to the bridgehead arsenic atoms (nat. charge: +0.016; Figure 2). The electrostatic potential (Figure 2, right) presents a similar picture, showing that the wingtip As atoms have the highest negative potential. This indicates that a coordination of the electron-rich arsenic atoms directly bound to the zirconium atom should be favored. In contrast, due to the possible steric repulsion of the Cp″ ligand, a coordination of the bridgehead arsenic atoms could still be possible.

Recently, we investigated the coordination behavior of [Cp″_2_Zr(η^1:1^-P_4_)] towards Lewis-acidic metal fragments [27]. The results show that a coordination to Lewis acids via both the wing tip and bridgehead phosphorus atoms is possible, with the coordination via the wing tip P atoms being favored electronically but disfavored sterically. Since the single bond covalent radius of As is larger than that of P (1.21 Å vs. 1.11 Å for As and P, respectively) [34] and inherently the Zr-As bond (2.6619(4) Å to 2.6656(3) Å) [24] is longer compared to the Zr-P bond (2.5596(7) Å to 2.5620(6) Å) [27], one would expect that, for **1**, the electronic effects should predominate and a coordination via wingtip arsenic atoms be preferred.

### 2.2. Coordination of **1** to Transition Metal Complexes

**Scheme 2:**
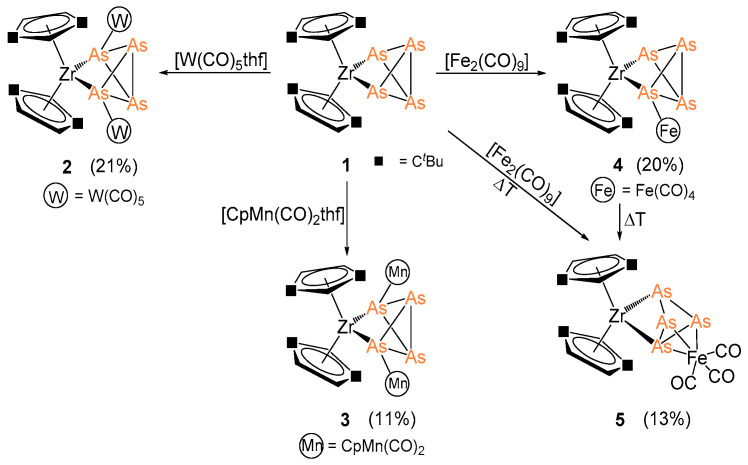
Overview of the reactions of [Cp″_2_Zr(η^1:1^-As_4_)] (**1**) with Lewis-acidic transition metal compounds.

The reaction of **1** with Lewis-acidic transition metal compounds leads to single or double coordination of **1**. Scheme 2 gives an overview of the reactivity of **1** towards transition metal compounds. All products were comprehensively characterized by mass spectrometry, NMR spectroscopy, IR spectroscopy, and single crystal X-ray structure analysis.

The reaction of **1** with [W(CO)_5_thf] and [CpMn(CO)_2_thf] (Cp = cyclopentadienyl) in thf leads to the formation of the hetero trinuclear complexes [Cp″_2_Zr(µ_3_,η^1:1:1:1^-As_4_)(LA)_2_] (LA = W(CO)_5_ (**2**); CpMn(CO)_2_ (**3**)], respectively (Scheme 2). The stoichiometry used has neither an influence on the reaction outcome nor, as a result, on the coordination mode. In contrast, the reaction with [Fe_2_(CO)_9_] in *n*-hexane leads to the formation of the mono-substituted compound [Cp″_2_Zr(µ,η^1:1:1:1^-As_4_)(Fe(CO)_4_)] (**4**), also irrespective of the stoichiometry used. In an attempt to synthesize the di-substituted complex [Cp″_2_Zr(µ,η^1:1:1:1^-As_4_){Fe(CO)_4_}_2_], the reaction of **1** with [Fe_2_(CO)_9_] was performed at different temperatures. Surprisingly, the reaction in refluxing *n*-hexane leads to the formation of [Cp″_2_Zr(µ,η^3:1:1^-As_4_){Fe(CO)_3_}] (**5**). By these reaction conditions, **4** is formed first, followed by CO elimination and the subsequent insertion of the Fe(CO)_3_ fragment into the As-As bond of the bridgehead As atoms (Scheme 2). The same reaction outcome, namely the formation of **5**, can be observed when isolated **4** is heated to 70 °C in *n*-hexane for 2 h. This strongly indicates that **5** is formed from **4**. A similar rearrangement of a P_4_-butterfly core has been reported for the reaction of [(Cp*Cr(CO)_3_}_2_(µ,η^1:1^-P_4_)] with [(Cr(CO)_4_(nbd)] (nbd = norbornadiene) [35]. In **2**, **3** and **4**, the As_4_ ligand in **1** serves as a four-valence electron donor, while, in **5**, it serves as a six-electron donor.

DFT calculations at the B3LYP/def2-SVP level show that the coordination via the wingtip arsenic atoms is favored compared to the bridgehead arsenic atoms (Supplementary Materials). The coordination of one wing tip arsenic atom to one Lewis acid is exothermic with −58, −61 and −105 kJ·mol^−1^ for W(CO)_5_, CpMn(CO)_2_ and Fe(CO)_4_ fragments, respectively, while the coordination of a second fragment is similarly exothermic with −59, −68 and −100 kJ·mol^−1^, respectively (Supplementary Table S5). These are in agreement with the experimental results, where in the reaction solution the formation of both mono and disubstituted species has been observed. In the case of **4** however, only the mono-substituted complex could be isolated, probably due to the lower solubility of **4** compared to the di-substituted derivative as well as the presence of a complex equilibrium in solution (vide infra).

**Figure 3:**
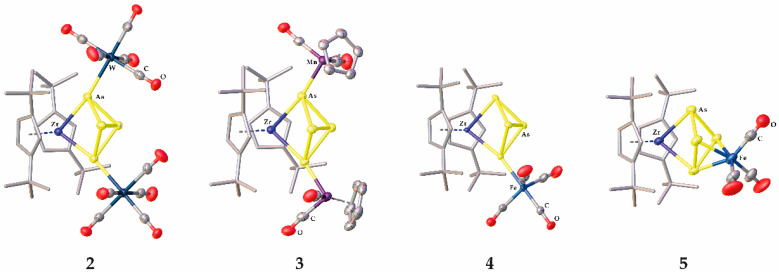
Molecular structures of **2**, **3**, **4** and **5** in the solid state with thermal ellipsoids at 50% probability level. Hydrogen atoms are omitted and the Cp″ ligands are drawn in the tube model for clarity.

Single crystals suitable for X-ray diffractions were obtained by storing a concentrated *n*-hexane (**2**) or *n*-pentane (**3**, **4**, **5**) solution at −78 °C. It should be mentioned that **5** always co-crystallizes with a certain amount of **4** in a ratio of approximately 10:1. The molecular structures in the solid state are depicted in Figure 3. In the compounds **2**, **3** and **4**, one or two arsenic atoms of the As_4_-butterfly moiety of **1** coordinate to the Lewis-acidic metal fragments. Due to the steric repulsion, the As_4_ unit is slightly distorted. The As-As distances are still intact and in the range of a single bond (**2**: 2.4363(6) Å to 2.4649(6)Å; **3**: 2.4394(4) Å to 2.4710(4) Å; **4**: 2.4321(6) Å to 2.4791(4) Å) [13,16,19,24]. Furthermore, the Zr-As distances are slightly elongated compared to **1** [24]. In the case of **2** and **4**, the As-W distances of 2.6560(4) Å and 2.6595(4) Å and the As-Fe distance of 2.4213(7) Å are slightly elongated compared to the sum of the single bond covalent radii (As-W: 2.58 Å; As-Fe: 2.37 Å) [34]. This indicates an elongated single bond between the coordinating As atoms and the metal center of the Lewis acids. In contrast, in **3**, the As-Mn distances (2.3605(4) Å and 2.3732(5) Å) are slightly below the calculated value of 2.40 Å, which implies a single bond [34]. In **5**, the As-As distance between the former bridgehead arsenic atoms is 3.032(3) Å, which clearly shows the cleavage of this bond. The other As-As bonds are still in the range of a single bond (2.4657(14) Å and 2.4721(13) Å) [13,16,19], and the As-Fe distances of 2.4151(16) Å to 2.4265(15) Å are in the same range as observed for **4**.

**Figure 4:**
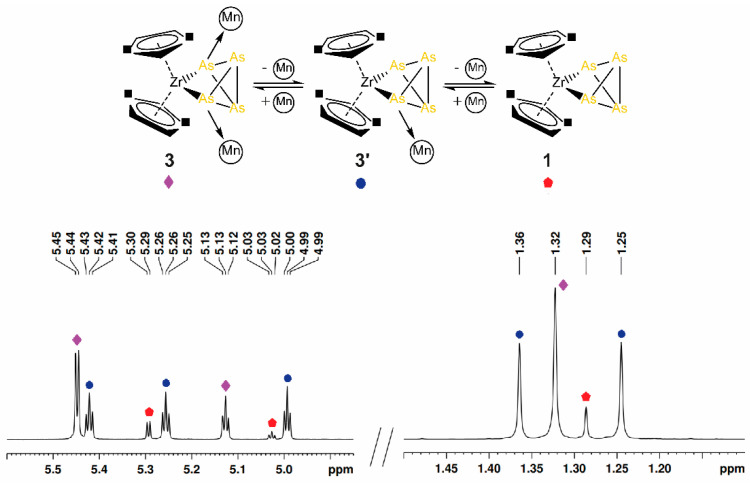
Sections of the ^1^H NMR spectrum of **3** at 293 K in C_6_D_6_ with assignment to the compounds present in solution.

The complexes **2**–**5** were characterized by IR and ^1^H NMR spectroscopy as well as by mass spectrometry. While the expected carbonyl stretches were observed in the IR spectra of **2**, **3**, **4** and **5**, marked differences are present in their ^1^H NMR spectra. The ^1^H NMR spectrum of **2** shows one set of signals for the Cp″ ligands (one singlet for the *^t^*Bu groups at 1.26 ppm and a triplet and a doublet at 5.31 ppm and 5.48 ppm, respectively, for the Cp ring-bound hydrogen atoms), while, in LIFDI-MS, a peak corresponding to [M^+^ − W(CO)_5_] (corresponds to the mono-substituted complex) can be observed (Supplementary Materials). In the ^1^H NMR spectrum of crystalline **3** dissolved in C_6_D_6_, three sets of signals corresponding to Cp″ ligands can be detected. In two of them, the adjacent CH groups are equivalent, indicating that the Cp″ ligands are in a symmetric environment, while, in the third set, they are not equivalent, pointing to an asymmetric compound (Figure 4). One set of signals corresponding to a symmetric Cp″ ligand can be assigned to the starting material **1**, the second set of signals for the symmetric Cp″ ligand is assigned to **3**, based on the comparison with the ^1^H NMR data of **2** which also shows a symmetric Cp″ ligand. The set of signals for the Cp″ ligands in an asymmetric environment can be attributed to [Cp″_2_Zr(µ,η^1:1:1^-As_4_){CpMn(CO)_2_}] (**3′**), based on the comparison with the ^1^H NMR data for **4** (Figure 4; vide infra). These data show that, in solution, **3** partly dissociates to **3′** and to **1** by elimination of one as well as both CpMn(CO)_2_ fragments. Attempts to freeze this dynamic process by lowering the temperature leads only to a temperature-dependent shift of the resonance signals as well as to a change of their relative intensity. By lowering the temperature, the intensity of the resonance signals corresponding to **3** increases and **3** crystallizes from the solution. These results are in stark contrast to the results reported for the related reaction of [Cp″_2_Zr(µ,η^1:1^-P_4_)] with [CpMn(CO)_2_thf], where the phosphorus analog of the mono-substituted **3′** and the coordination of one or both bridgehead phosphorus atoms to the manganese fragment were reported (see Figure S10 in Supplementary Materials) [27].

The ^1^H NMR spectrum of isolated **4**, dissolved in C_6_D_6_, shows one set of signals corresponding to Cp″ ligands in an asymmetric environment, two singlets at 1.15 ppm and 1.28 ppm for the *^t^*Bu groups and three triplets at 5.11 ppm, 5.22 ppm and 5.44 ppm for the CH groups. Therefore, no dynamic behavior in solution similar to that observed for **3** can be detected. In the reaction solution, however, among signals of **4**, also signals corresponding to the di-substituted complex [Cp″_2_Zr(µ_3_,η^1:1:1:1^-As_4_){Fe(CO)_4_}_2_] (**4′**) and **1** can be detected by ^1^H NMR spectroscopy (Supplementary Materials), but only **4** crystallizes from the solution. In the remaining solution, after crystallization of **4**, three sets of signals can still be detected, even though the intensity of the signals of **4** is drastically decreased. Surprisingly, the ^1^H NMR spectrum of **5** shows three signals (*δ* [ppm] = 1.32 (s), 5.84 (d), 5.95 (t)) for the Cp″ substituents indicating a symmetric environment on the NMR timescale (see Figure S14 in Supplementary Materials).

### 2.3. Coordination towards Main Group Compounds

**Scheme 3:**
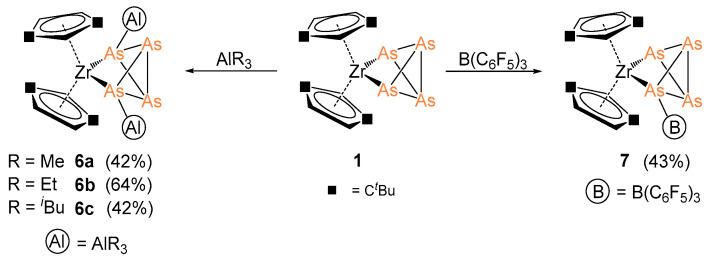
Overview of the reactions of [Cp″_2_Zr(η^1:1^-As_4_)] (**1**) with Lewis-acidic main group compounds.

In order to investigate whether the nature of the Lewis acid plays a crucial role in the coordination behavior of **1**, the reactivity of **1** towards Lewis-acidic main group compounds was investigated (Scheme 3). Thus, the reaction with AlR_3_ (R = Me, Et, *^i^*Bu) in toluene was performed, leading to [Cp″_2_Zr(µ_3_,η^1:1:1:1^-As_4_)(AlR_3_)_2_] (**6a**: R = Me; **6b**: R = Et; **6c**: R = *^i^*Bu), showing twofold coordination of **1** to the AlR_3_ units. The reaction outcome is independent of the stoichiometry used, although the yield can be increased by using an excess of the Lewis acid. In contrast, the reaction with [B(C_6_F_5_)_3_] leads to the formation of [Cp″_2_Zr(µ_3_,η^1:1:1:1^-As_4_){B(C_6_F_5_)_3_}] (**7**). Probably due to the pronounced steric requirement of [B(C_6_F_5_)_3_], only the formation of the mono-substituted compound **7** seems to be possible, interestingly also at a wingtip arsenic atom. The less Lewis acidic Et_3_B do not reacts with **1**.

DFT calculations support the reaction outcome (B3LYP/def2-SVP level of theory; for further information, see Supplementary Materials). In the case of the compounds **6** and **7**, the coordination of the arsenic atoms directly bound to the zirconium is favored (−24 kJ·mol^−1^ (**6a**), −33 kJ·mol^−1^ (**6b**), −7 kJ·mol^−1^ (**6c**)) (see Supplementary Materials) as compared to the coordination of the bridgehead arsenic atoms. Furthermore, the formation of the di-substituted compounds **6a**, **6b**, and **6c** is energetically clearly favored (**6a**: −28 kJ·mol^−1^; **6b**: −24 kJ·mol^−1^; **6c**: −7 kJ·mol^−1^) over the mono-substituted compounds. These results unambiguously underline the experimental results.

**Figure 5:**
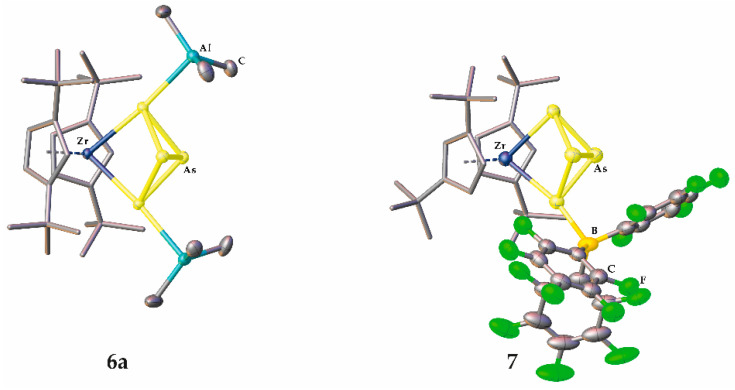
Molecular structures of **6a** and **7** in the solid state with thermal ellipsoids at 50% probability level. Hydrogen atoms are omitted, the Cp″ ligands and the [B(C_6_F_5_)_3_] ligand are drawn in the tube model for clarity.

Single crystals suitable for X-ray diffractions can be obtained by storing a concentrated *n*-pentane (**6a**, **6b**, **6c**) or *n*-hexane (**7**) solution at −78 °C. The molecular structures of **6a** and **7** are exemplified in Figure 5. In the case of the compounds **6**, a di-coordination of **1** is observed, while, in **7**, only the mono-coordination of **1** to boron occurs. The geometric parameters of **6a**, **6b,** and **6c** are very similar, therefore, only **6a** will be discussed further (for **6b**, **6c** see Supplementary Materials) herein. In **6a** and **7**, the As_4_-butterfly unit is only slightly distorted, which can be attributed to the steric repulsions. The As-As distances in **6a** of 2.4272(4) Å to 2.4583(3) Å and **7** of 2.436(3) Å to 2.457(4) Å do not differ considerably from that reported for **1** [24]. However, the Zr-As distances are slightly elongated (**6a**: 2.6810(2) Å; **7**: 2.732(3) Å) [24]. As in **2**–**4**, the As-Al (**6a**: 2.6743(5) Å) and As-B (**7**: 2.177(6) Å) distances in **6a** and **7** are slightly longer than the sum of the covalent radii (As-Al: 2.47 Å and As-B: 2.06 Å) [34].

The ^1^H NMR spectra of **6a**, **6b**, **6c,** and **7** show the expected signals for the Cp″ ligands as well as the signals of the alkyl groups bonded to aluminum in compound **6**. A doublet and a triplet corresponding to the hydrogen atoms directly bonded to the cyclopentadienyl ligands resonate between δ = 5.0 ppm to 5.78 ppm for **6**. The corresponding signals for **7** are slightly more deshielded and resonate at 5.88 ppm and 6.06 ppm. Furthermore, a sharp singlet for the *^t^*Bu groups is observed (δ = 1.27 ppm (**6a**); 1.26 ppm (**6b**, **7**); 1.29 ppm (**6c**)). Additionally, the expected signals for the alkyl groups of the aluminum Lewis acids appear, for **6a** a singlet at −0.29 ppm (Supplementary Materials for **6b** and **6c**).

## 3. Materials and Methods

### 3.1. General Informations

All experiments were performed under an atmosphere of dry nitrogen or argon using Schlenk and glovebox techniques. Solvents were purified, dried and degassed prior to use. ^1^H, ^13^C{^1^H} NMR spectra were recorded at room temperature on a Bruker Avance 400 spectrometer (^1^H: 400,13 MHz, ^13^C: 100.61 MHz). ^1^H, ^13^C NMR chemical shifts are reported in parts per million (ppm) relative to the external standard Me_4_Si. The elemental analysis was determined with a Vario micro cube apparatus. For mass spectrometry, a Finnigan MAT 95 (LIFDI MS, FD MS) or a Finnigan MAT SSQ 710 A (EI MS) device and a Joel AccuTOF GCX spectrometer were used. [Fe_2_(CO)_9_] [36,37] and [Cp″_2_Zr(η^1:1^-As_4_)] [24] were prepared according to literature procedures.

### 3.2. Synthesis and Characterization of the Compounds **2**–**7**

#### 3.2.1. Synthesis and Characterization of [Cp″_2_Zr(µ_3_,η^1:1:1:1^-As_4_)(W(CO)_5_)_2_] (**2**)

W(CO)_6_ (43 mg, 0.13 mmol) is dissolved in 50 mL thf and irradiated with UV light for 1 h using a low-pressure mercury lamp (TQ 150). The pale-yellow solution is added to a solution of [Cp″_2_Zr(η^1:1^-As_4_)] (**1**) (50 mg, 0.067 mmol) and stirred for 24 h. After removing the solvent in vacuo, the brown residue is extracted with *n*-hexane. Red crystals of **2** suitable for single crystal X-ray structure analysis were obtained by storing a concentrated solution at −78 °C. Crystalline yield: 19 mg (0.014 mmol, 21%).

^1^H NMR (C_6_D_6_, 298 K): *δ* [ppm] = 1.26 (s, 36 H, C*CH_3_*), 5.31 (t, br, 2 H, C_5_*H_3_^t^*Bu_2_), 5.48 (d, 4 H, C_5_*H_3_^t^*Bu_2_); IR (toluene): ν [cm^−1^] = 2064 (m), 1977 (vs), 1937 (s); elemental analysis (%): calculated for [C_36_H_42_ZrAs_4_W_2_O_10_] (1391.77 g/mol): C, 31.03; H, 3.04; O, 11.48. No satisfying elemental analysis could be obtained, not even by using Sn capsules. This is caused by the air sensitivity of compound **2**; FD MS (toluene): *m*/*z* (%): 1393.8 (12) [M]^+^, 1070.2 (M^+^-[W(CO)_5_], 100); crystal data for C_36_H_42_As_4_O_10_W_2_Zr, triclinic, *P*-1, a = 11.0989(3) Å, b = 12.5065(3) Å, c = 17.5582(5) Å, α = 101.764(2)°, β = 95.394(2)°, γ = 113.207(2)°, V = 2151.41(10) Å^3^.

#### 3.2.2. Synthesis and Characterization of [Cp″_2_Zr(µ_3_,η^1:1:1:1^-As_4_)(CpMn(CO)_2_)_2_] (**3**)

A solution of [CpMn(CO)_3_] (56 mg, 0.27 mmol) is dissolved in 50 mL thf and irradiated with UV light for 1 h using a low-pressure mercury lamp (TQ 150). The pale-rose solution is added to solid [Cp″_2_Zr(η^1:1^-As_4_)] (**1**) (100 mg, 0.13 mmol). After stirring for 16 h at room temperature, the solvent of the red-brown reaction mixture is removed in vacuo, extracted with 10 mL *n*-pentane and filtered via cannula. A red solution is obtained. Violet crystals of **3** suitable for single crystal X-ray structure analysis were obtained by storing a concentrated solution at −78 °C. Crystalline yield: 15 mg (0.014 mmol, 11%).

^1^H NMR (C_6_D_6_, 298 K): *δ* [ppm] = 1.25 (s, 18 H, C*CH_3_*), 1.36 (s, 36 H, C*CH_3_*), 4.99 (t, 2 H, C_5_*H_3_^t^*Bu_2_), 5.26 (t, 2 H, C_5_*H_3_^t^*Bu_2_), 5.42 (t, 2 H, C_5_*H_3_^t^*Bu_2_); elemental analysis (%): calculated for [C_40_H_52_ZrAs_4_Mn_2_O_4_] (1097.62 g·mol^−1^): C, 43.77; H, 4.78; found: C, 42.68; H, 5.13; IR (toluene): ν[cm^−1^] = 1928 (s), 1873 (s); LIFDI MS (toluene): *m*/*z* (%): 1095.9 (M^+^, 42), 919.9 (M^+^-[CpMn(CO)_2_], 100); crystal data for C_40_H_52_As_4_Mn_2_O_4_Zr, triclinic, *P*$\bar{1}$ *P*-1, a = 11.4684(3) Å, b = 12.1340(4) Å, c = 16.8248(4) Å, α = 80.103(2)°, β = 89.979(2)°, γ = 63.838(2)°, V = 2080.60(11) Å^3^.

#### 3.2.3. Synthesis and Characterization of [Cp″_2_Zr(µ,η^1:1:1^-As_4_)(Fe(CO)_4_)] (**4**)

A solution of [Cp″_2_Zr(η^1:1^-As_4_)] (**1**) (60 mg, 0.081 mmol) in 10 mL *n*-hexane is added to a suspension of [Fe_2_(CO)_9_] (58 mg, 0.16 mmol) in 10 mL *n*-hexane. The reaction mixture is stirred at room temperature for 24 h. From the brown reaction mixture, the solvent was removed in vacuo. The brown residue was dissolved in 5 mL *n*-pentane and filtered via cannula. Orange crystals of **4** suitable for single crystal X-ray structure analysis were obtained by storing a concentrated solution at −78 °C. Crystalline yield: 11 mg (0.016 mmol, 20%).

^1^H NMR (C_6_D_6_, 298 K): *δ* [ppm] = 1.15 (s, 18 H, C*CH_3_*), 1.28 (s, 18 H, C*CH_3_*), 5.11 (t, 2 H, C_5_*H_3_^t^*Bu_2_), 5.22 (t, 2 H, C_5_*H_3_^t^*Bu_2_), 5.44 (t, 2 H, C_5_*H_3_^t^*Bu_2_); elemental analysis (%): calculated for [C_30_H_42_ZrAs_4_FeO_4_] (911.83 g/mol): C, 39.45; H, 4.63. No satisfying elemental analysis could be obtained, not even by using Sn capsules. This is caused by the air sensitivity of compound **4**; ATR-IR (diamond crystal): ν[cm^−1^] = 2021 (m), 1949 (m), 1918 (s); LIFDI MS (toluene): *m*/*z* (%): 911.8 (M^+^, 100); crystal data for C_30_H_42_As_4_FeO_4_Zr, monoclinic, *P*2_1_/*m*, a = 9.1335(2) Å, b = 17.1283(3) Å, c = 11.3330(2) Å, β = 104.114(2)°, α = γ = 90°, V = 1719.43(6) Å^3^.

#### 3.2.4. Synthesis and Characterization of [Cp″_2_Zr(µ,η^3:1:1^-As_4_)(Fe(CO)_3_)] (**5**)

A solution of [Cp″_2_Zr(η^1:1^-As_4_)] (**1**) (60 mg, 0.081 mmol) in 10 mL *n*-hexane is added to a suspension of [Fe_2_(CO)_9_] (58 mg, 0.24 mmol) in 10 mL *n*-hexane. The reaction mixture is refluxed for 3 h. All volatiles of the brown mixture are removed in vacuo, extracted with 10 mL of *n*-pentane and filtered via cannula. Violet crystals of **5** suitable for single crystal X-ray structure analysis were obtained by storing a concentrated solution at −78 °C. Crystalline yield: 9 mg (0.01 mmol, 13%).

^1^H NMR (C_6_D_6_, 298 K): *δ* [ppm] = 1.32 (s, 36 H, C*CH_3_*), 5.84 (d, 4 H, C_5_*H_3_^t^*Bu_2_), 5.95 (t, 2 H, C_5_*H_3_^t^*Bu_2_); elemental analysis (%): calculated for [C_26_H_42_ZrAs_4_(Fe(CO)_4_)]_0.1_[C_26_H_42_ZrAs_4_(Fe(CO)_3_)]_0.9_ + 0.45·[C_5_H_12_] (as found in the X-ray structure, cf. Supplementary Materials): C, 40.90; H, 5.19; found: C, 40.71; H, 4.77; ATR-IR (diamond crystal): ν[cm^−1^] = 1919 (m), 2016 (m); LIFDI MS (toluene): *m*/*z* (%): 883.8 (M^+^, 100); crystal data for C_33.2_H_52.8_As_4_FeO_2.7_Zr, orthorhombic, *Pnma*, a = 12.0528(5) Å, b = 12.2716(7) Å, c = 18.8597(6) Å, α = β = γ = 90°, V = 3926.0(3) Å^3^.

#### 3.2.5. Synthesis and Characterization of [Cp″_2_Zr(µ_3_,η^1:1:1:1^-As_4_){AlR_3_}_2_] (R = Me (**6a**), Et (**6b**), ^i^Bu (**6c**))

A solution of AlR_3_ (**6a**: 0.17 mL, c = 2.0 mol/L, 0.34 mmol; **6b**: 0.34 mL, c = 1.0 mol/L, 0.34 mmol; **6c**: 0.21 mL, c = 1 mol/L, 0.21 mmol) is added via a syringe to a solution of [Cp″_2_Zr(η^1:1^-As_4_)] (**1**) (50 mg, 0.067 mmol) in 10 mL toluene. The reaction mixture is stirred for 3 h at room temperature. After removing the solvent in vacuo, the orange-red residue is dissolved in *n*-pentane and filtered via cannula. Orange crystals of **6a**/**6b**/**6c** suitable for single crystal X-ray structure analysis were obtained by storing a concentrated solution at −78 °C. Crystalline yield: **6a**: 25 mg (0.028 mmol, 42%); **6b**: 42 mg (0.043 mmol, 64%); **6c**: 32 mg (0.028 mmol, 42%).

**6a:** ^1^H NMR (C_6_D_6_, 298 K): *δ* [ppm] = −0.29 (s, 18 H, Al*CH_3_*), 1.27 (s, 36 H, C*CH_3_*), 5.06 (t, 2 H, C_5_*H_3_^t^*Bu_2_), 5.30 (d, 4 H, C_5_*H_3_^t^*Bu_2_); ^13^C{^1^H} NMR (C_6_D_6_, 298 K): *δ* [ppm] = −6.5 (s, Al(*C*H_3_)_3_), 32.6 (s, *C*(CH_3_)_3_), 34.1 (s, *C*H_3_), 101.5 (s, *C*_5_H*_3_^t^*Bu_2_), 107.4 (s, *C*_5_H*_3_^t^*Bu_2_), 137.1 (s, *C*_5_H*_3_^t^*Bu_2_); elemental analysis (%): calculated for [C_32_H_60_ZrAs_4_Al_2_] (888.02 g·mol^−1^): C, 43.20; H, 6.80; found: C, 43.66; H, 6.66; LIFDI MS (toluene): No peaks detected, due to the high volatility of **6a**; Crystal Data for 0.5·(C_32_H_60_Al_2_As_4_Zr), monoclinic, *I*2/*a*, a = 17.6419(3) Å, b = 9.9522(2) Å, c = 23.1811(4) Å, β = 109.190(2)°, α = γ = 90°, V = 3843.88(13) Å^3^.

**6b**: ^1^H NMR (toluene-d_8_, 298 K): *δ* [ppm] = 0.27 (q, 12 H, Al(*CH_2_*CH_3_), 1.26 (s, 36 H, C*CH_3_*), 1.29 (m, 18 H, Al(CH_2_C*H_3_*), 5.00 (t, 2 H, C_5_*H_3_^t^*Bu_2_), 5.27 (d, 4 H, C_5_*H_3_^t^*Bu_2_); ^13^C{^1^H} NMR (C_6_D_6_, 298 K): *δ* [ppm] = 32.5 (s, *C*(CH_3_)_3_), 34.1 (s, *C*H_3_), 101.3 (s, *C*_5_H*_3_^t^*Bu_2_), 107.1 (s, *C*_5_H*_3_^t^*Bu_2_), 136.6 (s, *C*_5_H*_3_^t^*Bu_2_); elemental analysis (%): calculated for [C_38_H_72_ZrAs_4_Al_2_] (972.12 g·mol^−1^): C, 46.87; H, 7.45; found: C, 46.85; H, 7.11; LIFDI MS (toluene): No peaks detected, due to the high volatility of 6b; crystal data for 0.5·(C_38_H_72_Al_2_As_4_Zr), monoclinic, *C*2/*c*, a = 25.1728(4) Å, b = 10.3746(2) Å, c = 18.0895(3) Å, β = 110.797(2)°, α = γ = 90°, V = 4416.40(14) Å^3^.

**6c:** ^1^H NMR (thf-d_8_, 298 K): *δ* [ppm] = −0.08 (d, 12 H, Al(C*H_2_*CH{CH_3_}_2_), 0.91 (d, 36 H, Al(CH_2_CH{C*H_3_*}_2_), 1.29 (s, 36 H, C*CH_3_*), 1.81 (m, 6 H, Al(CH_2_C*H*{CH_3_}_2_), 5.08 (t, 2 H, C_5_*H_3_^t^*Bu_2_), 5.76 (d, 4 H, C_5_*H_3_^t^*Bu_2_); ^13^C{^1^H} NMR (C_6_D_6_, 298 K): *δ* [ppm] = 24.8 (s, Al(*^i^Bu*)_3_), 26.7 (s, Al(*^i^Bu*)_3_), 28.5 (s, Al(*^i^Bu*)_3_), 32.6 (s, *C*(CH_3_)_3_), 34.1 (s, *C*H_3_), 101.2 (s, *C*_5_H*_3_^t^*Bu_2_), 107.0 (s, *C*_5_H*_3_^t^*Bu_2_), 136.3 (s, *C*_5_H*_3_^t^*Bu_2_); elemental analysis (%): calculated for [C_50_H_96_ZrAs_4_Al_2_] (888.02 g·mol^−1^): C, 52.58; H, 8.45; found: C, 52.62; H, 8.15. LIFDI MS (toluene): No peaks detected, due to the high volatility of **6c**; crystal data for 0.5·(C_50_H_96_Al_2_As_4_Zr), monoclinic, *C*2/*c*, a = 18.5668(4) Å, b = 11.9157(2) Å, c = 27.2938(5) Å, β = 110.737(2)°, α = γ = 90°, V = 5647.2(2) Å^3^.

#### 3.2.6. Synthesis and Characterization of [Cp″_2_Zr(µ,η^1:1:1^-As_4_)(B(C_6_F_5_)_3_)] (**7**)

A solution of [Cp″_2_Zr(η^1:1^-As_4_)] (**1**) (60 mg, 0.081 mmol) in 10 mL *n*-hexane is added to a solution of [B(C_6_F_5_)_3_] (81 mg, 0.16 mmol) in 10 mL *n*-hexane at −60 °C. The color changes immediately to brown and a brown solid is formed. After stirring for 2 h at −60 °C and further 2 h at room temperature, the solvent is removed in vacuo. The brown residue is extracted with 5 mL of *n*-hexane and filtered via a cannula. Orange crystals of **7** suitable for single crystal X-ray structure analysis were obtained by storing the solution at −78 °C. Crystalline yield: 44 mg (0.034 mmol, 43%).

^1^H NMR (thf-d_8_, 298 K): *δ* [ppm] = 1.26 (s, 36 H, C*CH_3_*), 5.88 (d, 4 H, C_5_*H_3_^t^*Bu_2_), 6.06 (t, 2 H, C_5_*H_3_^t^*Bu_2_). ^19^F{^1^H} NMR (thf-d_8_, 298 K): *δ* [ppm] = −162.28 (m, B(C_6_*F*_5_)_3_, meta), −155.46 (m, B(C_6_*F*_5_)_3_, para), −130.84 (m, B(C_6_*F*_5_)_3_, ortho). ^19^F NMR (thf-d_8_, 298 K): *δ* [ppm] = −162.28 (m, B(C_6_*F*_5_)_3_, meta), −155.46 (m, B(C_6_*F*_5_)_3_, para), −130.84 (m, B(C_6_*F*_5_)_3_, ortho); elemental analysis (%): calculated for [C_44_H_42_ZrAs_4_BF_15_] (1255.91 g/mol): C, 42.03; H, 3.37. No satisfying elemental analysis could be obtained, not even by using Sn capsules. This is caused by the air sensitivity of compound **7**; LIFDI MS (toluene): Due to the instability of **7**, only fragments could be detected. Inter alia: *m*/*z* (%): 966.75 ([Cp″_2_Zr(B_2_(C_6_F_5_)_3_]), 742.5 (M^+^ − B(C_6_F_5_)_3_); crystal data for C_44_H_42_As_4_BF_15_Zr, triclinic, *P*-1, a = 11.2797(4) Å, b = 12.7421(5) Å, c = 18.5702(6) Å, α = 71.815(3)°, β = 87.410(3)°, γ = 65.217(4)°, V = 2290.82(16) Å^3^.

## 4. Conclusions

In summary, herein, we reported the synthesis and characterization of a variety of coordination compounds based on [Cp″_2_Zr(η^1:1^-As_4_)] (**1**). Computational studies show the preferred coordination of the wingtip arsenic atoms for coordination as well as the preference for the formation of the di-substituted compounds. Experimentally, the coordination of the two wingtip arsenic atoms is observed for the transition metal complexes [Cp″_2_Zr(µ_3_,η^1:1:1:1^-As_4_)(W(CO)_5_)_2_] (**2**) and [Cp″_2_Zr(µ_3_,η^1:1:1:1^-As_4_)(CpMn(CO)_2_)_2_] (**3**). In contrast, the reaction of **1** with [Fe_2_(CO)_9_] leads to the mono-substituted complex [Cp″_2_Zr(µ,η^1:1:1^-As_4_)(Fe(CO)_4_)] (**4**). NMR investigations show the formation of both the mono-substituted (**4**) and the di-substituted [Cp″_2_Zr(µ_3_,η^1:1:1^-As_4_){Fe(CO)_4_}_2_] compounds, however, only **4** can be isolated. Elimination of CO from **4** leads to the formation of [Cp″_2_Zr(µ,η^3:1:1^-As_4_)(Fe(CO)_3_)] (**5**) in which the As_4_-butterfly core is rearranged to a *cyclo*-As_4_ ligand. In solutions, isolated **3** shows an interesting equilibrium between **3**, the mono-substituted complex [Cp″_2_Zr(µ,η^1:1:1:1^-As_4_){CpMn(CO)_2_}] and **1**, while **4** does not dissociate in solution.

The comparison of the reactivity of **1** and the phosphorus congener [Cp″_2_Zr(µ,η^1:1^-P_4_)] towards transition metal-based Lewis acids reveals pronounced differences. While [Cp″_2_Zr(µ,η^1:1^-P_4_)] can coordinate to LAs via both wingtip and bridgehead phosphorus atoms and can form mono-, di- and tri-substituted complexes, **1** coordinates solely through the wingtip arsenic atoms and forms only mono- or di-substituted complexes, with the latter prevailing.

Moreover, **1** reacts with Lewis-acidic main group compounds in a similar way as with transition metal-based LAs. Here, also a twofold coordination is observed for the compounds [Cp″_2_Zr(µ_3_,η^1:1:1:1^-As_4_){AlR_3_}_2_] (R = Me (**6a**), Et (**6b**), *^i^*Bu (**6c**)). In contrast, the mono-substituted compound **7** is formed in the reaction with [B(C_6_F_5_)_3_] due to sterical reasons.

## Data Availability

The data presented in this study are available on request from the corresponding authors.

## Supplementary Materials

The following ones are available online. Crystallographic Data, ^1^H NMR spectra and computational details for the mentioned compounds.

## Sample Availability

Samples of the compounds are available from the authors.
