# Supplementary material for: Coordination Behavior of [Cp″2Zr(µ1:1-As4)] towards Lewis Acids
Source: Molecules. 2021 May 17;26(10):2966. doi: 10.3390/molecules26102966 (PMC8156824; doi:10.3390/molecules26102966)
Supplement: Supplementary file 1 [file molecules-26-02966-s001.zip › molecules-1209150-supplementary publiziert revised.pdf]

Supplementary Information

# Coordination Behavior of [$Cp''_2Zr(\eta^{1:1}-As_4)$] towards Different Lewis Acids

**Veronika Heinl, Gábor Balázs, Sarah Koschabek, Maria Eckhardt, Martin Piesch, Michael Seidl and Manfred Scheer ***

Faculty for Chemistry and Pharmacy, Institute of Inorganic Chemistry, University of Regensburg, Universitätsstraße 31, 93053 Regensburg, Germany; veronika.heinl@ur.de (V.H.); gabor.balazs@ur.de (G.B.); sarah.koschabek@ur.de (S.K.); maria.parzefall83@gmail.com (M.E.); martin.piesch@ur.de (M.P.); michael1.seidl@ur.de (M.S.)

* Correspondence: manfred.scheer@ur.de; Tel.: +49-(0)941-943-4440; Fax: +49-(0)941-943-4439

**Contents:**

1. Crystallographic data

2. NMR investigations

3. Computational details

4. References

## 1. Crystallographic data

Crystals suitable for single crystal X-ray diffraction analysis were obtained as described above. The diffraction intensities were collected either on a Gemini Ultra diffractometer equipped with a Ruby or an Atlas[S2] CCD detector using Cu-$K_\alpha$ radiation (fine-focus sealed X-ray tube) (**2**, **5**), at a XtaLAB Synergy R, DW system equipped with a HyPix-Arc 150 detector using Cu-$K_\alpha$ radiation (rotating-anode) (**7**), a GV50 diffractometer equipped with a Titan[S2] CCD detector using Cu-$K_\alpha$ or Cu-$K_\beta$ radiation (micro-focus sealed X-ray tube) (**3**, **6a**, **6b**, **6c**) or at a SuperNova diffractometer equipped with an Atlas CCD detector using Cu-$K_\alpha$ radiation (micro-focus sealed X-ray tube) (**4**). Data collection and reduction were performed with the **CrysAlisPro** software package (version 1.171.35.15 (**2**), 1.171.40.14a (**3**, **4**, **5**, **6c**); 1.171.40.18c (**6b**); 1.171.41.83a (**7**); 1.171.41.90a (**6a**)).[1] A numerical absorption correction based on gaussian integration over a multifaceted crystal model and an empirical absorption correction using spherical harmonics, implemented in SCALE3 ABSPACK scaling algorithm, was performed for the compounds (**3**, **4**, **6b**, **6c**, **7**).[1] An analytical numeric absorption correction using a multifaceted crystal model based on expressions derived by R. C. Clark & J. S. Reid. (Clark, R. C. & Reid, J. S. (1995). Acta Cryst. A51, 887-897) and an empirical absorption correction using spherical harmonics, implemented in SCALE3 ABSPACK scaling algorithm, was performed for compound (**5**).[1] A multi-scan absorption correction using spherical harmonics, implemented in SCALE3 ABSPACK scaling algorithm, was performed for the compounds (**2**, **6a**).[1] Using **Olex2**,[2] the structures were solved with **olex2.solve**[3] (**2**, **6b**, **7**) or **ShelXT**[4] (**3**, **4**, **5**, **6a**, **6c**) and refined by full-matrix least-squares method against $|F|^2$ using **ShelXL**[4] All non-hydrogen atoms were refined anisotropically. Hydrogen atoms at the carbon atoms were located in idealized positions and refined with isotropic displacement parameters according to the riding model. Using **Olex2**,[2] all pictures of the respective molecular structures were made.

CCDC reference numbers 2077256 (**2**), 2077257 (**3**), 2077258 (**4**), 2077259 (**5**), 2077260 (**6a**), 2077261 (**6b**), 2077262 (**6c**) and 2077263 (**7**) contain the supplementary crystallographic data for this paper. These data can be obtained free of charge at www.ccdc.cam.ac.uk/conts/retrieving.html (or from the Cambridge Crystallographic Data Centre, 12 Union Road, Cambridge CB2 1EZ, UK; Fax: + 44-1223-336-033; e-mail: deposit@ccdc.cam.ac.uk).

**[Cp''$_2$Zr($\eta^{1:1:1:1}$-As$_4$)(W(CO)$_5$)$_2$] (2):**

Compound **2** crystallizes out of a concentrated *n*-hexane solution at -78 °C in form of red blocks in the triclinic space group *P*-1. The asymmetric unit contains one molecule of **2**.

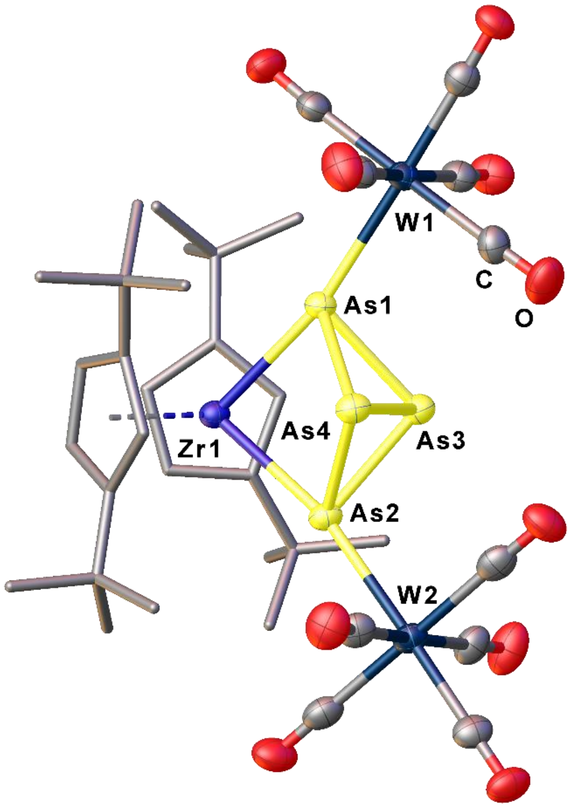


**Figure S1:** Molecular structure of **2** in the solid state. Thermal ellipsoids are depicted at 50% probability level. Hydrogen atoms are omitted and the Cp'' ligands are drawn in the tube model for clarity. Selected bond lengths [Å] and angles [°]: As1-As2 2.4575(6), As1-As3 2.4616(6), As1-W1 2.6460(4), As1-Zr1 2.7021(5), As2-As3 2.4363(6), As2-As4 2.4558(6), As3-As4 2.4649(6), As4-W2 2.6595(4), As4-Zr1 2.7008(5); As2-As1-As3 59.374(18), As2-As1-W1 104.814(19), As2-As1-Zr1 82.962(17), As3-As1-W1 107.84(2), As3-As1-Zr1 83.495(18), W1-As1-Zr1 168.38(2), As3-As2-As1 60.397(18), As3-As2-As4 60.508(18), As4-As2-As1 93.02(2), As1-As3-As4 92.70(2), As2-As3-As1 60.229(18), As2-As3-As4 60.139(18), As2-As4-As3 59.352(18), As2-As4-W2 107.436(19), As2-As4-Zr1 83.020(18), As3-As4-W2 106.51(2), As3-As4-Zr1- 83.461(18), W2-As4-Zr 1168.14(2).

**[Cp''$_2$Zr($\eta^{1:1:1:1}$-$As_4$)(CpMn(CO)$_2$)$_2$] (3):**

Compound **3** crystallizes out of a concentrated *n*-pentane solution at -78 °C in form of violet blocks in the triclinic space group $P\overline{1}$. The asymmetric unit contains one molecule of **3**. One [CpMn(CO)$_2$] fragment is disordered over two positions (occupancy of 0.52 and 0.48).

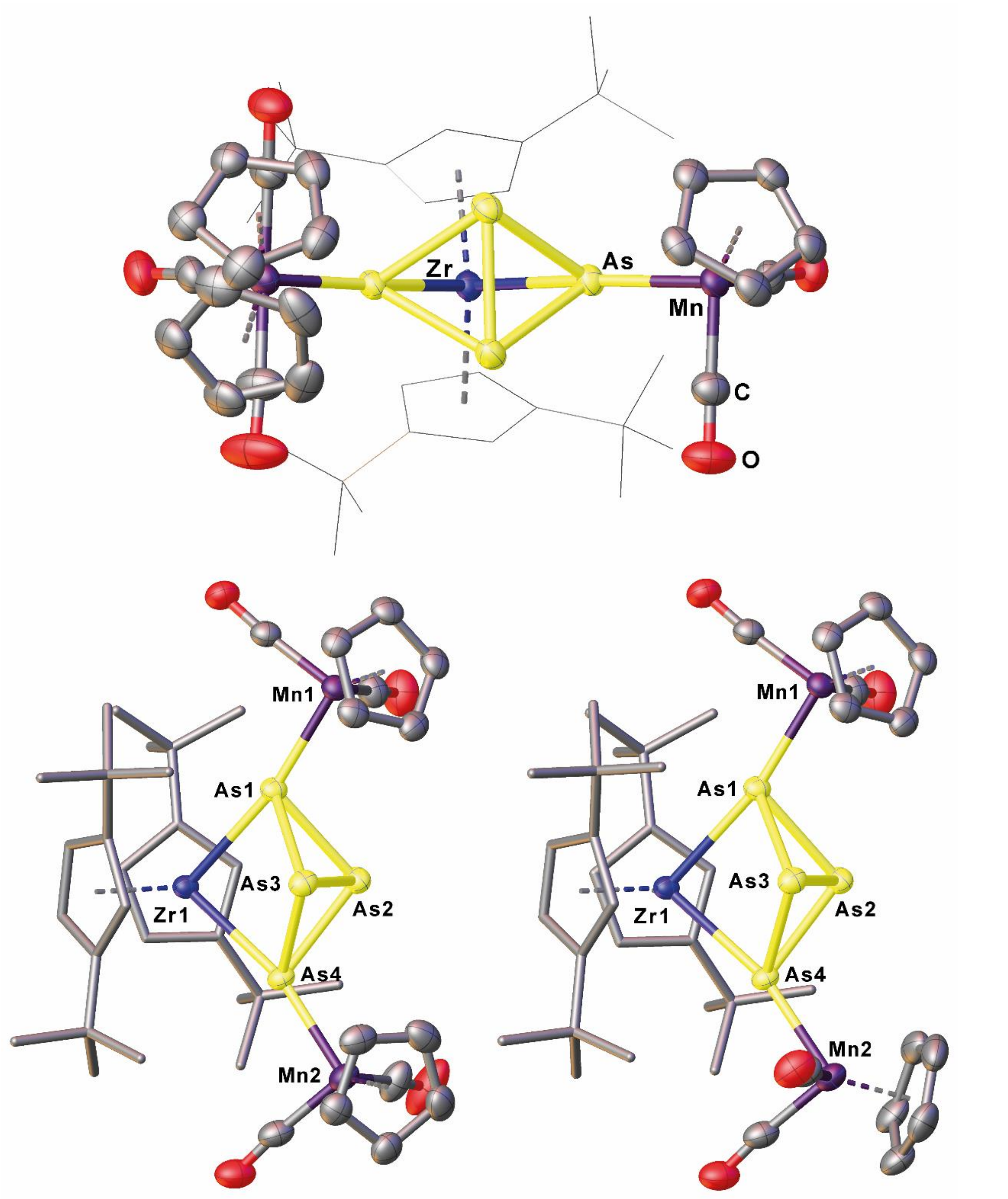


**Figure S2:** Molecular structure of **3** in the solid state (above). Thermal ellipsoids are depicted at 50% probability level. Hydrogen atoms are omitted and the Cp'' ligands are drawn in the wireframe model for clarity. The separate parts of the disorder are depicted below (left: part1, occupancy of 52%; right: part2, occupancy of 48%). Hydrogen atoms are omitted and the Cp'' ligands are drawn in the tube model for clarity. Selected bond lengths [Å] and angles [°]:Zr1-As4 2.6874(3), Zr1-As1 2.6837(3), As4-As2 2.4726(4), As4-As3 2.4666(4), As4-Mn2 2.3732(5), As1-As2 2.4710(4), As1-As3 2.4699(4), As1-Mn1

2.3605(4), As2-As3 2.4394(4); As1-Zr1-As4 85.871(9), As2-As4-Zr1 80.039(11), As3-As4-Zr1 80.228(10), As3-As4-As2 59.193(11), Mn2-As4-Zr1 166.512(16), Mn2-As4-As2 112.664(16), Mn2-As4-As3 109.772(15), As2-As1-Zr1 80.139(11), As3-As1-Zr1 80.242(10), As3-As1-As2 59.170(11), Mn1-As1-Zr1 167.600(15), Mn1-As1-As2 108.773(15), Mn1-As1-As3 111.582(15), As1-As2-As4 95.477(13), As3-As2-As4 60.282(11), As3-As2-As1 60.393(11), As4-As3-As1 95.656(12), As2-As3-As4 60.525(11), As2-As3-As1 60.437(11).

### [Cp''$_2$Zr(μ,η$^{1:1:1}$-As$_4$)(Fe(CO)$_4$)] (4):

Compound **4** crystallizes out of a concentrated *n*-pentane solution at -78 °C in form of orange plates in the monoclinic space group $P2_1/m$. The asymmetric unit contains half a molecule of **4**. One carbonyl ligand, which is located on a mirror plane, shows a disorder over three positions (occupancy of 0.6, 0.2 and 0.2). To describe the disorder the restrains ISOR and SIMU were used.

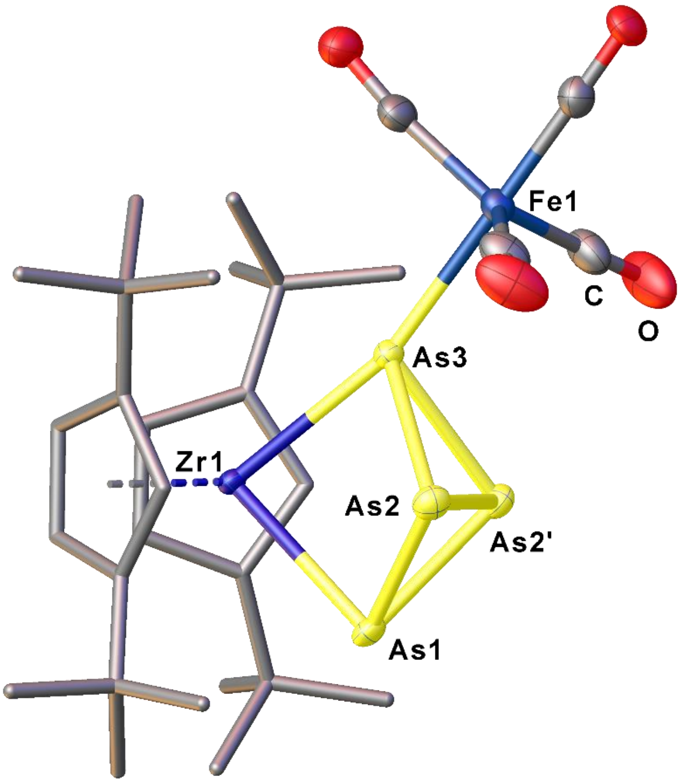


**Figure S3:** Molecular structure of **4** in the solid state. Thermal ellipsoids are depicted at 50% probability level. Hydrogen atoms are omitted and the Cp'' ligands are drawn in the tube model for clarity. Selected bond lengths [Å] and angles [°]: Zr1-As3 2.7078(4), Zr1-As1 2.6851(5), As3-As2 2.4791(4), As3-As2' 2.4791(4), As3-Fe1 2.4213(7), As1-As2 2.4457(4), As1-As2' 2.4457(4), As2-As2' 2.4321(6); As1-Zr1-As3 83.029(14), Fe1-As3-As2 110.125(19), Fe1-As3-As2' 110.125(19), As2'-As1-Zr1 83.508(13), As2-As1-Zr1 83.508(14)As2'-As1-As2 59.632(17), As1-As2-As3 93.072(14), As2$^{1}$-As2-As3 60.626(8), As2'-As2-As1 60.184(8).As2'-As3-Zr1 82.416(13), As2-As3-Zr1 82.416(13), As2-As3-As2' 58.748(16), Fe1-As3-Zr1 165.46(2).

**[Cp''$_2$Zr(μ,η$^{3:1:1}$-As$_4$)(Fe(CO)$_3$)] (5):**

Compound **5** crystallizes out of a concentrated *n*-pentane solution at -78 °C in form of violet plates in the orthorombic space group P*nma*. The asymmetric unit contains half a molecule of **5** and an only partly occupied pentane solvent molecule. Due to heavy disorder it was not possible to model the pentane solvent molecule. Therefore, a solvent mask was calculated and 162 electrons were found in a volume of 756 Å$^3$ in 1 void per unit cell. This is consistent with the presence of 0.45 pentane molecules per asymmetric unit, which account for 151 electrons per unit cell. Further, compound **5** co-crystallizes with compound **4**. This co-crystallization is presented by the disorder of an As atom and a Fe(CO)$_4$ fragment (0.9 to 0.1). However, due to the low occupancy (0.1) and the disordered pentane molecule (overlapping positions) it was not possible to properly locate the CO ligands of the Fe(CO)$_4$ fragment.

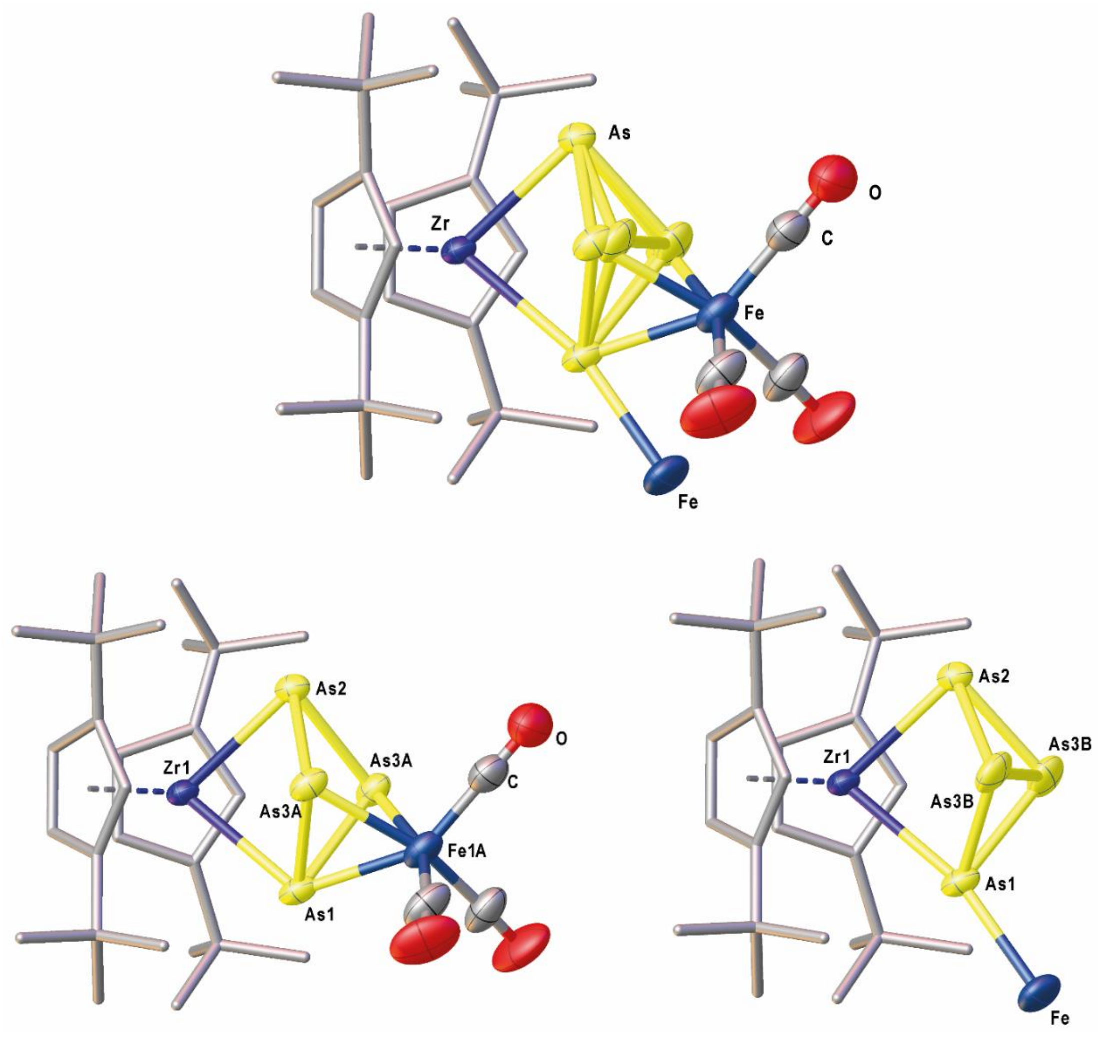


**Figure S4:** Molecular structure of **5** in the solid state. Thermal ellipsoids are depicted at 50% probability level. Hydrogen atoms are omitted and the Cp'' ligands are drawn in the wireframe model for clarity. Selected bond lengths [Å] and angles [°]: Zr1-As2 2.7105(8), Zr1-As1 2.7513(8), As2-As3A 2.4657(14), As2-As3B 2.377(14), As1-As3A 2.4721(13), As1-Fe1A 2.4265(15), As1-As3B 2.433(14), As3B-As3B$^1$ 2.391(18), As1-Fe1B 2.393(11), As3A-Fe1A 2.4151(16); As2-Zr1-As1 79.85(2), As3B$^1$-As2-Zr1 84.9(4), As3A-As1-Zr1 78.54(4), As3A-As1-As3A$^1$ 75.65(7), Fe1A-As1-Zr1 124.84(4), Fe1A-As1-As3A 59.07(4), Fe1A-As1-As3B 50.4(3), As3B-As1-Zr1 82.9(4), As3B$^1$-As1-As3B 58.9(5), Fe1B-As1-Zr1 165.8(3, Fe1B-As1-As3B 109.3(5), As2-As3A-As1 90.45(5), Fe1A-As3A-As2 99.36(5), Fe1A-As3A-As1 59.52(4), As3A-Fe1A-As1 61.40(4), As3A-Fe1A-As3A$^1$ 77.76(7), As2-As3B-As1 93.6(5), As2-As3B-As3B 59.8(2), As3B$^1$-As3B-As1 60.6(2), As3A-As2-Zr1 79.44(4), As3A$^1$-As2-As3A 75.88(6), As3B-As2-Zr1 84.9(4).

**[Cp''$_2$Zr(μ$_3$,η$^{1:1:1:1}$-As$_4$){AlMe$_3$}$_2$] (6a):**

Compound **6a** crystallizes out of a concentrated *n*-pentane solution at -30°C in form of orange blocks in the monoclinic space group *I*2/*a*. The asymmetric unit contains half a molecule of **6a**. The crystals of **6a** were twinned. Therefore, a refinement as a 2- component twin (BASF 0.57) was performed.

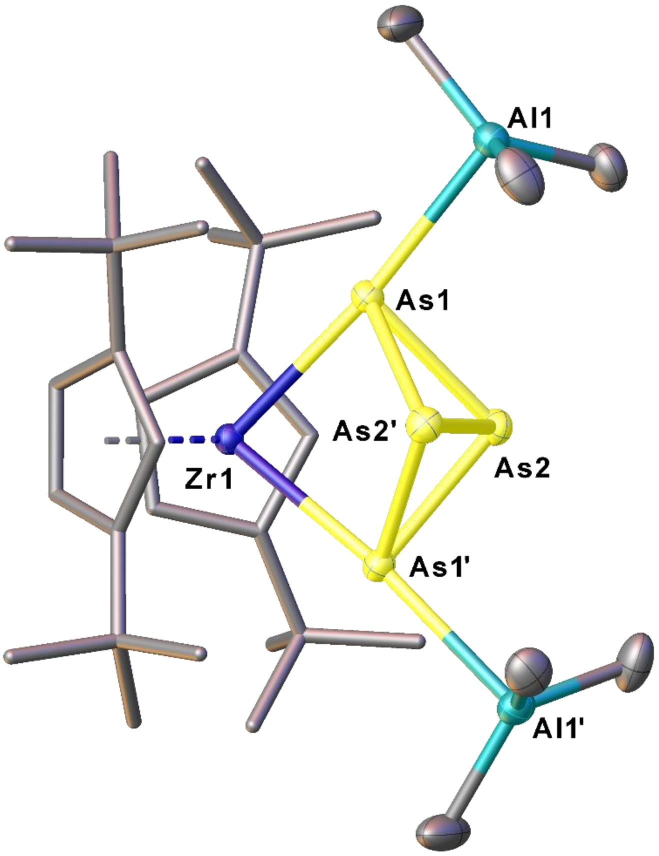


**Figure S5:** Molecular structure of **6a** in the solid state. Thermal ellipsoids are depicted at 50% probability level. Hydrogen atoms are omitted and the Cp'' ligands are drawn in the tube model for clarity. Selected bond lengths [Å] and angles [°]: Zr1-As1' 2.6810(2), Zr1-As1 2.6810(2), As1-As2 2.4513(2), As1-As2'- 2.4583(3), As1-Al1 2.6743(5), As2-As2' 2.4272(4); As1-Zr1-As1' 81.591(8), As2'-As1-Zr1 84.745(7), As2-As1-Zr1 84.881(7), As2-As1-As2' 59.258(9), As2-As1-Al1 100.118(13), As2'-As1-Al1 100.245(14), Al1-As1-Zr1 174.223(13), As1-As2-As1' 91.049(9), As2'-As2-As1' 60.225(8), As2'-As2-As1 60.515(8).

**[Cp''$_2$Zr($\mu_3$,$\eta^{1:1:1:1}$-As$_4$){AlEt$_3$}$_2$] (6b):**

Compound **6b** crystallizes out of a concentrated *n*-pentane solution at -30 °C in form of orange plates in the monoclinic space group *C*2/*c*. The asymmetric unit contains half a molecule of **6b**. The three ethyl substituents at AlEt$_3$ are disordered over two positions (occupancy 0.63:0.37; 0.63:0.37; 0.65:0.35). To describe the disorder of these ethyl substituents the restrains SADI and SIMU were used.

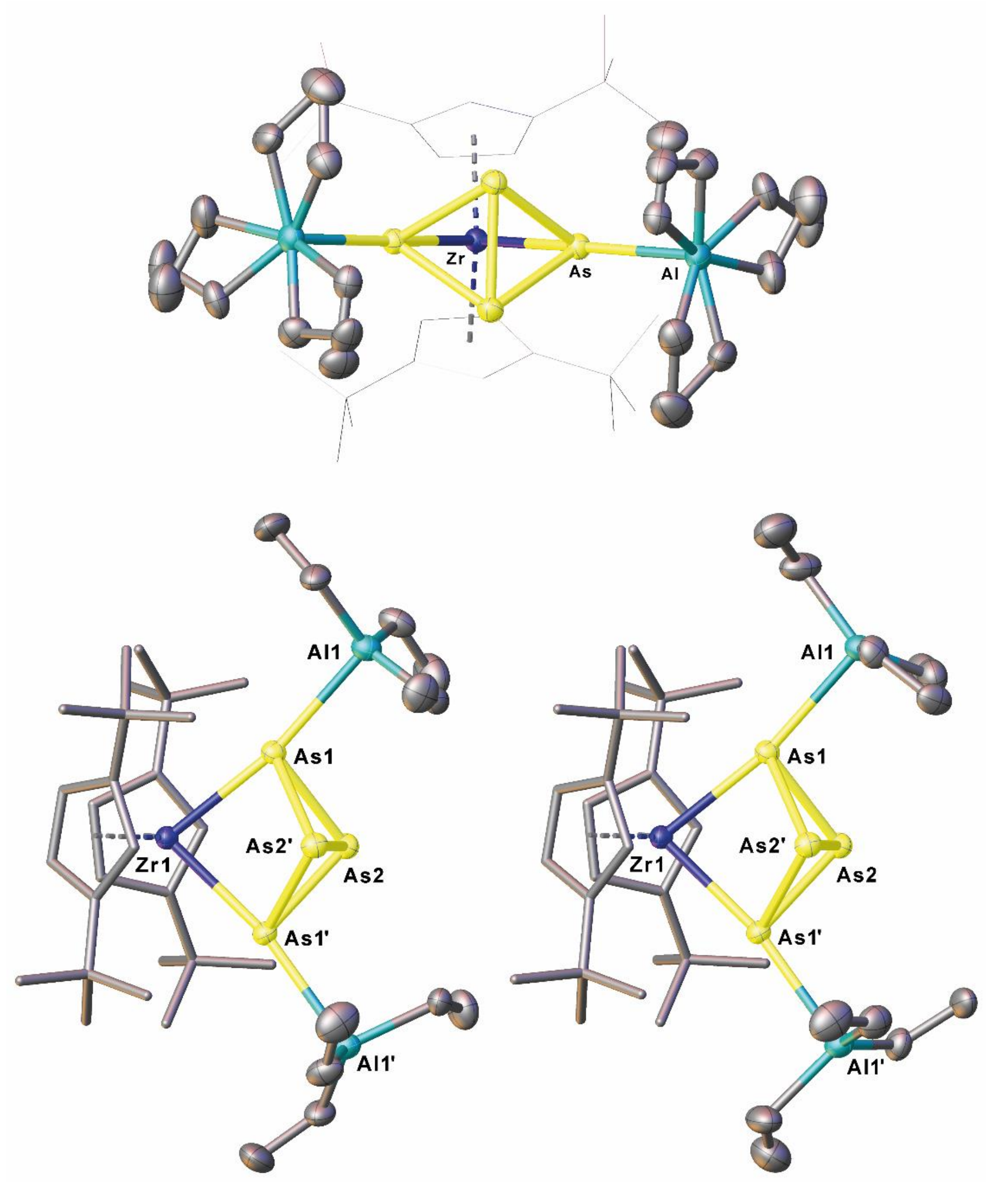


**Figure S6:** Molecular structure of **6b** in the solid state (above). Thermal ellipsoids are depicted at 50% probability level. Hydrogen atoms are omitted and the Cp'' ligands are drawn in the wireframe model for clarity. The separate parts of the disorder are depicted below (left: part1, occupancy of 63%; right: part2, occupancy of 37%). Selected bond lengths [Å] and angles [°]: Zr1-As1 2.7018(2), Zr1-As1' 2.7019(2), As1-As2' 2.4637(2), As1-As2 2.4583(2), As1-Al1 2.7415(5), As2-As2' 2.4202(3); As1-Zr1-As1' 81.216(8), Zr1-As1-Al1 170.087(13), As2'-As1-Zr1 84.937(7), As2-As1-Zr1 85.042(6),As2-As1-As2' 58.907(8), As2-As1-Al1 102.934(13), As2'-As1-Al1 104.161(13), As1-As2-As1' 91.218(8), As2'-As2-As1' 60.435(7), As2'-As2-As1 60.658(7).

**[Cp''$_2$Zr($\mu_3$,$\eta^{1:1:1:1}$-As$_4$){Al$^i$Bu$_3$}$_2$] (6c):**

Compound **6c** crystallizes out of a concentrated *n*-pentane solution at -30 °C in form of orange blocks in the monoclinic space group *C*2/*c*. The asymmetric unit contains half a molecule of **6c**. A $^i$Bu substituent of the Al$^i$Bu$_3$ ligand is disordered over two positions (occupancy of 0.86 and 0.14). To describe the disorder the restrains SADI and SIMU were used.

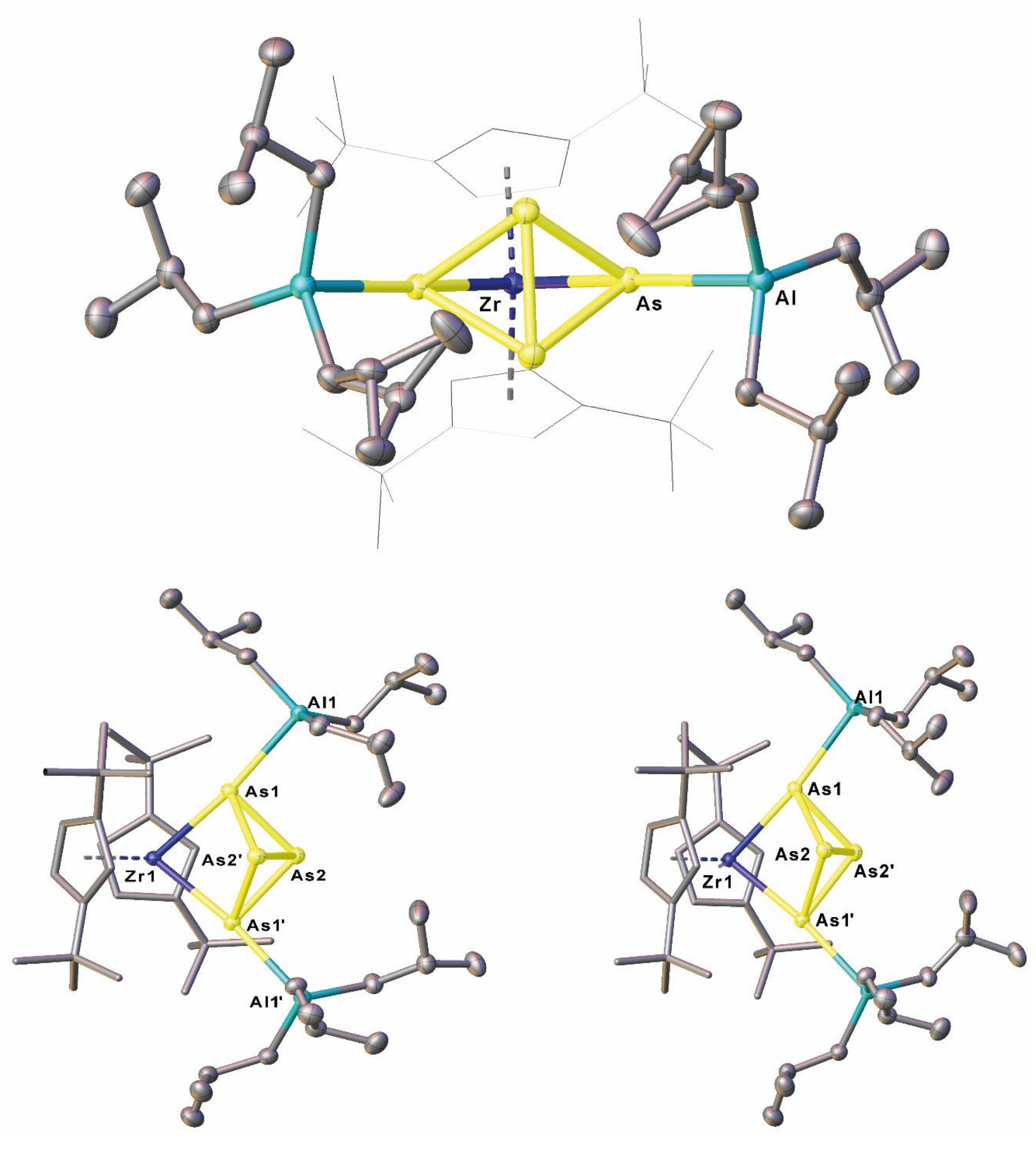


**Figure S7:** Molecular structure of **6c** in the solid state (above). Thermal ellipsoids are depicted at 50% probability level. Hydrogen atoms are omitted and the Cp'' ligands are drawn in the wireframe model for clarity. The separate parts of the disorder are depicted below (left: part1, occupancy of 86%; right: part2, occupancy of 14%). Selected bond lengths [Å] and angles [°]: Zr1-As1' 2.6810(3), Zr1-As1 2.6810(3), As1-As2' 2.4696(3), As1-As2 2.4550(3), As1-Al1 2.7566(6), As2-As2' 2.4281(4); As1'-Zr1-As1 83.462(11), Zr1-As1-Al1 172.833(15), As2'-As1-Zr1 82.784(8), As2-As1-Zr1 83.057(9), As2-As1-As2' 59.081(11), As2'-As1-Al1 104.082(15), As2-As1-Al1 102.142(15), As1-As2-As1' 92.896(11), As2'-As2-As1' 60.159(9), As2'-As2-As1 60.760(10).

**[Cp''$_2$Zr(μ,η$^{1:1:1}$-As$_4$)(B(C$_6$F$_5$)$_3$)] (7):**

Compound **7** crystallizes out of a concentrated *n*-hexane solution at -30 °C in form of clear orange blocks in the triclinic space group $P\bar{1}$. The asymmetric unit contains one molecule of **7**. The [Cp''$_2$Zr(μ,η$^{1:1}$-As$_4$)] fragment is disordered over two positions (occupancy of 0.77 and 0.23). To describe the disorder the restrains SADI, SIMU and ISOR were used.

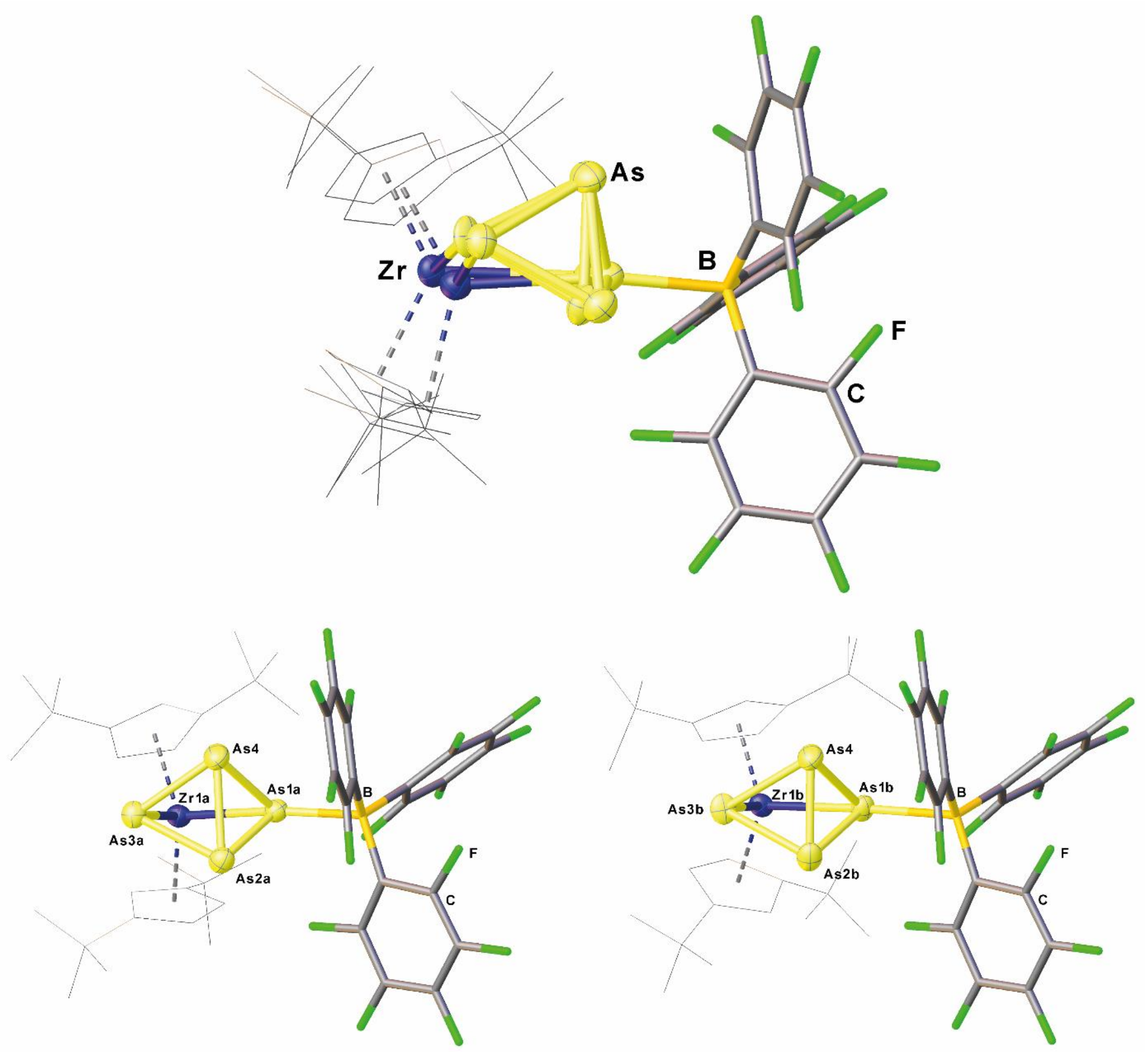


**Figure S8:** Molecular structure of **7** in the solid state (above). Thermal ellipsoids are depicted at 50% probability level. Hydrogen atoms are omitted, the Cp'' ligands are drawn in the wireframe model and the the [BC$_{18}$F$_{15}$] unit is drawn in the tube model for clarity. The separate parts of the disorder are depicted below (left: part1, occupancy of 77%; right: part2, occupancy of 23%). Selected bond lengths [Å] and angles [°]: As1A-As2A 2.457(4), As3A-As4 2.436(3), As3A-As2A 2.437(4), As4-As1A 2.445(3), As4-As2A 2.442(3), As4-As1B 2.552(9), As4-As2B 2.357(13), As4-As3B 2.543(12), As4-As3B 2.543(12), As1B-As2B 2.434(14), Zr1B-As1B 2.789(7), Zr1B-As3B 2.595(10), Zr1A-As3A 2.732(3), Zr1A-As1A 2.732(2), As1A-B1 2.177(6), B1-As1B 2.518(8); As1A-Zr1A-As3A 79.83(8), As2A-As3A-Zr1A 86.27(11), As2A-As4-As1A 60.36(10), As2A-As1A-Zr1A85.89(10), As3A-As2A-As4 59.89(10), As3A-As2A-As1A 91.51(11), As3A-As4-As1A 91.85(9), As3A-As4-As2A 59.96(10), As4-As2A-As1A 59.87(10), As4- As1A-Zr1A 83.69(7), As4-As1A-As2A 59.76(10), As4-As3A-Zr1A 83.86(10), As4-As3A-As2A 60.15(11), As2B-As4-As1B 59.3(3), As2B-As4-As3B 59.5(4), As3B-As4-As1B 85.3(3), As4-As1B-Zr1B 86.7(3), As2B-As1B-As4 56.4(4), As2B-As1B-B1 94.8(3), As2B-As1B-Zr1B 85.8(4), As4-As2B-As1B 64.4(4), As4-As2B-As3B 64.1(4), As1B-As2B-As3B 90.3(4), As4-As3B-Zr1B 91.2(4), As2B-As3B-As4 56.4(4), As2B-As3B-Zr1B 90.2(5), B1-As1B-As4 95.9(3), B1-As1A-As4 109.00(18), B1-As1A-As2A 101.90(17).

**Table S1:** Structure determination summary of the complexes **2**, **3** and **4**.

| **Compound** | **2** | **3** | **4** |
|---|---|---|---|
| Formula | $C_{36}H_{42}As_4O_{10}W_2Zr$ | $C_{40}H_{52}As_4Mn_2O_4Zr$ | $C_{30}H_{42}As_4FeO_4Zr$ |
| CCDC number | 2077256 | 2077257 | 2077258 |
| $D_{calc.}$/ g cm$^{-3}$ | 2.151 | 1.752 | 1.764 |
| $m$/mm$^{-1}$ | 15.466 | 7.938 | 10.399 |
| Formula Weight | 1393.29 | 1097.59 | 913.38 |
| Colour | red | violet | clear light orange |
| Shape | block | block | plate |
| Size/mm$^3$ | 0.15×0.07×0.03 | 0.25×0.19×0.10 | 0.27×0.19×0.09 |
| $T$/K | 123(1) | 122.99(11) | 123.01(10) |
| Crystal System | triclinic | triclinic | monoclinic |
| Space Group | $P$-1 | $P$-1 | $P2_1/m$ |
| $a$/Å | 11.0989(3) | 11.5684(3) | 9.1335(2) |
| $b$/Å | 12.5065(3) | 12.1340(4) | 17.1283(3) |
| $c$/Å | 17.5582(5) | 16.8248(4) | 11.3330(2) |
| α/° | 101.764(2) | 80.103(2) | 90 |
| β/° | 95.394(2) | 89.979(2) | 104.114(2) |
| γ/° | 113.207(2) | 63.838(2) | 90 |
| V/Å$^3$ | 2151.41(10) | 2080.60(11) | 1719.43(6) |
| $Z$ | 2 | 2 | 2 |
| $Z'$ | 1 | 1 | 0.5 |
| Wavelength/Å | 1.54178 | 1.39222 | 1.54184 |
| Radiation type | Cu$K_a$ | Cu $K_a$ | Cu $K_a$ |
| $\theta_{min}$/° | 3.986 | 2.416 | 4.022 |
| $\theta_{max}$/° | 66.565 | 74.751 | 72.087 |
| Measured Refl's. | 36658 | 46763 | 10794 |
| Ind't Refl's | 7562 | 11522 | 3482 |
| Refl's with I≥σ(I) | 7021 | 10758 | 3418 |
| $R_{int}$ | 0.0320 | 0.0415 | 0.0264 |
| Parameters | 490 | 535 | 217 |
| Restraints | 0 | 0 | 42 |
| Largest Peak | 1.615 | 0.806 | 1.429 |
| Deepest Hole | -0.984 | -1.049 | -0.906 |
| GooF | 1.068 | 1.065 | 1.127 |
| $wR_2$ (all data) | 0.0730 | 0.0853 | 0.0670 |
| $wR_2$ | 0.0711 | 0.0834 | 0.0666 |
| $R_1$ (all data) | 0.0297 | 0.0346 | 0.0259 |
| $R_1$ | 0.0270 | 0.0323 | 0.0253 |

**Table S2:** Structure determination summary of the complexes **5**, **6a** and **6b**.

| **Compound** | **5** | **6a** | **6b** |
|---|---|---|---|
| Formula | $C_{33.2}H_{52.8}As_4FeO_{2.7}Zr$ | 0.5·($C_{32}H_{60}Al_2As_4Zr$) | 0.5·($C_{38}H_{72}Al_2As_4Zr$) |
| CCDC number | 2077259 | 2077260 | 2077261 |
| $D_{calc.}$/ g cm$^{-3}$ | 1.594 | 1.537 | 1.465 |
| $m$/mm$^{-1}$ | 9.098 | 4.969 | 4.361 |
| Formula Weight | 941.90 | 889.66 | 973.81 |
| Colour | dark violet | dark orange | orange |
| Shape | plate-shaped | block | plate |
| Size/mm$^3$ | 0.25×0.20×0.14 | 0.20×0.11×0.08 | 0.25×0.21×0.12 |
| $T$/K | 123(1) | 122.99(11) | 123.00(11) |
| Crystal System | orthorhombic | monoclinic | monoclinic |
| Space Group | *Pnma* | *I*2/*a* | *C*2/*c* |
| $a$/Å | 12.0528(5) | 17.6443(3) | 25.1728(4) |
| $b$/Å | 17.2716(7) | 9.95170(16) | 10.3746(2) |
| $c$/Å | 18.8597(6) | 23.1782(4) | 18.0895(3) |
| α/° | 90 | 90 | 90 |
| β/° | 90 | 109.1757(19) | 110.797(2) |
| γ/° | 90 | 90 | 90 |
| V/Å$^3$ | 3926.0(3) | 3844.08(12) | 4416.40(14) |
| $Z$ | 4 | 4 | 4 |
| $Z'$ | 0.5 | 0.5 | 0.5 |
| Wavelength/Å | 1.54184 | 1.39222 | 1.39222 |
| Radiation type | Cu $K_a$ | Cu K | Cu K |
| $\theta_{min}$/° | 3.470 | 3.646 | 3.392 |
| $\theta_{max}$/° | 71.632 | 74.104 | 74.810 |
| Measured Refl's. | 11708 | 9433 | 22870 |
| Ind't Refl's | 3893 | 9433 | 6087 |
| Refl's with I≥σ(I) | 3256 | 8609 | 5789 |
| $R_{int}$ | 0.0392 | 0.0752 | 0.0304 |
| Parameters | 202 | 188 | 264 |
| Restraints | 2 | 0 | 19 |
| Largest Peak | 1.294 | 1.497 | 0.806 |
| Deepest Hole | -0.660 | -0.897 | -0.529 |
| GooF | 1.030 | 1.018 | 1.064 |
| $wR_2$ (all data) | 0.1108 | 0.1192 | 0.0631 |
| $wR_2$ | 0.1051 | 0.1175 | 0.0620 |
| $R_1$ (all data) | 0.0501 | 0.0430 | 0.0256 |
| $R_1$ | 0.0408 | 0.0407 | 0.0242 |

**Table S3:** Structure determination summary of the complexes **6c** and **7**.

| **Compound** | **6c** | **7** |
|---|---|---|
| Formula | 0.5·($C_{50}H_{96}Al_2As_4Zr$) | $C_{44}H_{42}As_4BF_{15}Zr$ |
| CCDC number | 2077262 | 2077263 |
| $D_{calc.}$/ g cm$^{-3}$ | 1.343 | 1.823 |
| *m*/mm$^{-1}$ | 3.465 | 6.020 |
| Formula Weight | 1142.12 | 1257.48 |
| Colour | orange | clear orange |
| Shape | block | block |
| Size/mm$^3$ | 0.22×0.17×0.10 | 0.21×0.14×0.11 |
| *T*/K | 123.00(10) | 123.00(10) |
| Crystal System | monoclinic | triclinic |
| Space Group | *C2/c* | *P*-1 |
| *a*/Å | 18.5668(4) | 11.2797(4) |
| *b*/Å | 11.9157(2) | 12.7421(5) |
| *c*/Å | 27.2938(5) | 18.5702(6) |
| α/° | 90 | 71.815(3) |
| β/° | 110.737(2) | 87.410(3) |
| γ/° | 90 | 65.217(4) |
| V/Å$^3$ | 5647.2(2) | 2290.82(16) |
| *Z* | 4 | 2 |
| *Z'* | 0.5 | 1 |
| Wavelength/Å | 1.39222 | 1.54184 |
| Radiation type | Cu $K_a$ | Cu $K_a$ |
| $\theta_{min}$/° | 3.127 | 2.517 |
| $\theta_{max}$/° | 64.688 | 73.366 |
| Measured Refl's. | 20223 | 28611 |
| Ind't Refl's | 6481 | 8705 |
| Refl's with I≥σ(I) | 5866 | 7895 |
| $R_{int}$ | 0.0282 | 0.0456 |
| Parameters | 280 | 880 |
| Restraints | 34 | 371 |
| Largest Peak | 0.804 | 0.610 |
| Deepest Hole | -0.456 | -0.814 |
| GooF | 1.049 | 1.136 |
| $wR_2$ (all data) | 0.0662 | 0.1158 |
| $wR_2$ | 0.0642 | 0.1134 |
| $R_1$ (all data) | 0.0335 | 0.0502 |
| $R_1$ | 0.0289 | 0.0457 |

## 2. NMR Investigations

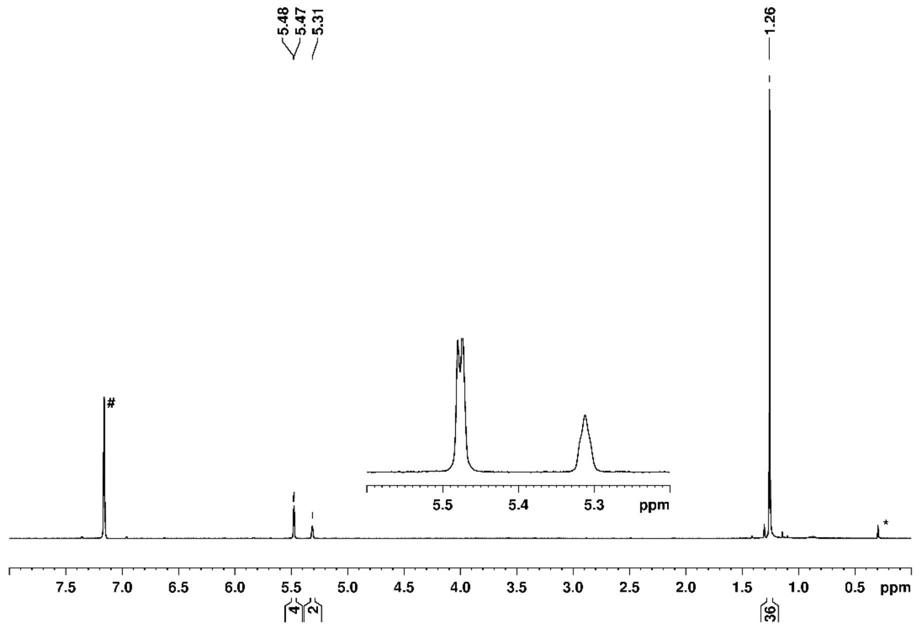


**Figure S9:** $^{1}H$ NMR spectrum of **2** at 293 K in $C_6D_6$ (#). The signal marked with * is due to silicon grease.

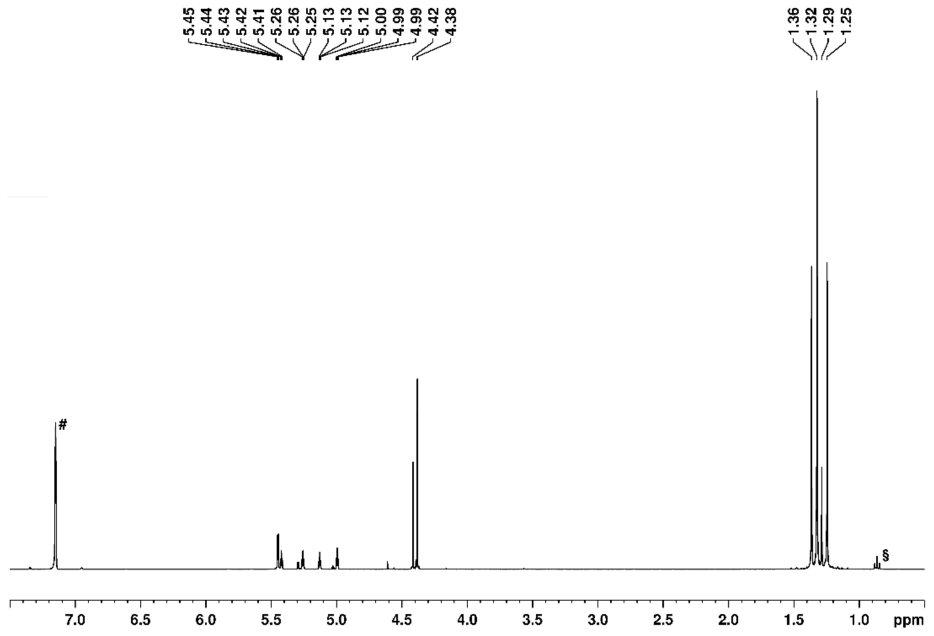


**Figure S10:** $^{1}H$ NMR spectrum of **3** at 293 K in $C_6D_6$ (#). The signal marked with § is due to *n*-pentane. A signal corresponding to the free $CpMn(CO)_2$ fragment could not be detected in the $^{1}H$ NMR, probably due to a highly dynamic behavior or a triplet spin state.

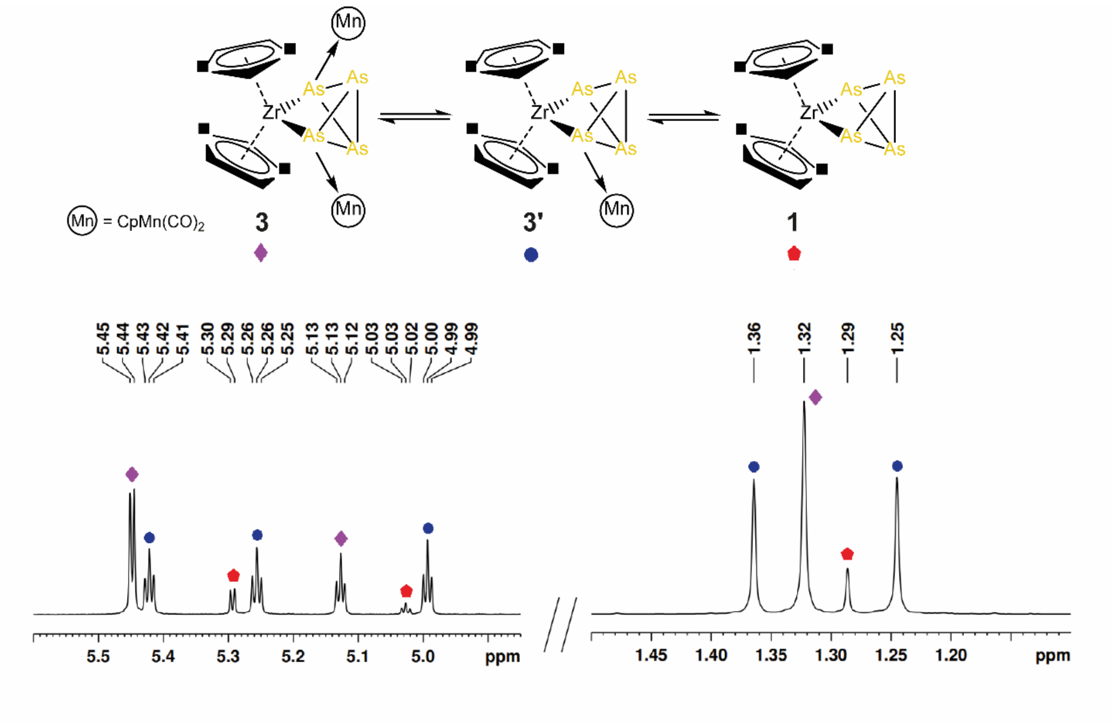


**Figure S11:** Parts of the $^{1}$H NMR spectrum of **3** at 293 K in $C_6D_6$.

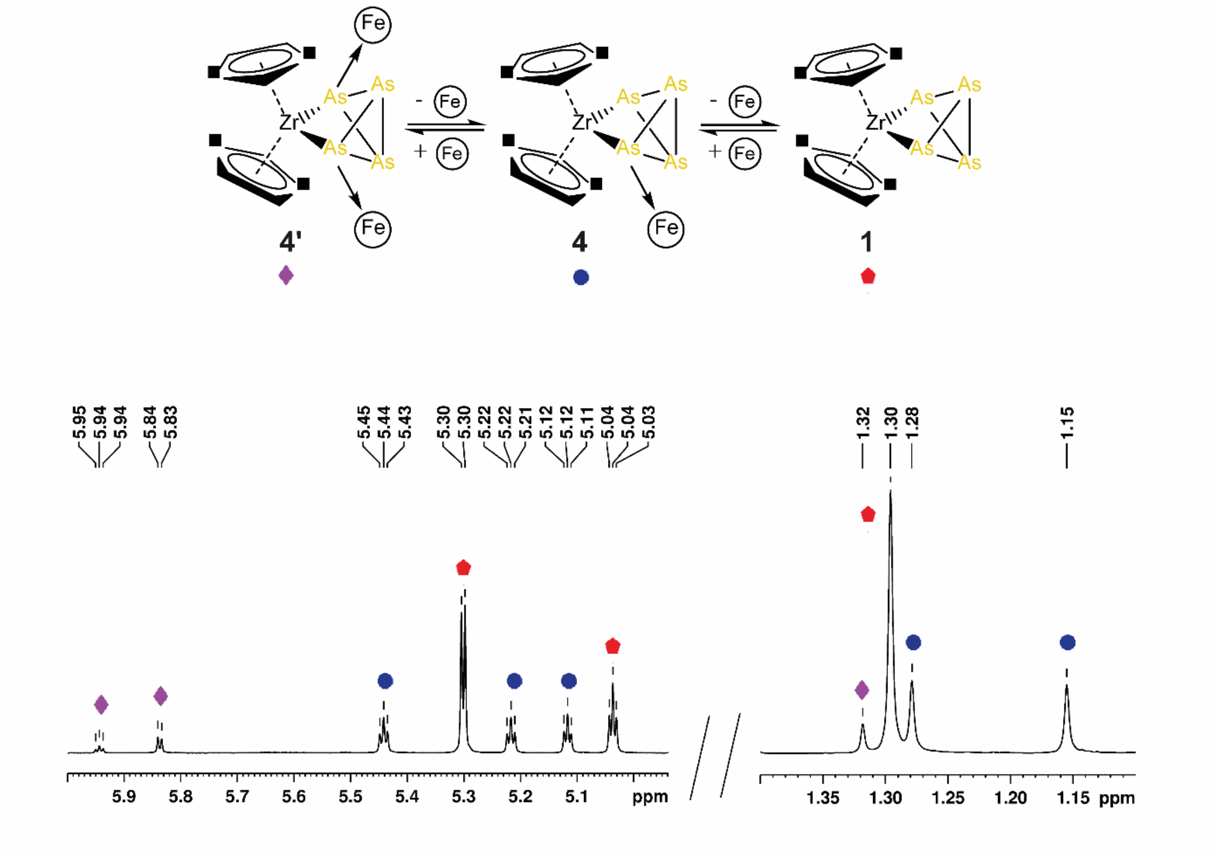


**Figure S12:** Parts of the $^{1}$H NMR spectrum of the reaction solution of **1** with $[Fe_2(CO)_9]$ at 293 K in $C_6D_6$.

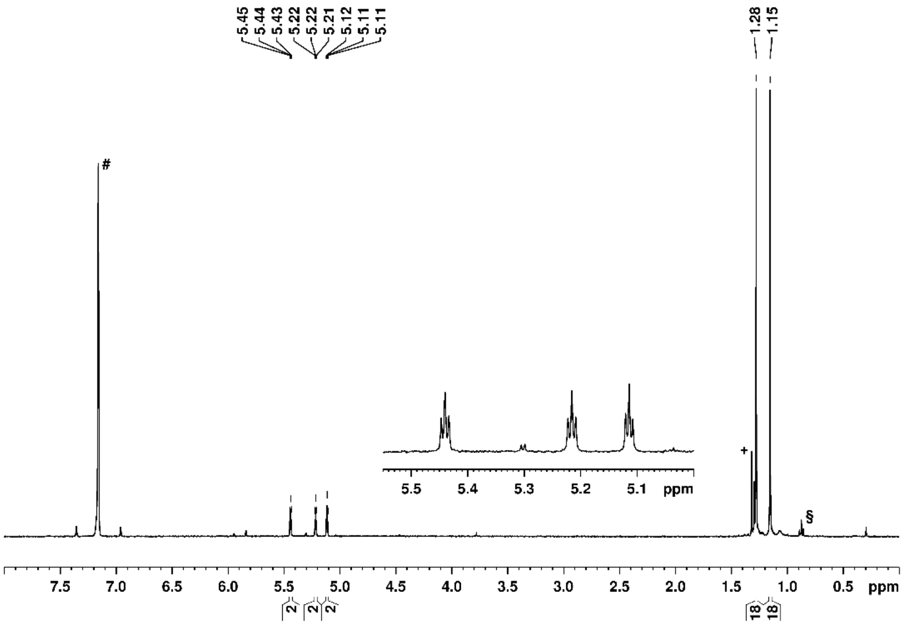


**Figure S13:** $^1$H NMR spectrum of **4** at 293 K in $C_6D_6$ (#). The signal marked with § is due to *n*-pentane, the signal marked with + due to a small amount of starting material **1**.

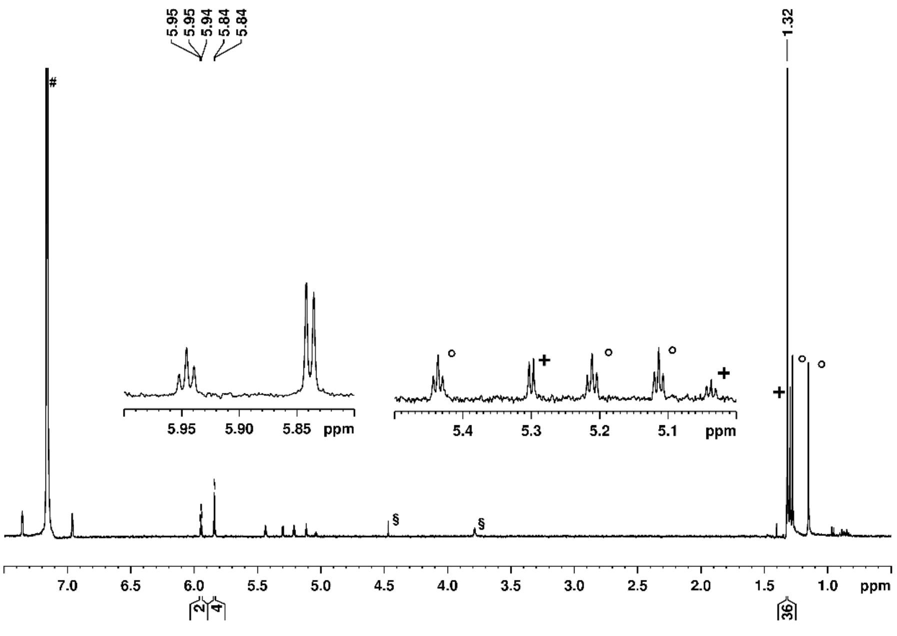


**Figure S14:** $^1$H NMR spectrum of **5** at 293 K in $C_6D_6$ (#). The signal marked with § are due to an unknown impurity, the signal marked with + due to a small amount of starting material **1** and the signals marked with ° due to [Cp''$_2$Zr(μ,η$^{1:1:1}$-As$_4$)(Fe(CO)$_4$)] (**4**). Especially the signals for **4** were expected to appear in the NMR spectrum due to the co-crystallization of **5** and **4**. The symmetric environment of the Cp'' ligand in **5** might be an indication of an on the NMR timescale fast dynamic process in solution. Possibly, the $Fe(CO)_3$ fragment migrates around the $As_4$-cycle.

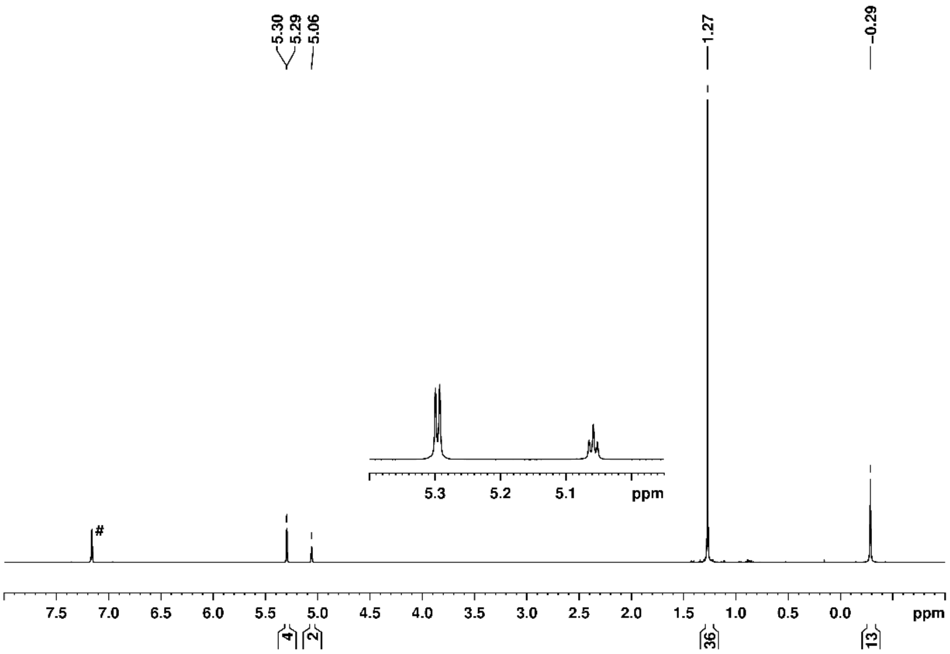


**Figure S15:** $^1$H NMR spectrum of **6a** at 293 K in $C_6D_6$ (#).

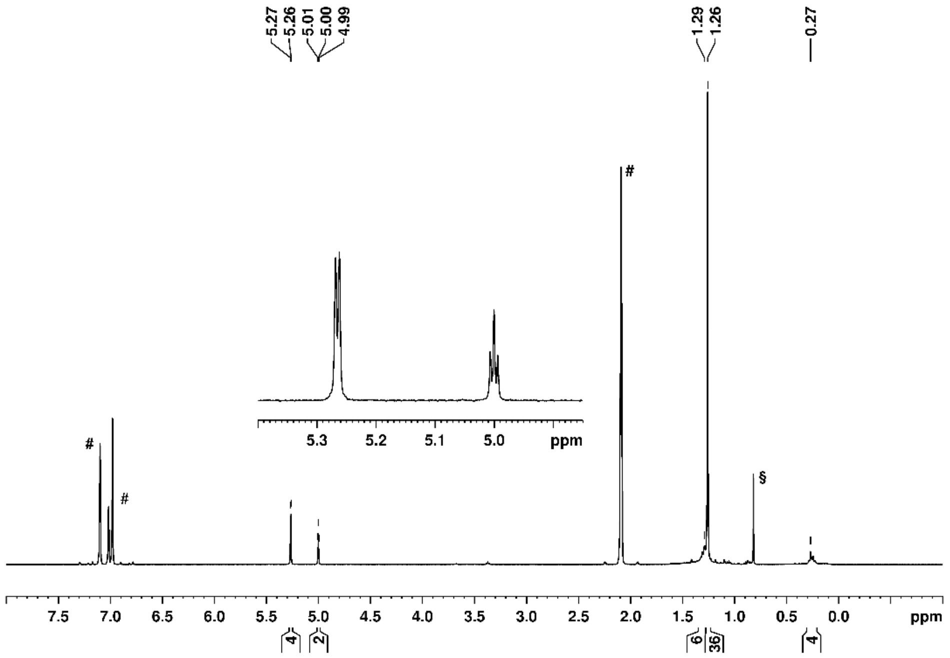


**Figure S16:** $^1$H NMR spectrum of **6b** at 293 K in tol-$d^8$ (#). The signal marked with § is due to free ethane.

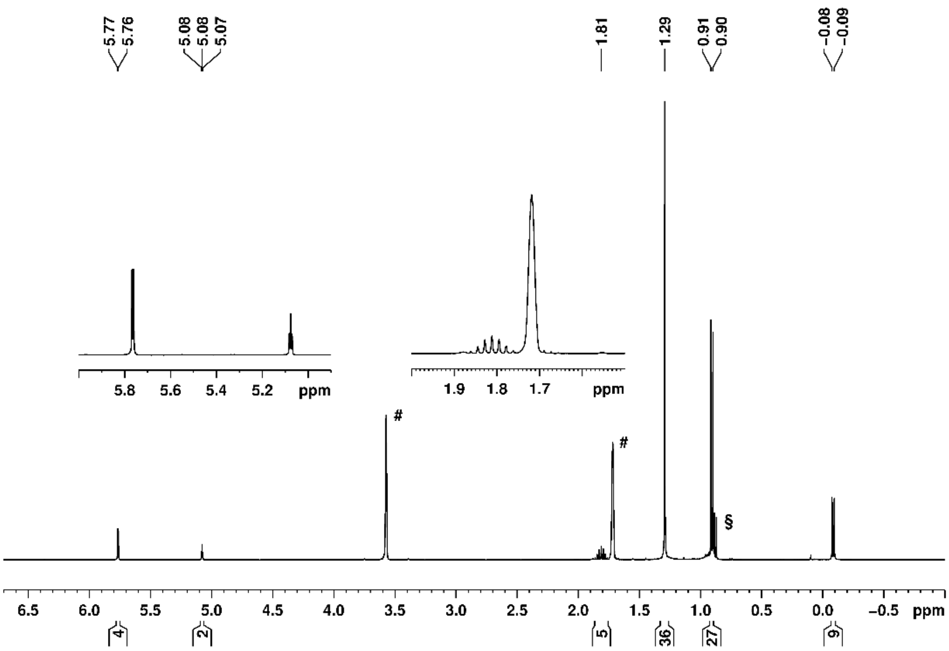


**Figure S17:** $^{1}H$ NMR spectrum of **6c** at 293 K in thf-d$^{8}$ (#). The signal marked with § is due to an unknown impurity.

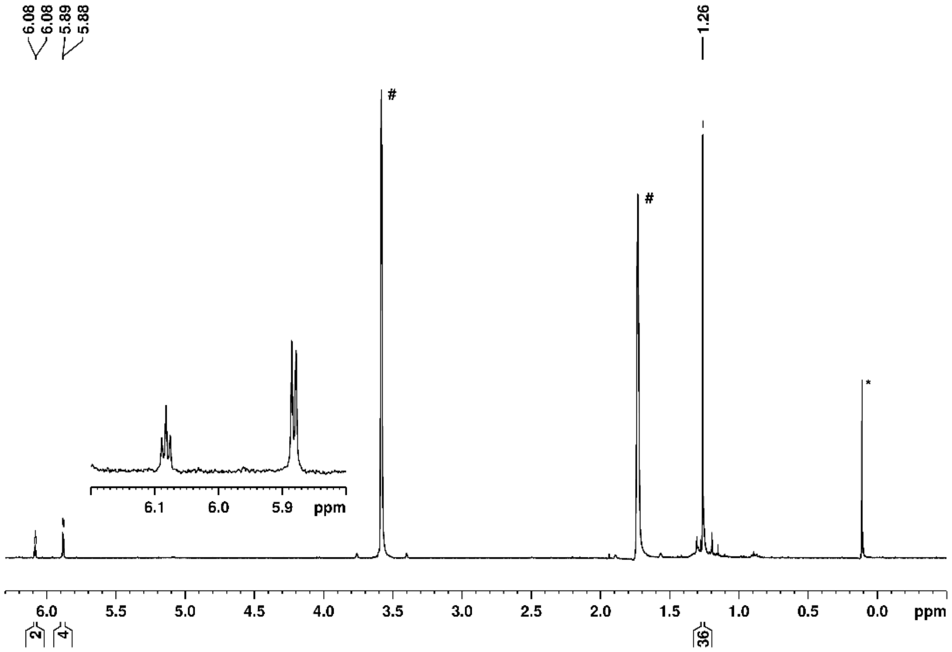


**Figure S18:** $^{1}H$ NMR spectrum of **7** at 293 K in thf-$d_8$ (#). The signal marked with * is due to silicon grease.

## 3. Computational Details

The geometry of the molecules has been optimized using Gaussian 09 program package.[5] Density functional theory (DFT) in form of Becke's three-parameter hybrid functioncal B3LYP[6] with def2-SVP all electron basis set[7] or def2-TZVP basis set for all atoms[8] was employed. The figures for the supporting information concerning the DFT calculations were created with Chemcraft.[9] The reaction energies have been calculated as single point calculations at the B3LYP/def2-TZVP level usind the B3LYP/def2SVP optimised geometries. The dispersion effects have been included in the single point calculations via the D3(BJ) model.[10] For the calculation of relative energies and reaction energies the SCF energies have been used witouth corrections for the zero point vibration energies.

Despite several attempts, the geometry optimization of the isomer of **7** in which the $B(C_6F_5)_3$ group is bonded to the bridgehead arsenic atoms did not reach convergence, indicating that this isomer is not a stable entity.

**Table S4:** Total and relative energies calculated at the B3LYP/def2-SVP level for the Isomers I, II, I_2 and II_2. Labeling according to figure S19.

| | Total energy [Ha] | | |
|---|---|---|---|
| **Compound** | **Isomer I** | **Isomer II** | **ΔE (Isomer I – II) [kJ·mol⁻¹]** |
| [Cp''$_2$Zr($\mu_3$,$\eta^{1:1:1:1}$-$As_4$)($W(CO)_5$)$_2$] | -11272.0985236 | -11272.0856656 | -33.76 |
| [Cp''$_2$Zr($\mu_3$,$\eta^{1:1:1:1}$-$As_4$)($CpMn(CO)_2$)$_2$] | -13146.7853601 | -13146.7796608 | -14.96 |
| [Cp''$_2$Zr($\mu$,$\eta^{1:1:1:1}$-$As_4$)($Fe(CO)_4$)$_2$] | -13438.4133113 | -13438.4001335 | -34.60 |
| [Cp''$_2$Zr($\mu_3$,$\eta^{1:1:1:1}$-$As_4$){$AlMe_3$}$_2$] | -10729.2667023 | -10729.2540650 | -33.18 |
| [Cp''$_2$Zr($\mu_3$,$\eta^{1:1:1:1}$-$As_4$){$AlEt_3$}$_2$] | -10964.9386234 | -10964.9386234 | 0.00 |
| [Cp''$_2$Zr($\mu_3$,$\eta^{1:1:1:1}$-$As_4$){$Al^iBu_3$}$_2$] | -11436.35712 | -11436.35027 | -17.98 |
| | | | |
| | **Total energy [Ha]** | | |
| **Compound** | **Isomer I_2** | **Isomer II_2** | **ΔE (Isomer I_2 – II_2) [kJ·mol⁻¹]** |
| [Cp''$_2$Zr($\mu_3$,$\eta^{1:1:1}$-$As_4$)($W(CO)_5$)] | -10638.6304544 | -10638.6231044 | -19.30 |
| [Cp''$_2$Zr($\mu_3$,$\eta^{1:1:1}$-$As_4$)($CpMn(CO)_2$)] | -11575.9729414 | -11575.9702229 | -7.14 |
| [Cp''$_2$Zr($\mu$,$\eta^{1:1:1}$-$As_4$)($Fe(CO)_4$)] | -11721.7858591 | -11721.7789285 | -18.20 |
| [Cp''$_2$Zr($\mu$,$\eta^{3:1:1}$-$As_4$)($Fe(CO)_3$)] | -11608.5858271 | n.a. | n.a. |
| [Cp''$_2$Zr($\mu_3$,$\eta^{1:1:1}$-$As_4$){$AlMe_3$}] | -10367.2130152 | -10367.2066613 | -16.68 |
| [Cp''$_2$Zr($\mu_3$,$\eta^{1:1:1}$-$As_4$){$AlEt_3$}] | -10485.0492434 | -10485.0442703 | -13.06 |
| [Cp''$_2$Zr($\mu_3$,$\eta^{1:1:1}$-$As_4$){$Al^iBu_3$}] | -10720.75877 | -10720.75552 | -8.53 |

**Table S5:** Reaction energies (kJ·mol⁻¹) for selected transformations at the B3LYP-D3(BJ)/def2TZVP level of theory.

| **Transformation** | **Reaction energy [kJ·mol⁻¹]** |
|---|---|
| **1** + [$W(CO)_5$(thf)] = [Cp''$_2$Zr($\mu_3$,$\eta^{1:1:1}$-$As_4$)($W(CO)_5$] (**I_2**)+ thf | -57.87 |
| [Cp''$_2$Zr($\mu_3$,$\eta^{1:1:1}$-$As_4$)($W(CO)_5$] (**I_2)** + [$W(CO)_5$(thf)] = **2** + thf | -58.52 |
| **1** + [$CpMn(CO)_2$(thf)] = [Cp''$_2$Zr($\mu_3$,$\eta^{1:1:1}$-$As_4$)($CpMn(CO)_2$] (**I_2**)+ thf | -61.32 |
| [Cp''$_2$Zr($\mu_3$,$\eta^{1:1:1}$-$As_4$)($CpMn(CO)_2$] (**I_2**) + [$CpMn(CO)_2$(thf)] = **3** + thf | -68.44 |
| **1** + $Fe_2(CO)_9$ = **4** + $Fe(CO)_5$ | -104.79 |
| **4** + $Fe_2(CO)_9$ = [Cp''$_2$Zr($\mu$,$\eta^{1:1:1:1}$-$As_4$)($Fe(CO)_4$)$_2$] (**I**) + $Fe(CO)_5$ | -99.52 |

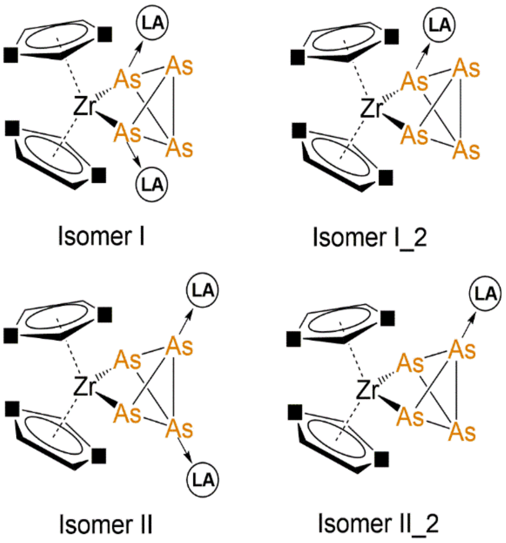


**Figure S19:** Labeling scheme of different isomers.

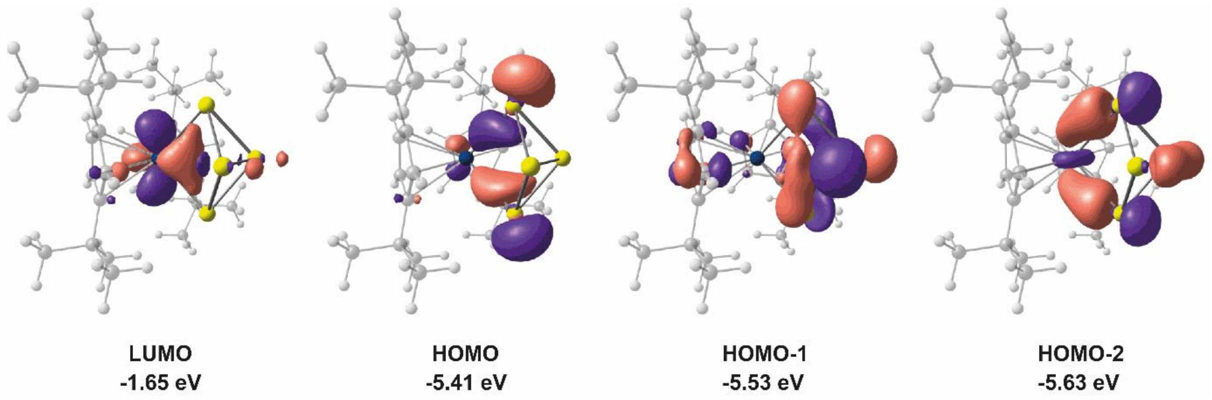


**Figure S20:** Selected molecular orbitals of **1** at the B3LYP/def2-TZVP level.

**Table S6:** Cartesian coordinates of the optimized geometry of [Cp''$_2$Zr($\eta^{1:1}$-As$_4$)] (**1**) at the B3LYP/def2-TZVP level of theory.

| | | | |
|---|---|---|---|
| Zr | 0.000172299 | -0.000051182 | -0.534015379 |
| As | -0.202191999 | 1.812317119 | 1.474037605 |
| As | 0.201814891 | -1.811817190 | 1.474666876 |
| As | -1.207864073 | -0.137994761 | 2.670128421 |
| As | 1.207571264 | 0.138940746 | 2.670096242 |
| C | -3.459146723 | -3.192126687 | -2.463524235 |
| H | -4.354209150 | -2.631496995 | -2.188345006 |
| H | -3.719919244 | -4.252653537 | -2.502186189 |
| H | -3.160838178 | -2.878796176 | -3.465349378 |
| C | 1.095432641 | 3.767436752 | -1.888609122 |
| H | 0.726555796 | 3.438175688 | -2.861941299 |
| H | 1.358015017 | 4.823776598 | -1.980089369 |
| H | 0.281208500 | 3.685550986 | -1.170108758 |
| C | 3.459511178 | 3.191483769 | -2.463895484 |
| H | 4.354564302 | 2.630960055 | -2.188485524 |
| H | 3.720246434 | 4.252011656 | -2.502762003 |
| H | 3.161336848 | 2.877923515 | -3.465678765 |
| C | -3.384728177 | 2.021343212 | -0.798817511 |
| C | 2.321121362 | 2.958194481 | -1.440675313 |
| C | -2.650786125 | 3.350776604 | -1.023534349 |
| H | -2.245443522 | 3.425189519 | -2.033718615 |
| H | -1.831399341 | 3.479180855 | -0.316945108 |
| H | -3.342710995 | 4.184630226 | -0.886428361 |
| C | -2.516744152 | 0.804878606 | -1.123052439 |
| C | -2.320858795 | -2.958629809 | -1.440189195 |

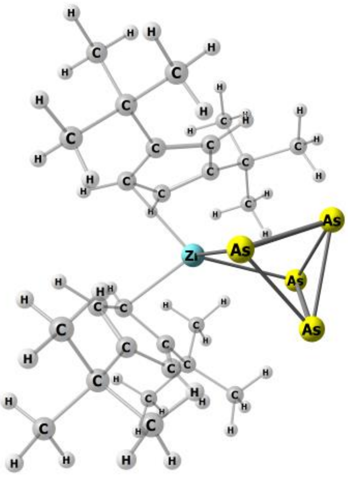

| | | | |
|---|---|---|---|
| C | -4.584893727 | 1.962731784 | -1.776635147 |
| H | -5.266652520 | 2.795031021 | -1.585068538 |
| H | -5.142233016 | 1.032082620 | -1.656914928 |
| H | -4.253474799 | 2.025773341 | -2.814425510 |
| C | 2.650557023 | -3.351128554 | -1.022151276 |
| H | 1.831079665 | -3.479023715 | -0.315543280 |
| H | 3.342273842 | -4.185087059 | -0.884605599 |
| H | 2.245231947 | -3.425975564 | -2.032306939 |
| C | 2.794290311 | 3.474929900 | -0.074666115 |
| H | 2.038213547 | 3.335417869 | 0.698416706 |
| H | 3.009943820 | 4.543535842 | -0.139405423 |
| H | 3.709206749 | 2.976416350 | 0.249383980 |
| C | 1.719141685 | -0.656767461 | -2.288374926 |
| H | 1.445322828 | -1.446904084 | -2.968189247 |
| C | 2.050386994 | 1.455100881 | -1.429236660 |
| C | -3.938944606 | 1.986590232 | 0.632603916 |
| H | -4.595054343 | 2.844856589 | 0.792350557 |
| H | -3.145678789 | 2.031256766 | 1.377426429 |
| H | -4.529874030 | 1.087440043 | 0.815188747 |
| C | -1.718778982 | 0.656155071 | -2.288516619 |
| H | -1.444822692 | 1.446220477 | -2.968364987 |
| C | -1.448206183 | -0.719971122 | -2.482104722 |
| H | -0.931079787 | -1.135671676 | -3.330874152 |
| C | 1.448610400 | 0.719319329 | -2.482240472 |
| H | 0.931509076 | 1.134849525 | -3.331109784 |
| C | 3.384752482 | -2.021744307 | -0.798003758 |
| C | -2.794264799 | -3.475085652 | -0.074155013 |
| H | -2.038430018 | -3.335211241 | 0.699095857 |
| H | -3.009673294 | -4.543756069 | -0.138659470 |
| H | -3.709357633 | -2.976676975 | 0.249533271 |
| C | -2.050146393 | -1.455544109 | -1.429048056 |
| C | 2.516984615 | -0.805282993 | -1.122807477 |
| C | 2.667471332 | 0.496330853 | -0.584194469 |
| H | 3.229520506 | 0.734534032 | 0.302678558 |
| C | -2.667289718 | -0.496642001 | -0.584229130 |
| H | -3.229372071 | -0.734658431 | 0.302676532 |
| C | -1.095162078 | -3.767990970 | -1.887791601 |
| H | -0.726156288 | -3.438957383 | -2.861146951 |
| H | -1.357760313 | -4.824347310 | -1.979069015 |
| H | -0.281028602 | -3.685968533 | -1.169214740 |
| C | 4.584968341 | -1.963741538 | -1.775802307 |
| H | 5.266466498 | -2.796189517 | -1.584010125 |
| H | 5.142574785 | -1.033234211 | -1.656360186 |

**Table S7:** Cartesian coordinates of the optimized geometry of the Isomers for [Cp''$_2$Zr($\mu_3$,$\eta^{1:1:1:1}$-As$_4$)(W(CO)$_5$)$_2$] at the B3LYP/def2-SVP level of theory.

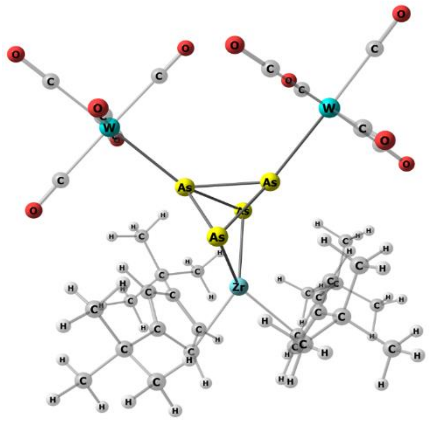

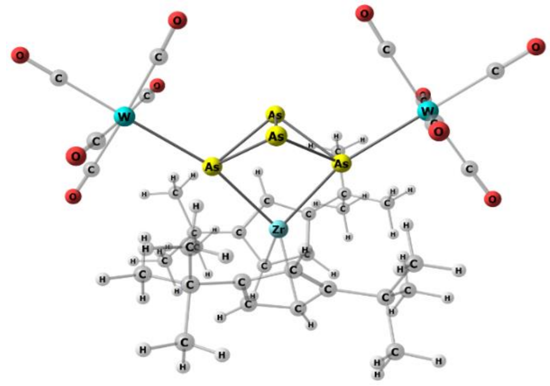

| | | | |
|---|---|---|---|
| C | -3.623021232 | -2.093698411 | -1.162577195 |
| C | -2.775015675 | -2.575784066 | -0.120563953 |
| H | -1.906638381 | -3.211442662 | -0.275359669 |
| C | -3.314914039 | -2.230438204 | 1.153984018 |
| C | -4.470016707 | -1.438065744 | 0.890076604 |
| H | -5.151172143 | -1.040450294 | 1.640039169 |
| C | -4.664159457 | -1.366587545 | -0.518250483 |
| H | -5.516579104 | -0.902413768 | -1.008912780 |
| C | -3.642112185 | -2.572673445 | -2.617924112 |
| C | -2.254908762 | -3.021804820 | -3.109651598 |
| H | -1.530696576 | -2.191970003 | -3.122003738 |
| H | -2.329467551 | -3.404332114 | -4.139805145 |
| H | -1.836991067 | -3.830466816 | -2.491968898 |
| C | -4.590820787 | -3.799852330 | -2.657332656 |
| H | -4.226820247 | -4.603730632 | -1.998914733 |

| | | | |
|---|---|---|---|
| C | 1.308287934 | 2.975629559 | -2.066968166 |
| C | 0.392908168 | 2.044051240 | -2.645480424 |
| H | 0.681794889 | 1.171094425 | -3.224665754 |
| C | -0.944805782 | 2.493170909 | -2.463610401 |
| C | -0.860874624 | 3.667916948 | -1.656790397 |
| H | -1.700282570 | 4.294379523 | -1.360381963 |
| C | 0.511724815 | 3.973431559 | -1.440543543 |
| H | 0.884820006 | 4.866708722 | -0.944524688 |
| C | 2.802141285 | 3.100216087 | -2.378513889 |
| C | 3.383120298 | 1.780124611 | -2.908895757 |
| H | 3.279670252 | 0.967554143 | -2.176680749 |
| H | 4.456267406 | 1.897323285 | -3.123338984 |
| H | 2.893540674 | 1.462586262 | -3.841951412 |
| C | 2.928910802 | 4.171371520 | -3.493266246 |
| H | 2.357607435 | 3.882792910 | -4.389344027 |

| | | | |
|---|---|---|---|
| H | -4.653819005 | -4.201453707 | -3.681893281 |
| H | -5.608127879 | -3.530773250 | -2.332964026 |
| C | -4.196055618 | -1.493178560 | -3.564856186 |
| H | -5.216639222 | -1.186645193 | -3.286343781 |
| H | -4.243073783 | -1.879557048 | -4.595215154 |
| H | -3.560435921 | -0.596802148 | -3.570295588 |
| C | -3.005830345 | -2.924044789 | 2.487331184 |
| C | -3.252257791 | -2.010906466 | 3.701228237 |
| H | -3.114764096 | -2.580828453 | 4.633576134 |
| H | -4.275828807 | -1.606188436 | 3.714373450 |
| H | -2.547541749 | -1.166000459 | 3.723845506 |
| C | -3.985544447 | -4.125123826 | 2.572606891 |
| H | -3.845206963 | -4.812678423 | 1.724095414 |
| H | -5.034221616 | -3.789392019 | 2.565967129 |
| H | -3.813499483 | -4.691637700 | 3.502332031 |
| C | -1.570286739 | -3.470663498 | 2.550658508 |
| H | -1.356138517 | -4.167406739 | 1.726109716 |
| H | -1.418778350 | -4.023864764 | 3.490418410 |
| H | -0.822272345 | -2.665688788 | 2.522819159 |
| C | -3.493904058 | 2.341903327 | 1.159098501 |
| C | -2.612051678 | 2.770320767 | 0.121485476 |
| H | -1.702674483 | 3.345327179 | 0.280996087 |
| C | -3.167156734 | 2.463275267 | -1.156433850 |
| C | -4.371693600 | 1.746165814 | -0.898838239 |
| H | -5.074700246 | 1.396482546 | -1.652649235 |
| C | -4.576348668 | 1.684517316 | 0.508201950 |
| H | -5.458459937 | 1.273721447 | 0.993877007 |
| C | -3.495401487 | 2.829308150 | 2.612164965 |
| C | -4.342938484 | 4.129179128 | 2.629488497 |
| H | -3.903573923 | 4.898183936 | 1.975434562 |
| H | -4.391975329 | 4.539418144 | 3.651397188 |
| H | -5.372495146 | 3.941177120 | 2.287166132 |
| C | -4.149212429 | 1.805809504 | 3.557776488 |
| H | -5.184825853 | 1.575008873 | 3.262220252 |
| H | -4.186739976 | 2.207491332 | 4.582675913 |
| H | -3.584542167 | 0.863398884 | 3.587888631 |
| C | -2.085379761 | 3.169130502 | 3.124669468 |
| H | -1.434692832 | 2.281789554 | 3.162246832 |
| H | -2.146704720 | 3.571323864 | 4.148197150 |
| H | -1.587519878 | 3.930727410 | 2.506522511 |
| C | -2.815619313 | 3.144719423 | -2.485917989 |
| C | -3.740045177 | 4.388638025 | -2.576057973 |
| H | -3.579578532 | 5.064582049 | -1.721822036 |
| H | -4.802609921 | 4.100211389 | -2.584083047 |
| H | -3.531587003 | 4.952119856 | -3.500191012 |
| C | -3.092247180 | 2.248020758 | -3.705767144 |
| H | -2.924515053 | 2.816052603 | -4.634333942 |
| H | -4.132037850 | 1.887518932 | -3.727347876 |
| H | -2.424950055 | 1.373336180 | -3.729301757 |
| C | -1.357573577 | 3.628646847 | -2.533622683 |
| H | -0.644567883 | 2.793551521 | -2.492886608 |
| H | -1.124364608 | 4.318785046 | -1.708679867 |
| H | -1.170035885 | 4.172282049 | -3.472358971 |
| C | 4.249470415 | 4.191177185 | 0.189473338 |
| C | 1.458600430 | 4.314273741 | 1.021842437 |
| C | 2.178700248 | 3.812412330 | -1.771582669 |
| C | 3.830202508 | 1.547420016 | -0.980438646 |
| C | 3.205841247 | 2.084358861 | 1.874440163 |
| C | 2.817960711 | -2.561343004 | -2.008377433 |
| C | 3.820219055 | -1.794371295 | 0.681876975 |
| C | 2.006079671 | -3.714310594 | 1.909824425 |
| C | 3.810902561 | -4.577038609 | -0.176162591 |
| C | 0.961662951 | -4.466487094 | -0.721749247 |
| As | -0.629195078 | 0.082227542 | 1.825429385 |
| As | 0.585542241 | 1.224032031 | -0.026762076 |
| As | 0.510559573 | -1.209802571 | 0.025114809 |
| As | -0.630570044 | -0.003749969 | -1.826661554 |
| O | 5.153160664 | 4.902372543 | 0.275561793 |
| O | 0.766651752 | 5.072670661 | 1.543858675 |
| O | 1.935570221 | 4.311126814 | -2.778471134 |
| O | 4.459322394 | 0.785440555 | -1.563241802 |
| O | 3.508948842 | 1.626764641 | 2.881260893 |
| O | 3.052510800 | -2.257599650 | -3.089038773 |
| O | 4.601545950 | -1.066614320 | 1.100731028 |
| O | 1.804657797 | -4.045832129 | 2.991581221 |
| O | 4.615871355 | -5.398954802 | -0.255196761 |
| O | 0.146352410 | -5.198594553 | -1.078924205 |
| W | 2.670484219 | 2.945059429 | 0.048541403 |
| W | 2.400390923 | -3.141603135 | -0.046843326 |
| Zr | -2.699987205 | 0.101061743 | -0.002280301 |
| H | 3.984797116 | 4.289939647 | -3.785990870 |
| H | 2.557100024 | 5.151208947 | -3.155579913 |
| C | 3.608874012 | 3.567463246 | -1.153631824 |
| H | 3.245232000 | 4.533836113 | -0.769091825 |
| H | 4.668416957 | 3.699062347 | -1.421876566 |
| H | 3.565111868 | 2.833167286 | -0.337918719 |
| C | -2.155100747 | 2.089220894 | -3.313173216 |
| C | -3.480165675 | 2.162060333 | -2.535270525 |
| H | -4.324908214 | 1.947538036 | -3.207982923 |
| H | -3.648776226 | 3.161795507 | -2.107122971 |
| H | -3.516424842 | 1.428944877 | -1.717269466 |
| C | -2.217438097 | 3.123698306 | -4.469839125 |
| H | -1.283563090 | 3.126671139 | -5.053302102 |
| H | -2.383300503 | 4.143922165 | -4.090434934 |
| H | -3.045823180 | 2.874679548 | -5.152637297 |
| C | -1.984230779 | 0.693395454 | -3.936920636 |
| H | -1.106961143 | 0.650057896 | -4.600827523 |
| H | -2.867411827 | 0.438983978 | -4.541624363 |
| H | -1.873696050 | -0.088676940 | -3.174122326 |
| C | -1.308379858 | 2.975366928 | 2.067417336 |
| C | -0.393111386 | 2.043561031 | 2.645735668 |
| H | -0.682108098 | 1.170511208 | 3.224727028 |
| C | 0.944659454 | 2.492544643 | 2.463902930 |
| C | 0.860847096 | 3.667484107 | 1.657350631 |
| H | 1.700328571 | 4.293904063 | 1.361059829 |
| C | -0.511713078 | 3.973177760 | 1.441150969 |
| H | -0.884725463 | 4.866588736 | 0.945311585 |
| C | -2.802219910 | 3.100115226 | 2.378979851 |
| C | -2.928859006 | 4.171145451 | 3.493861914 |
| H | -2.357529418 | 3.882420988 | 4.389877400 |
| H | -3.984722910 | 4.289752096 | 3.786655903 |
| H | -2.557002544 | 5.150999112 | 3.156274881 |
| C | -3.608912002 | 3.567616105 | 1.154170961 |
| H | -3.245113227 | 4.533962597 | 0.769719546 |
| H | -4.668430176 | 3.699368275 | 1.422449607 |
| H | -3.565273670 | 2.833398647 | 0.338383209 |
| C | -3.383332488 | 1.780025152 | 2.909200558 |
| H | -3.279846013 | 0.967520149 | 2.176928854 |
| H | -4.456497895 | 1.897253396 | 3.123547062 |
| H | -2.893854952 | 1.462377983 | 3.842274072 |
| C | 2.154929834 | 2.088243917 | 3.313320438 |
| C | 2.217260754 | 3.122260537 | 4.470398931 |
| H | 1.283465214 | 3.124840043 | 5.053991159 |
| H | 2.382893921 | 4.142667623 | 4.091388210 |
| H | 3.045781576 | 2.873104055 | 5.152982929 |
| C | 3.479951601 | 2.161405984 | 2.535362232 |
| H | 4.324736803 | 1.946478718 | 3.207892984 |
| H | 3.648603857 | 3.161350479 | 2.107715618 |
| H | 3.516079987 | 1.428707546 | 1.716979493 |
| C | 1.984141279 | 0.692147876 | 3.936463485 |
| H | 1.874037380 | -0.089648729 | 3.173322570 |
| H | 1.106686299 | 0.648347172 | 4.600087231 |
| H | 2.867196722 | 0.437702234 | 4.541342598 |
| C | -5.737151399 | -2.803463466 | 0.148042064 |
| C | -5.256058280 | 0.014471102 | 0.291969314 |
| C | -3.830681503 | -1.838860507 | 2.089719797 |
| C | -2.953696204 | -3.400038248 | -0.251519127 |
| C | -4.353752935 | -1.522113585 | -2.020257081 |
| C | 4.353954536 | -1.522065240 | 2.020114023 |
| C | 2.953803564 | -3.399914908 | 0.251232506 |
| C | 3.830892264 | -1.838574175 | -2.089879609 |
| C | 5.737307560 | -2.803143774 | -0.148212321 |
| C | 5.255889780 | 0.014856572 | -0.291812574 |
| As | -1.792445392 | -0.116436301 | -0.039011522 |
| As | -0.028064161 | -1.348124398 | 1.232826211 |
| As | 0.028064962 | -1.347956770 | -1.233197116 |
| As | 1.792373074 | -0.116355745 | 0.038838149 |
| O | -6.688136733 | -3.454075966 | 0.218673975 |
| O | -5.936125379 | 0.933779490 | 0.431041303 |
| O | -3.703480506 | -1.936090424 | 3.228254957 |
| O | -2.370807327 | -4.375767889 | -0.412726850 |
| O | -4.544298090 | -1.460619675 | -3.153175987 |
| O | 4.544663582 | -1.460624491 | 3.153015604 |
| O | 2.370859710 | -4.375618994 | 0.412387333 |
| O | 3.703962306 | -1.935936444 | -3.228428887 |
| O | 6.688389733 | -3.453603400 | -0.218924955 |
| O | 5.935760882 | 0.934317245 | -0.430844186 |
| W | -4.079255832 | -1.667413312 | 0.032587094 |
| W | 4.079336182 | -1.667231863 | -0.032706568 |
| Zr | -0.000063389 | 1.979182804 | 0.000088225 |

**Table S8:** Cartesian coordinates of the optimized geometry of the Isomers for [Cp''$_2$Zr(μ,η$^{1:1:1}$-As$_4$)(W(CO)$_5$)] at the B3LYP/def2-SVP level of theory.

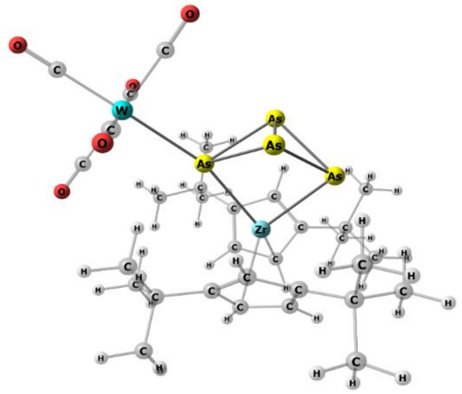

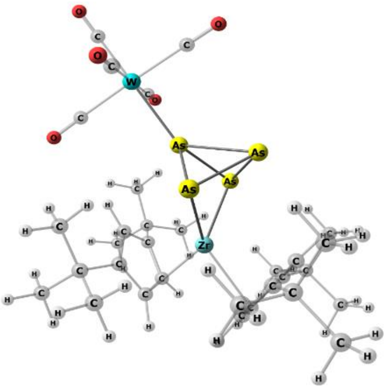

| | | | |
|---|---|---|---|
| W | 3.619195223 | -0.049942543 | -0.141373439 |
| Zr | -1.780662244 | 0.047172797 | 0.538952074 |
| As | 0.870168485 | 0.061166293 | -0.175518356 |
| As | -2.140949484 | -0.112005020 | -2.161842566 |
| As | 0.003141304 | 1.152061242 | -2.273771698 |
| As | 0.046238818 | -1.296845434 | -2.123553142 |
| O | 3.744636081 | 0.085372106 | -3.368415854 |
| O | 3.424871166 | -3.268774895 | -0.290225068 |
| O | 6.779389963 | -0.274698031 | -0.071152918 |
| O | 3.935211890 | 3.159097486 | -0.017951725 |
| O | 3.573949118 | -0.168179287 | 3.077833080 |
| C | -1.556826946 | -2.593791163 | 0.900638564 |
| H | -0.812149168 | -3.196930897 | 0.387147556 |
| C | -2.897811012 | -2.401243634 | 0.459473293 |
| C | -3.514358034 | -1.562261565 | 1.435218950 |
| H | -4.554651247 | -1.243223861 | 1.429987255 |
| C | -2.588931961 | -1.328905050 | 2.489380423 |
| H | -2.809732867 | -0.796003547 | 3.411697373 |
| C | -1.365375285 | -1.981555031 | 2.175872431 |
| C | -0.230386334 | -2.262478267 | 3.164127475 |
| C | 1.017744293 | -2.806524809 | 2.451825200 |
| H | 1.399996569 | -2.095353683 | 1.707204505 |
| H | 0.815460278 | -3.758949664 | 1.938663929 |
| H | 1.822515402 | -2.989368461 | 3.180334480 |
| C | 0.143778057 | -1.009677446 | 3.977188855 |
| H | 0.529728842 | -0.209650980 | 3.330637790 |
| H | 0.929058980 | -1.250666501 | 4.710440457 |
| H | -0.719003881 | -0.614783560 | 4.537072899 |
| C | -0.748714850 | -3.350278643 | 4.139842007 |
| H | -1.037316533 | -4.264790689 | 3.598478494 |
| H | -1.627018927 | -3.000127005 | 4.704189366 |
| H | 0.038185743 | -3.616803347 | 4.864234236 |
| C | -3.654757114 | -3.282756430 | -0.545194502 |
| C | -4.817859258 | -2.546027376 | -1.233014407 |
| H | -5.518989982 | -2.111718389 | -0.504315945 |
| H | -5.390511514 | -3.250783525 | -1.856543050 |
| H | -4.460092898 | -1.739243986 | -1.889563334 |
| C | -2.734718198 | -3.895409596 | -1.615926557 |
| H | -2.297631903 | -3.129918346 | -2.271447853 |
| H | -3.312005819 | -4.586761246 | -2.250155380 |
| H | -1.912818928 | -4.475802694 | -1.168951850 |
| C | -4.249477685 | -4.444802586 | 0.296036459 |
| H | -4.950653162 | -4.070279404 | 1.057916218 |
| H | -3.456944604 | -5.006715962 | 0.814210065 |
| H | -4.795545785 | -5.146699542 | -0.355644680 |
| C | -2.140652705 | 2.697040667 | 0.218198580 |
| H | -1.830497506 | 3.233137739 | -0.674757731 |
| C | -3.433372721 | 2.125260601 | 0.414948596 |
| C | -3.417143898 | 1.557621760 | 1.720789299 |
| H | -4.259431732 | 1.083630702 | 2.219820342 |
| C | -2.147183621 | 1.801757017 | 2.312535969 |
| H | -1.868761604 | 1.548754501 | 3.333943608 |
| C | -1.356815674 | 2.559338724 | 1.396874645 |
| C | -0.130925135 | 3.400854420 | 1.767356014 |
| C | 0.610140977 | 3.918849010 | 0.522595073 |
| H | 0.974085006 | 3.097971084 | -0.110017045 |
| H | 1.484399313 | 4.516294702 | 0.821889247 |
| H | -0.033825518 | 4.564957683 | -0.094177731 |
| C | -0.676394783 | 4.626993613 | 2.547419096 |
| H | -1.396056691 | 5.197259348 | 1.939438465 |
| H | 0.150779033 | 5.302919450 | 2.819902230 |
| H | -1.184973411 | 4.319800457 | 3.474492625 |
| C | 0.849092254 | 2.646995401 | 2.682764946 |
| H | 1.293312425 | 1.778264606 | 2.177560056 |
| C | -4.107431589 | -0.261356505 | -1.353806470 |
| C | -3.708413549 | -1.471718538 | -0.714126084 |
| H | -3.370397358 | -2.363417907 | -1.236250400 |
| C | -3.930193205 | -1.385397394 | 0.691406981 |
| C | -4.395707780 | -0.059244456 | 0.933807986 |
| H | -4.701569261 | 0.340577431 | 1.898707792 |
| C | -4.516572536 | 0.618476058 | -0.310909062 |
| H | -4.929431011 | 1.616356259 | -0.441067661 |
| C | -4.415642077 | -0.083454667 | -2.844409434 |
| C | -3.636701613 | -1.081052667 | -3.720248990 |
| H | -2.547737566 | -0.950209020 | -3.627661434 |
| H | -3.898889746 | -0.935069217 | -4.780014433 |
| H | -3.876476058 | -2.124855076 | -3.465440143 |
| C | -5.930842867 | -0.368645333 | -3.013931800 |
| H | -6.182841730 | -1.385508066 | -2.674584972 |
| H | -6.222192355 | -0.279767477 | -4.073588407 |
| H | -6.539795068 | 0.341064335 | -2.432655003 |
| C | -4.129427498 | 1.349184726 | -3.327543701 |
| H | -4.677750723 | 2.098903235 | -2.735476059 |
| H | -4.446411935 | 1.467121888 | -4.376151167 |
| H | -3.058339550 | 1.586770552 | -3.271624620 |
| C | -4.091563553 | -2.570548831 | 1.654344820 |
| C | -3.702837630 | -2.218775243 | 3.100802659 |
| H | -3.949318782 | -3.056726200 | 3.772097920 |
| H | -4.242971149 | -1.333647140 | 3.469743635 |
| H | -2.624082973 | -2.025961972 | 3.194216731 |
| C | -5.601127181 | -2.931307128 | 1.629436463 |
| H | -5.930370100 | -3.188611839 | 0.610588708 |
| H | -6.220317904 | -2.092091924 | 1.982388326 |
| H | -5.797836123 | -3.798606122 | 2.281076329 |
| C | -3.298387188 | -3.809909624 | 1.203105394 |
| H | -3.594395548 | -4.142950093 | 0.196351515 |
| H | -3.491501704 | -4.647189589 | 1.892300086 |
| H | -2.214627658 | -3.626432923 | 1.198468050 |
| C | -1.371703356 | 2.178260465 | 2.061483561 |
| C | -0.414200487 | 2.404483167 | 1.029080995 |
| H | 0.659229914 | 2.279111303 | 1.144653787 |
| C | -1.050965712 | 2.934843799 | -0.129203999 |
| C | -2.447239149 | 2.950945610 | 0.162544224 |
| H | -3.227644048 | 3.339802801 | -0.488978515 |
| C | -2.639788372 | 2.505677383 | 1.500659687 |
| H | -3.592338943 | 2.499021847 | 2.026561657 |
| C | -1.071218977 | 2.015421592 | 3.555547207 |
| C | -1.128384296 | 3.439506812 | 4.168532569 |
| H | -0.398590430 | 4.107493471 | 3.685222219 |
| H | -0.895071751 | 3.401816437 | 5.245323375 |
| H | -2.127073054 | 3.888537864 | 4.051634240 |
| C | -2.115676423 | 1.134459669 | 4.264151374 |
| H | -3.139387242 | 1.514047517 | 4.117708275 |
| H | -1.925815966 | 1.119011705 | 5.349272784 |
| H | -2.081414509 | 0.097085096 | 3.903164207 |
| C | 0.334904636 | 1.441807652 | 3.806136817 |
| H | 0.450331267 | 0.433585518 | 3.379957014 |
| H | 0.520918972 | 1.365723928 | 4.889078123 |
| H | 1.124028369 | 2.082064180 | 3.383598394 |
| C | -0.369207976 | 3.738688817 | -1.245436251 |
| C | -0.420236184 | 5.220612893 | -0.785513354 |
| H | 0.082391200 | 5.351929307 | 0.185286488 |
| H | -1.458133712 | 5.572800958 | -0.679014327 |
| H | 0.085851344 | 5.866389713 | -1.521910911 |
| C | -1.098566127 | 3.628410246 | -2.596233279 |
| H | -0.649555802 | 4.324858148 | -3.322025674 |
| H | -2.165484967 | 3.885704290 | -2.512665486 |
| H | -1.020802593 | 2.616099551 | -3.019759565 |

| | | | |
|---|---|---|---|
| H | 0.360240934 | 2.293449932 | 3.603479648 |
| H | 1.673032735 | 3.312262941 | 2.984881583 |
| C | -4.690946234 | 2.409154913 | -0.413531538 |
| C | -5.437102025 | 3.563595908 | 0.305296363 |
| H | -4.798787070 | 4.457057685 | 0.388746714 |
| H | -5.743986623 | 3.272829086 | 1.321965827 |
| H | -6.343121263 | 3.842932390 | -0.257354718 |
| C | -4.351314074 | 2.872343463 | -1.841321266 |
| H | -3.749903104 | 3.794393141 | -1.839283521 |
| H | -5.278407309 | 3.088381636 | -2.395536034 |
| H | -3.798197576 | 2.105835933 | -2.404726732 |
| C | -5.624754681 | 1.187738923 | -0.481304316 |
| H | -6.550107092 | 1.443979914 | -1.021556994 |
| H | -5.919189855 | 0.842359665 | 0.522504977 |
| H | -5.148486267 | 0.348089788 | -1.005689000 |
| C | 3.680267664 | 0.036743187 | -2.222571815 |
| C | 3.485997017 | -2.121392172 | -0.237359207 |
| C | 5.627607370 | -0.190875349 | -0.097470592 |
| C | 3.794368730 | 2.018048179 | -0.062854530 |
| C | 3.579461055 | -0.125712224 | 1.926560375 |
| C | 1.107108074 | 3.348461427 | -1.432968069 |
| H | 1.220310947 | 2.303522805 | -1.753650053 |
| H | 1.689361759 | 3.490282549 | -0.510009678 |
| H | 1.566521230 | 3.981331873 | -2.208357733 |
| C | 5.915352156 | -0.250347278 | -0.005171227 |
| C | 3.859048092 | -1.491855447 | 1.633176518 |
| C | 3.723507420 | 1.377047030 | 1.014895684 |
| C | 3.925923793 | 0.779115125 | -1.853699907 |
| C | 4.016604082 | -2.098192005 | -1.247515536 |
| As | -0.430434139 | -1.331005529 | 1.437237243 |
| As | 1.205778364 | -0.505365635 | -0.220635020 |
| As | -0.275033536 | -2.327714339 | -0.874502023 |
| As | -0.468298183 | -0.086850649 | -2.001431939 |
| O | 7.066410200 | -0.193154794 | 0.054527045 |
| O | 3.843503346 | -2.117529417 | 2.595633573 |
| O | 3.601844818 | 2.340199424 | 1.636108319 |
| O | 3.942637136 | 1.407645228 | -2.815549202 |
| O | 4.072780221 | -3.058742111 | -1.872487420 |
| W | 3.904547085 | -0.351473392 | -0.109655377 |
| Zr | -2.053828948 | 0.458525580 | 0.159742468 |

**Table S9:** Cartesian coordinates of the optimized geometry of the Isomers for [Cp''$_2$Zr($\mu_3$,$\eta^{1:1:1:1}$-As$_4$)(CpMn(CO)$_2$)$_2$] at the B3LYP/def2- SVP level of theory.

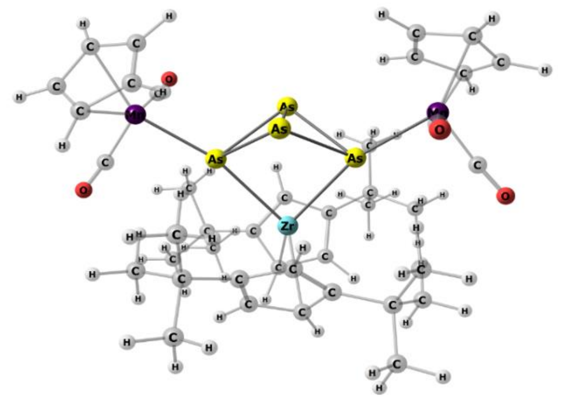

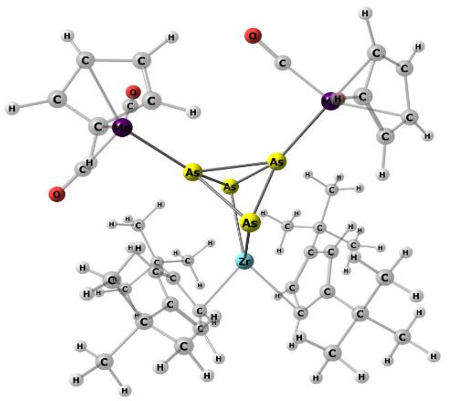

| | | | |
|---|---|---|---|
| Zr | 0.008041933 | 1.510709488 | -0.012297642 |
| As | -1.826715503 | -0.527014655 | -0.001777666 |
| As | 1.823167126 | -0.541723280 | 0.019012667 |
| As | 0.002583081 | -1.735545915 | -1.217404557 |
| As | -0.014925864 | -1.718154653 | 1.253047433 |
| Mn | 3.868500494 | -1.843580541 | -0.109693459 |
| Mn | -3.898986210 | -1.792437401 | 0.141343503 |
| O | 5.654295525 | 0.487139023 | -0.253336333 |
| O | -5.648723553 | 0.555805147 | 0.379632828 |
| O | 3.640339796 | -2.178941680 | -3.021805497 |
| C | 0.354874212 | 1.588356951 | -2.680664933 |
| H | 0.654957003 | 0.723000201 | -3.266314406 |
| C | -0.311478012 | 1.640597874 | 2.654898292 |
| H | -0.596879736 | 0.784477141 | 3.261331297 |
| C | 3.323580603 | -3.868956459 | 0.531758941 |
| H | 2.455724580 | -4.425839926 | 0.183992836 |
| C | -1.230590178 | 2.574002665 | 2.085294907 |
| C | -0.988763472 | 2.006388515 | -2.470006111 |
| C | 1.026261808 | 2.067373727 | 2.421174576 |
| C | 2.257581891 | 1.678134073 | 3.248278976 |
| C | 1.259469613 | 2.541135953 | -2.118813507 |
| C | 0.452564869 | 3.520710669 | -1.478710288 |
| H | 0.816011395 | 4.421862328 | -0.990362545 |
| C | -0.915283015 | 3.184476425 | -1.667013699 |
| H | -1.762105999 | 3.789441413 | -1.349203787 |
| C | 3.572860643 | 1.945681910 | 2.498076514 |
| H | 3.656235371 | 1.331208157 | 1.592092779 |
| H | 4.432070364 | 1.703537338 | 3.143225434 |
| H | 3.674330026 | 3.002212937 | 2.207104774 |
| C | 2.228352939 | 0.214512649 | 3.720145271 |
| H | 1.300826636 | -0.028499944 | 4.261044828 |
| H | 3.066151307 | 0.030798592 | 4.411685922 |
| H | 2.331613883 | -0.483272418 | 2.878819344 |
| C | -0.440496022 | 3.546190591 | 1.413544972 |
| H | -0.818289567 | 4.432884202 | 0.909731834 |
| C | 0.932585709 | 3.228120509 | 1.596395736 |

| | | | |
|---|---|---|---|
| Zr | 2.053574546 | 0.271579083 | 0.042035706 |
| As | -0.056326160 | 0.027712440 | 1.788066011 |
| As | 0.088118922 | 0.014304330 | -1.863413326 |
| As | -1.333656723 | 1.070432508 | -0.092192061 |
| As | -0.991668745 | -1.341799364 | -0.088356969 |
| Mn | -3.261779616 | 2.479570350 | -0.063944834 |
| Mn | -2.361884478 | -3.267400368 | -0.127600301 |
| O | -2.896733470 | 3.589825668 | -2.767508798 |
| O | -2.963902446 | -3.011824976 | -3.001518439 |
| O | -5.058984328 | 0.320979313 | -0.933564596 |
| C | 1.725635775 | 2.916535997 | 0.168278272 |
| H | 0.755703636 | 3.389204101 | 0.305234080 |
| C | 2.394995961 | -2.389490284 | -0.068882444 |
| H | 1.597936128 | -3.106746494 | -0.244187918 |
| C | -4.832208550 | 3.083519333 | 1.278081645 |
| H | -5.842997274 | 2.679444899 | 1.240542631 |
| C | 2.871778968 | -2.002708923 | 1.218103261 |
| C | 2.617580226 | 2.561616657 | 1.226186693 |
| C | 3.212941488 | -1.818488961 | -1.089557678 |
| C | 3.322087958 | -2.301564749 | -2.539496011 |
| C | 2.343418679 | 2.678563067 | -1.096412127 |
| C | 3.602419246 | 2.076735603 | -0.810635975 |
| H | 4.352357574 | 1.796345920 | -1.547414365 |
| C | 3.774880947 | 2.019244252 | 0.599515664 |
| H | 4.678438209 | 1.686317381 | 1.105704760 |
| C | 3.755347994 | -1.170128571 | -3.487864987 |
| H | 3.007805208 | -0.365662553 | -3.519543061 |
| H | 3.879934702 | -1.557682748 | -4.511476532 |
| H | 4.718958534 | -0.728945751 | -3.187068732 |
| C | 2.010706496 | -2.924732683 | -3.049930522 |
| H | 1.685033205 | -3.772517303 | -2.428715104 |
| H | 2.151406561 | -3.304713661 | -4.074253239 |
| H | 1.190613191 | -2.191526309 | -3.081807471 |
| C | 3.949036350 | -1.099406635 | 0.984723394 |
| H | 4.568377168 | -0.638241172 | 1.751698124 |
| C | 4.166192277 | -1.000149858 | -0.418312852 |

| | | | |
|---|---|---|---|
| H | 1.770161018 | 3.832947122 | 1.256042503 |
| C | 4.621947755 | -3.859720364 | -0.067150362 |
| H | 4.915630783 | -4.406274031 | -0.962384505 |
| C | 3.386268776 | -3.038853926 | 1.687553476 |
| H | 2.562517761 | -2.844059521 | 2.371910154 |
| C | 4.906693101 | -0.398992191 | -0.212264826 |
| C | 3.708045125 | -2.003632581 | -1.878878547 |
| C | 2.741466817 | 2.710394121 | -2.464143690 |
| C | 5.470530576 | -3.028481909 | 0.711571587 |
| H | 6.524727169 | -2.833136519 | 0.522160901 |
| C | 3.348277199 | 1.406931892 | -3.003573769 |
| H | 3.266296186 | 0.593772281 | -2.272360583 |
| H | 4.417305292 | 1.546501359 | -3.225485471 |
| H | 2.857311860 | 1.079433649 | -3.932477483 |
| C | 4.699746145 | -2.520190577 | 1.808460068 |
| H | 5.062312043 | -1.866793578 | 2.600043858 |
| C | -2.710003017 | 2.738602539 | 2.442165281 |
| C | -2.208075697 | 1.588301075 | -3.301010124 |
| C | 2.214968056 | 2.590328121 | 4.504355743 |
| H | 2.240253849 | 3.655445817 | 4.226512747 |
| H | 3.082691652 | 2.385033739 | 5.152622787 |
| H | 1.300462572 | 2.415808396 | 5.092244615 |
| C | -3.295992973 | 1.444598009 | 3.026335196 |
| H | -3.200486381 | 0.607114665 | 2.324914531 |
| H | -4.366840780 | 1.573985862 | 3.245083831 |
| H | -2.797757665 | 1.158776205 | 3.965238609 |
| C | -4.909400265 | -0.334878178 | 0.301814369 |
| C | -3.530095628 | 1.760737504 | -2.534818777 |
| H | -3.596117962 | 1.072378330 | -1.681968269 |
| H | -4.382334524 | 1.547138446 | -3.199411236 |
| H | -3.660644089 | 2.787392813 | -2.160749672 |
| C | -2.105354895 | 0.146003835 | -3.826493647 |
| H | -1.190134656 | -0.013773784 | -4.417114651 |
| H | -2.959913873 | -0.070468485 | -4.487319824 |
| H | -2.120077140 | -0.587524036 | -3.009044123 |
| C | 3.560936517 | 3.202739517 | -1.257736256 |
| H | 3.177173049 | 4.158395332 | -0.865640172 |
| H | 4.609802449 | 3.366066164 | -1.550210728 |
| H | 3.559447000 | 2.467384202 | -0.442447869 |
| C | -3.546297532 | 3.193798549 | 1.232253032 |
| H | -3.171877223 | 4.141614042 | 0.813015013 |
| H | -4.592859728 | 3.358011145 | 1.531928109 |
| H | -3.550369080 | 2.439121649 | 0.434834544 |
| C | -2.780200115 | 3.840074415 | 3.532027268 |
| H | -2.181550159 | 3.565291307 | 4.414690654 |
| H | -3.823169149 | 3.982965671 | 3.859209327 |
| H | -2.405907162 | 4.805209938 | 3.156407985 |
| C | 2.813522967 | 3.782873212 | -3.582096966 |
| H | 2.229267659 | 3.477616369 | -4.464357934 |
| H | 3.858841437 | 3.929282666 | -3.900076897 |
| H | 2.423380436 | 4.753149480 | -3.237060932 |
| C | -2.221791120 | 2.545428982 | -4.523732023 |
| H | -2.321980508 | 3.595624223 | -4.208741986 |
| H | -3.070174848 | 2.304653165 | -5.185229742 |
| H | -1.294316755 | 2.455309097 | -5.110371445 |
| C | -4.640742522 | -2.402015968 | -1.836046332 |
| H | -4.874982313 | -1.727770063 | -2.657401978 |
| C | -4.843282119 | -3.722234368 | 0.047491722 |
| H | -5.259798696 | -4.227715059 | 0.917554334 |
| O | -3.616874246 | -2.198585988 | 3.040163788 |
| C | -3.704663196 | -1.998896011 | 1.902704488 |
| C | -5.538618860 | -2.820190473 | -0.800067232 |
| H | -6.578680653 | -2.516079098 | -0.692435892 |
| C | -3.399312699 | -3.052190325 | -1.610245432 |
| H | -2.508243522 | -2.950672031 | -2.226972612 |
| C | -3.509490894 | -3.870002823 | -0.453133515 |
| H | -2.731055349 | -4.507308156 | -0.038054580 |

| | | | |
|---|---|---|---|
| H | 4.978870284 | -0.450431911 | -0.888074779 |
| C | -3.754211321 | 2.570301668 | 2.063785006 |
| H | -3.794118657 | 1.719372417 | 2.740801223 |
| C | -4.366929327 | 4.218550662 | 0.560865198 |
| H | -4.958261095 | 4.837231051 | -0.112016036 |
| C | -3.010239603 | 3.115363389 | -1.719440712 |
| C | -4.311364012 | 1.147960002 | -0.622393467 |
| C | 1.972831142 | 3.339919628 | -2.431143376 |
| C | -2.621881506 | 3.402568546 | 1.822752463 |
| H | -1.637451588 | 3.273961076 | 2.267212128 |
| C | 0.473818594 | 3.659184470 | -2.540296508 |
| H | -0.141051015 | 2.749608441 | -2.553053844 |
| H | 0.269725788 | 4.203444498 | -3.475372535 |
| H | 0.123956944 | 4.294655161 | -1.712657750 |
| C | -2.990402711 | 4.420857468 | 0.907093309 |
| H | -2.348098400 | 5.219201138 | 0.539116722 |
| C | 2.597454827 | -2.732378801 | 2.539412375 |
| C | 2.551043929 | 3.038336513 | 2.681517469 |
| C | 4.415666647 | -3.401951933 | -2.549482221 |
| H | 5.387992313 | -3.004566035 | -2.218631132 |
| H | 4.540705273 | -3.808256929 | -3.566813390 |
| H | 4.145393135 | -4.234822921 | -1.881875384 |
| C | 1.270252589 | -3.509823620 | 2.505189836 |
| H | 0.410395315 | -2.849685538 | 2.323647278 |
| H | 1.109829501 | -4.016540549 | 3.470380182 |
| H | 1.269646411 | -4.283607983 | 1.723631140 |
| C | -2.692051599 | -3.085549278 | -1.882120626 |
| C | 3.156257138 | 2.012660562 | 3.656128942 |
| H | 2.564878157 | 1.087318703 | 3.681418773 |
| H | 3.180417207 | 2.426432169 | 4.677064788 |
| H | 4.191367192 | 1.750684258 | 3.386510012 |
| C | 1.115123374 | 3.364363637 | 3.123976770 |
| H | 0.662080337 | 4.145663714 | 2.494659632 |
| H | 1.116073453 | 3.739289957 | 4.159723281 |
| H | 0.468876760 | 2.474203302 | 3.092978361 |
| C | 2.399388280 | 2.498531236 | -3.647319438 |
| H | 3.473120812 | 2.256627657 | -3.628409553 |
| H | 2.206166971 | 3.057500524 | -4.576573922 |
| H | 1.836049798 | 1.555700942 | -3.704889080 |
| C | 2.609731390 | -1.788814363 | 3.754759565 |
| H | 3.544074861 | -1.210304697 | 3.817361487 |
| H | 2.521405018 | -2.371683687 | 4.685597766 |
| H | 1.769187269 | -1.079637774 | 3.724387764 |
| C | 3.750351089 | -3.757788782 | 2.707490208 |
| H | 3.793367845 | -4.448071933 | 1.850818675 |
| H | 3.598704479 | -4.356364774 | 3.620970517 |
| H | 4.727021565 | -3.255432056 | 2.787138676 |
| C | 2.756947468 | 4.679276161 | -2.465798464 |
| H | 2.483689275 | 5.320822046 | -1.613388718 |
| H | 2.530857007 | 5.230485568 | -3.393483092 |
| H | 3.844329169 | 4.510094750 | -2.425209765 |
| C | 3.387415334 | 4.342883053 | 2.752408202 |
| H | 4.439093906 | 4.159216414 | 2.483350416 |
| H | 3.364129958 | 4.759684168 | 3.772970475 |
| H | 2.992239814 | 5.105567097 | 2.063229407 |
| C | -3.372366316 | -4.736031654 | 1.076985294 |
| H | -3.111640754 | -5.793457711 | 1.085645952 |
| C | -4.371773363 | -2.724614791 | 0.534804742 |
| H | -4.988003423 | -1.971167128 | 0.048358226 |
| O | -0.138153426 | -5.148286201 | -0.537828297 |
| C | -0.999921788 | -4.383694954 | -0.394980860 |
| C | -4.309087508 | -4.117987278 | 0.205684804 |
| H | -4.890349705 | -4.619250538 | -0.566707701 |
| C | -2.844758929 | -3.733442305 | 1.951601170 |
| H | -2.118967694 | -3.888805152 | 2.747696455 |
| C | -3.464804035 | -2.499268174 | 1.604719318 |
| Zr | 2.053574546 | 0.271579083 | 0.042035706 |

**Table S10:** Cartesian coordinates of the optimized geometry of the Isomers for [Cp''$_2$Zr($\mu_3$,$\eta^{1:1:1}$-As$_4$)(CpMn(CO)$_2$)] at the B3LYP/def2- SVP level of theory.

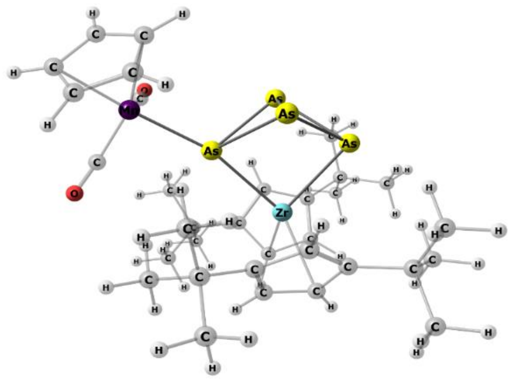

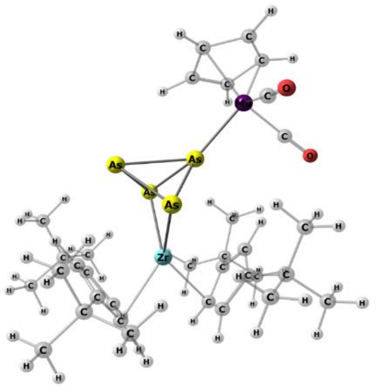

| | | | |
|---|---|---|---|
| Zr | 1.257331367 | 0.047352463 | 0.524050588 |
| As | 1.695748828 | 0.022326011 | -2.165797944 |
| As | -1.374392899 | -0.026658240 | -0.210771919 |
| As | -0.440330943 | -1.243841300 | -2.216569521 |
| As | -0.479528104 | 1.215161713 | -2.220514829 |
| Mn | -3.782290323 | -0.185902383 | -0.193627852 |
| O | -3.978772380 | -0.094136074 | 2.738323493 |
| O | -3.724166859 | -3.121330942 | -0.355803974 |
| C | 1.150757164 | -2.613378506 | 0.808130279 |
| H | 0.429604302 | -3.229710919 | 0.276789534 |
| C | 1.461789295 | 2.740956145 | 0.354476130 |
| H | 1.104916979 | 3.313734186 | -0.497631309 |
| C | -4.312692411 | 0.419501622 | -2.233630847 |
| H | -3.777296477 | 0.126052509 | -3.134492787 |
| C | 2.786690659 | 2.229489167 | 0.490799194 |
| C | 2.481314835 | -2.351041155 | 0.371444837 |
| C | 0.712511030 | 2.489140901 | 1.537127095 |
| C | -0.543547459 | 3.243644257 | 1.987901766 |
| C | 0.937688073 | -2.049711849 | 2.103536176 |
| C | 2.135503927 | -1.358819223 | 2.434586891 |
| H | 2.338663566 | -0.848629542 | 3.373328297 |
| C | 3.065694294 | -1.519014469 | 1.372296071 |
| H | 4.091804292 | -1.156381631 | 1.373872178 |
| C | -1.358524741 | 2.451723730 | 3.023396654 |
| H | -1.745933828 | 1.513647977 | 2.604711069 |
| H | -2.223279081 | 3.045136155 | 3.359546955 |
| H | -0.764001643 | 2.209256059 | 3.917348952 |
| C | -1.458624471 | 3.630566850 | 0.813583710 |
| H | -0.923030672 | 4.206314158 | 0.042955772 |
| H | -2.284373209 | 4.262251744 | 1.178244382 |
| H | -1.901343137 | 2.744177580 | 0.339818860 |
| C | 2.830942281 | 1.586208243 | 1.760605273 |
| H | 3.708418433 | 1.130959416 | 2.214224229 |
| C | 1.564544234 | 1.726944848 | 2.390673895 |
| H | 1.320346259 | 1.395873135 | 3.397882057 |
| C | -5.396573028 | -0.282292976 | -1.628977713 |
| H | -5.825409433 | -1.218719393 | -1.983750884 |
| C | -4.081583413 | 1.594469483 | -1.455580035 |
| H | -3.327881655 | 2.353341657 | -1.657758690 |
| C | -3.855749508 | -0.143820622 | 1.585628143 |
| C | -3.705081055 | -1.966566604 | -0.266380118 |
| C | -0.172135466 | -2.420694486 | 3.091698736 |
| C | -5.828947936 | 0.445864633 | -0.486515843 |
| H | -6.648944967 | 0.174206913 | 0.175815205 |
| C | -1.425316742 | -2.944253830 | 2.374328129 |
| H | -1.855134549 | -2.183332152 | 1.710780192 |
| H | -2.198461430 | -3.215422922 | 3.109792719 |
| H | -1.214199166 | -3.840992993 | 1.772456854 |
| C | -5.010551391 | 1.617392367 | -0.385984688 |
| H | -5.092240403 | 2.391726053 | 0.375141159 |
| C | 4.011575025 | 2.634652161 | -0.337423942 |
| C | 3.273086012 | -3.159332229 | -0.666486495 |
| C | -0.033225328 | 4.547754739 | 2.656913500 |
| H | 0.619576826 | 4.327566086 | 3.515914873 |
| H | -0.883130396 | 5.149275270 | 3.019502602 |
| H | 0.540901049 | 5.161656642 | 1.945454406 |
| C | 3.619378122 | 3.178517720 | -1.722952867 |
| H | 3.087903069 | 2.427793690 | -2.326452024 |
| H | 4.523081993 | 3.474883396 | -2.278805624 |

| | | | |
|---|---|---|---|
| Zr | -1.497270412 | 0.447226805 | 0.249804540 |
| As | 0.107242259 | -1.538062654 | 1.210129037 |
| As | -0.001345078 | 0.155875288 | -2.021659994 |
| As | 0.122885607 | -2.224934348 | -1.222554743 |
| As | 1.716973524 | -0.565768311 | -0.393521290 |
| Mn | 4.079102736 | -0.470603777 | -0.303627708 |
| O | 4.343947747 | 0.876986246 | -2.910019169 |
| C | -3.237404527 | -1.344063338 | -0.763032106 |
| H | -2.938401966 | -2.195372030 | -1.369223584 |
| C | 0.240819554 | 2.241376536 | 1.257453157 |
| H | 1.316777266 | 2.090971926 | 1.289030756 |
| C | -0.657403506 | 1.900799646 | 2.311035863 |
| C | -3.426500148 | -1.377949812 | 0.649286303 |
| C | -0.453915994 | 2.925943483 | 0.219254126 |
| C | 0.175027915 | 3.849384738 | -0.832541739 |
| C | -3.607908137 | -0.068695049 | -1.282022687 |
| C | -3.966226683 | 0.725725390 | -0.154607525 |
| H | -4.349544590 | 1.743411648 | -0.186887995 |
| C | -3.845889914 | -0.066552111 | 1.020755798 |
| H | -4.125367232 | 0.250311795 | 2.023780026 |
| C | -0.660732756 | 3.931151614 | -2.121381980 |
| H | -0.672559190 | 2.968859101 | -2.652937802 |
| H | -0.231819861 | 4.683508246 | -2.802222233 |
| H | -1.702781403 | 4.224997136 | -1.921511538 |
| C | 1.617628475 | 3.447175537 | -1.186800787 |
| H | 2.274248487 | 3.443490405 | -0.304392876 |
| H | 2.039319512 | 4.168884747 | -1.904235118 |
| H | 1.667727432 | 2.453290412 | -1.654653034 |
| C | -1.950111271 | 2.314510446 | 1.877746690 |
| H | -2.868868647 | 2.255102912 | 2.457185469 |
| C | -1.828643650 | 2.925878171 | 0.598052319 |
| H | -2.636588908 | 3.409623638 | 0.052626400 |
| C | -3.943464695 | 0.252157799 | -2.742282130 |
| C | -3.232965534 | -0.700809788 | -3.720038769 |
| H | -2.137573195 | -0.630862340 | -3.640823633 |
| H | -3.509331914 | -0.450707089 | -4.756630503 |
| H | -3.519967757 | -1.750021199 | -3.549973780 |
| C | -0.270408399 | 1.548552207 | 3.751083799 |
| C | -3.598772526 | -2.640815145 | 1.505794786 |
| C | 0.214574512 | 5.259774063 | -0.185348057 |
| H | -0.797610632 | 5.619542338 | 0.057112851 |
| H | 0.678751791 | 5.983423294 | -0.875673188 |
| H | 0.802613135 | 5.251878690 | 0.745527732 |
| C | 1.114009350 | 0.880720057 | 3.835850654 |
| H | 1.136886143 | -0.083842848 | 3.306192965 |
| H | 1.371822347 | 0.687785794 | 4.889593590 |
| H | 1.906883486 | 1.517547918 | 3.415126330 |
| C | 4.202709508 | 0.363423164 | -1.884491197 |
| C | -3.147391726 | -2.440621094 | 2.963496419 |
| H | -2.057895279 | -2.308854959 | 3.036021733 |
| H | -3.415685035 | -3.323936014 | 3.564954597 |
| H | -3.630177720 | -1.567893794 | 3.429091857 |
| C | -2.866962921 | -3.857978905 | 0.912265244 |
| H | -3.214370447 | -4.087831504 | -0.106807609 |
| H | -3.061278458 | -4.748105950 | 1.531594399 |
| H | -1.778498444 | -3.707173787 | 0.876643614 |
| C | -3.600435593 | 1.706562507 | -3.109323974 |
| H | -4.094130714 | 2.426403144 | -2.437613658 |
| H | -3.937584658 | 1.930949324 | -4.133932627 |

**Table S11:** Cartesian coordinates of the optimized geometry of the Isomers for [$Cp''_2Zr(\mu,\eta^{1:1:1:1}$-$As_4)(Fe(CO)_4)_2$] at the B3LYP/def2- SVP level of theory.

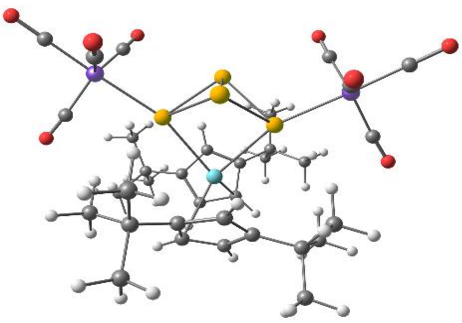

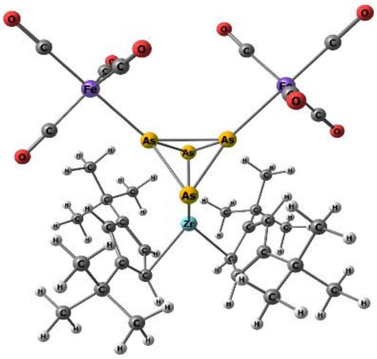


| | | | |
|---|---|---|---|
| As | -1.774259940 | -0.609960569 | -0.040873014 |
| As | -0.024696323 | -1.855646597 | 1.227965765 |
| Fe | -3.890859910 | -1.933966386 | 0.011304099 |
| C | 0.471323292 | 1.595876840 | -2.643333314 |
| H | 0.779736067 | 0.734744856 | -3.211471518 |
| C | -1.932975993 | 0.194830088 | -3.910310910 |
| H | -1.975558135 | -0.543945094 | -3.112820433 |
| H | -1.008550304 | 0.042823875 | -4.469744244 |
| H | -2.760991864 | -0.010020174 | -4.590957865 |
| C | 1.360267978 | 2.530218507 | -2.043772739 |
| C | -0.815553376 | 3.196306743 | -1.682623110 |
| H | -1.658527839 | 3.808656844 | -1.406833126 |
| C | 0.543454880 | 3.510999642 | -1.431445493 |
| H | 0.892448540 | 4.394338613 | -0.923660991 |
| C | -0.868391031 | 2.030487893 | -2.494541240 |
| C | -2.010498651 | 2.595090759 | -4.586102198 |
| H | -2.828066562 | 2.361292810 | -5.271380449 |
| H | -1.071385948 | 2.504052137 | -5.134361034 |
| H | -2.119112027 | 3.633376951 | -4.268569485 |
| C | -3.434952421 | -2.521458671 | 1.657841501 |
| C | 3.474301126 | 1.374189235 | -2.840793751 |
| H | 3.395777514 | 0.566121864 | -2.115180409 |
| H | 4.535100174 | 1.527632862 | -3.044936565 |
| H | 3.007512710 | 1.043948468 | -3.770061016 |
| C | 2.971193424 | 3.740028868 | -3.451588828 |
| H | 4.020534869 | 3.875179065 | -3.722506915 |
| H | 2.576109658 | 4.705373003 | -3.131160716 |
| H | 2.427349313 | 3.431485427 | -4.346022450 |
| C | -3.363758391 | -2.777205035 | -1.498042836 |
| C | -2.048938197 | 1.627903933 | -3.375300397 |
| C | 2.850936074 | 2.678791338 | -2.329522924 |
| C | -3.394704052 | 1.802844651 | -2.663232395 |
| H | -3.541892507 | 2.825441234 | -2.312768241 |
| H | -3.483133317 | 1.132745532 | -1.811203942 |
| H | -4.212535181 | 1.572772242 | -3.348080477 |
| C | 3.627679855 | 3.182618859 | -1.104316580 |
| H | 3.599185962 | 2.465788865 | -0.285813536 |
| H | 3.236237882 | 4.136067730 | -0.743410642 |
| H | 4.675707649 | 3.339054079 | -1.364909281 |
| C | -4.895663856 | -0.441505298 | -0.095981020 |
| Zr | -0.000011414 | 1.522229338 | 0.000137209 |
| As | 1.774374613 | -0.609845547 | 0.040740972 |
| As | 0.024908441 | -1.855065333 | -1.228740592 |
| Fe | 3.891056547 | -1.933668887 | -0.011592828 |
| C | -0.471590460 | 1.595157514 | 2.643487178 |
| H | -0.780055055 | 0.733834989 | 3.211310098 |
| C | 1.932255370 | 0.193944456 | 3.910771278 |
| H | 1.974565377 | -0.544817411 | 3.113254734 |
| H | 1.007833136 | 0.042168505 | 4.470274786 |
| H | 2.760272543 | -0.011156142 | 4.591342222 |
| C | -1.360474850 | 2.529712323 | 2.044141406 |
| C | 0.815388225 | 3.195869331 | 1.683385432 |
| H | 1.658391671 | 3.808304301 | 1.407862757 |
| C | -0.543592794 | 3.510670970 | 1.432202561 |
| H | -0.892520530 | 4.394178135 | 0.924664243 |
| C | 0.868133486 | 2.029817271 | 2.494977255 |
| C | 2.010153881 | 2.594169297 | 4.586635820 |
| H | 2.827509772 | 2.360098182 | 5.272072824 |
| H | 1.070899454 | 2.503398249 | 5.134696130 |
| H | 2.119150519 | 3.632431120 | 4.269156417 |
| C | 3.435107380 | -2.521314312 | -1.658062401 |
| C | -3.474577902 | 1.373424658 | 2.840572217 |
| H | -3.395987555 | 0.565645086 | 2.114642739 |
| H | -4.535397122 | 1.526799609 | 3.044663568 |
| H | -3.007894766 | 1.042797142 | 3.769754649 |
| C | -2.971408370 | 3.738988723 | 3.452414773 |

| | | | |
|---|---|---|---|
| As | -0.048907414 | -0.014029116 | -1.830332377 |
| As | -1.215999015 | 1.207001943 | -0.015669481 |
| Fe | -2.993824721 | 2.862855542 | -0.064521718 |
| C | 2.038481506 | -2.674450439 | 0.232938404 |
| H | 1.164538808 | -3.273804169 | 0.425772502 |
| C | 0.782272678 | -3.656293167 | -2.380492842 |
| H | 0.056656473 | -2.843631819 | -2.384366907 |
| H | 0.562618759 | -4.306329970 | -1.533077805 |
| H | 0.620807100 | -4.240914804 | -3.288017547 |
| C | 2.907975698 | -2.172699315 | 1.239356120 |
| C | 3.742920150 | -1.634689563 | -0.841218870 |
| H | 4.419492027 | -1.291845005 | -1.607047989 |
| C | 3.955342539 | -1.506908196 | 0.555088820 |
| H | 4.815545209 | -1.045342072 | 1.010031767 |
| C | 2.569422969 | -2.403052314 | -1.056389213 |
| C | 3.171376367 | -4.371779570 | -2.393327888 |
| H | 2.974811483 | -4.960931160 | -3.291756317 |
| H | 3.017524755 | -5.014927374 | -1.525429759 |
| H | 4.219207350 | -4.067556263 | -2.407775719 |
| C | -3.182829138 | 2.673488919 | 1.730190144 |
| C | 1.546036350 | -2.975650911 | 3.239211609 |
| H | 0.862508829 | -2.125372712 | 3.254367970 |
| H | 1.634052899 | -3.337867743 | 4.265371228 |
| H | 1.086272821 | -3.770521236 | 2.651947145 |
| C | 3.842621128 | -3.841709604 | 2.785839969 |
| H | 3.907820739 | -4.193677331 | 3.817778352 |
| H | 4.852712809 | -3.619345647 | 2.437661967 |
| H | 3.445454034 | -4.654491341 | 2.175670375 |
| C | -3.913497383 | 1.785508624 | -1.200386926 |
| C | 2.228055393 | -3.143210992 | -2.350013693 |
| C | 2.933171276 | -2.590585852 | 2.707657994 |
| C | 2.490712288 | -2.294585758 | -3.601924613 |
| H | 3.518709496 | -1.931071204 | -3.640801075 |
| H | 1.822058981 | -1.435016545 | -3.653044500 |
| H | 2.323923652 | -2.895652726 | -4.497824791 |
| C | 3.531886723 | -1.496864025 | 3.603334516 |
| H | 2.919918016 | -0.595921719 | 3.591904091 |
| H | 4.544259375 | -1.227959341 | 3.296308580 |
| H | 3.588971899 | -1.849739254 | 4.634863394 |
| C | -1.827903855 | 4.067178414 | -0.727768326 |
| Zr | 2.032495755 | 0.000747254 | 0.000328644 |
| As | -0.049964078 | 0.014080868 | 1.829714335 |
| As | -1.214805302 | -1.207962892 | 0.014367229 |
| Fe | -2.991124578 | -2.865461328 | 0.062615383 |
| C | 2.036536743 | 2.675809006 | -0.232156911 |
| H | 1.162272421 | 3.274558691 | -0.425400854 |
| C | 0.778295133 | 3.657072074 | 2.380465161 |
| H | 0.053075321 | 2.844050779 | 2.383984645 |
| H | 0.558785711 | 4.306936286 | 1.532874278 |
| H | 0.616032598 | 4.241688805 | 3.287865382 |
| C | 2.907062269 | 2.174959912 | -1.238088767 |
| C | 3.741162726 | 1.637332994 | 0.842947803 |
| H | 4.417529457 | 1.294894977 | 1.609136826 |
| C | 3.954503650 | 1.509843939 | -0.553279976 |
| H | 4.815377518 | 1.049045058 | -1.007735387 |
| C | 2.566960914 | 2.404740331 | 1.057473259 |
| C | 3.166950609 | 4.373872937 | 2.394689356 |
| H | 2.969474286 | 4.962968529 | 3.292957819 |
| H | 3.013263359 | 5.016890786 | 1.526665251 |
| H | 4.214950405 | 4.070254617 | 2.409808669 |
| C | -3.175234953 | -2.686518168 | -1.733674236 |
| C | 1.545678743 | 2.976900182 | -3.238722961 |
| H | 0.862803406 | 2.126120233 | -3.254403129 |
| H | 1.634043040 | 3.339308259 | -4.264779592 |
| H | 1.084979363 | 3.771353002 | -2.651615028 |
| C | 3.841335926 | 3.844632594 | -2.784040581 |

| | | | |
|---|---|---|---|
| H | -4.020754658 | 3.874047150 | 3.723356599 |
| H | -2.576290562 | 4.704459066 | 3.132411728 |
| H | -2.427600296 | 3.430046139 | 4.346732426 |
| C | 3.364101652 | -2.776835029 | 1.497847332 |
| C | 2.048550040 | 1.627006221 | 3.375809625 |
| C | -2.851147903 | 2.678224679 | 2.329893014 |
| C | 3.394430880 | 1.801714492 | 2.663899973 |
| H | 3.541722679 | 2.824207220 | 2.313184126 |
| H | 3.482982956 | 1.131373179 | 1.812074380 |
| H | 4.212143749 | 1.571790094 | 3.348940565 |
| C | -3.627822643 | 3.182592617 | 1.104868877 |
| H | -3.599308631 | 2.466107387 | 0.286067180 |
| H | -3.236328715 | 4.136178757 | 0.744379925 |
| H | -4.675855576 | 3.338958308 | 1.365481708 |
| C | 4.895744534 | -0.441120916 | 0.095603043 |
| C | 5.364364972 | -2.931299217 | -0.052577585 |
| C | -5.364096256 | -2.931698915 | 0.052258207 |
| O | 5.599359253 | 0.464962358 | 0.147933458 |
| O | 6.309090615 | -3.573578477 | -0.079019688 |
| O | 3.064840546 | -3.324679757 | 2.460130351 |
| O | 3.188598557 | -2.898213531 | -2.713011786 |
| O | -3.064390856 | -3.325091494 | -2.460269059 |
| O | -3.188478656 | -2.898299385 | 2.712819960 |
| O | -6.308742575 | -3.574095726 | 0.078674847 |
| O | -5.599314961 | 0.464546502 | -0.148370061 |

| | | | |
|---|---|---|---|
| H | 3.906913804 | 4.196608719 | -3.815953627 |
| H | 4.851392146 | 3.623085871 | -2.435211924 |
| H | 3.443135613 | 4.657135311 | -2.174161145 |
| C | -3.911511513 | -1.782181036 | 1.192109636 |
| C | 2.224369959 | 3.144745691 | 2.350852805 |
| C | 2.932781950 | 2.592800326 | -2.706380439 |
| C | 2.486799207 | 2.296304949 | 3.602947317 |
| H | 3.515015479 | 1.933460212 | 3.642431099 |
| H | 1.818688509 | 1.436286306 | 3.653662622 |
| H | 2.319078849 | 2.897259477 | 4.498750984 |
| C | 3.532879550 | 1.499578541 | -3.601763648 |
| H | 2.921623592 | 0.598140660 | -3.590846457 |
| H | 4.545274623 | 1.231436306 | -3.294123433 |
| H | 3.590338214 | 1.852615793 | -4.633214446 |
| C | -1.826392636 | -4.068542422 | 0.730290623 |
| C | -4.305726909 | -4.068529928 | 0.098826245 |
| C | -4.305056934 | 4.069764445 | -0.093141216 |
| O | -1.094590587 | -4.849530279 | 1.147764932 |
| O | -5.147868436 | -4.839174386 | 0.122956108 |
| O | -4.510903899 | -1.124899722 | 1.910607846 |
| O | -3.299881384 | -2.609310313 | -2.868324674 |
| O | -4.512581417 | 1.132078393 | -1.922579904 |
| O | -3.310516603 | 2.589493059 | 2.864019240 |
| O | -5.144597317 | 4.843411069 | -0.111287815 |
| O | -1.095109705 | 4.848930768 | -1.142172084 |

**Table S12:** Cartesian coordinates of the optimized geometry of the Isomers for [Cp''$_2$Zr(μ,η$^{1:1:1}$-As$_4$)(Fe(CO)$_4$)] at the B3LYP/def2- SVP level of theory.

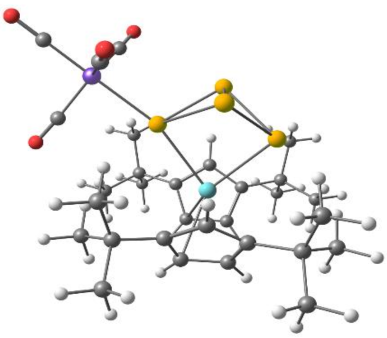

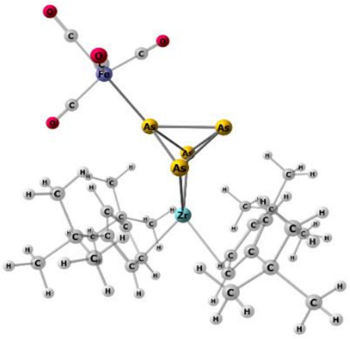

| | | | |
|---|---|---|---|
| As | -0.454739521 | -1.224162621 | -2.249529099 |
| C | 1.235554247 | -2.697986164 | 0.600140177 |
| H | 0.712568482 | -3.286346972 | -0.134605336 |
| C | 0.700605917 | -2.311646739 | 1.855289413 |
| C | 2.871514390 | -1.560594350 | 1.680347240 |
| H | 3.830510849 | -1.155457830 | 1.955835980 |
| O | -3.840859169 | 2.568378865 | -1.813539323 |
| C | 2.592066178 | -2.295969652 | 0.498203914 |
| C | 1.720738837 | -1.571331255 | 2.506925659 |
| H | 1.667746006 | -1.167036445 | 3.503737177 |
| C | -0.525648389 | -2.914286533 | 2.533589139 |
| C | -1.578627731 | -3.391489475 | 1.524729997 |
| H | -1.984029570 | -2.568741537 | 0.937878582 |
| H | -2.411521897 | -3.857089940 | 2.054731651 |
| H | -1.174577523 | -4.135425801 | 0.836375564 |
| C | 3.641551072 | -2.899690946 | -0.434703553 |
| C | 4.292789055 | -4.063696645 | 0.353646380 |
| H | 4.799643115 | -3.702960880 | 1.250096588 |
| H | 5.029782300 | -4.572233219 | -0.272251451 |
| H | 3.544599746 | -4.796505708 | 0.660458811 |
| C | -1.171999112 | -1.941839061 | 3.527113269 |
| H | -0.461310794 | -1.600491658 | 4.281751994 |
| H | -1.994845683 | -2.431688355 | 4.050706725 |
| H | -1.578594271 | -1.069554995 | 3.021230064 |
| C | -0.016729054 | -4.148335948 | 3.319756318 |
| H | 0.454189808 | -4.873822324 | 2.654136473 |
| H | -0.853073689 | -4.640411104 | 3.821580263 |
| H | 0.713822723 | -3.861652305 | 4.078134712 |
| C | 3.022877621 | -3.483291720 | -1.713495263 |
| H | 2.298675351 | -4.267848539 | -1.488517498 |
| H | 3.807059057 | -3.930376867 | -2.327670608 |
| H | 2.525501210 | -2.721259802 | -2.312547923 |
| C | -3.839715527 | 1.567172234 | -1.252802546 |
| C | 4.742946472 | -1.900404742 | -0.812992635 |
| H | 4.353827745 | -1.096422869 | -1.435611124 |
| H | 5.528685462 | -2.408256455 | -1.376152630 |
| H | 5.207871056 | -1.454766324 | 0.068005633 |
| Zr | 1.203348006 | -0.000002706 | 0.599525279 |

| | | | |
|---|---|---|---|
| As | -1.777766655 | -0.648371403 | -0.120594802 |
| C | -0.244682256 | 2.525503125 | 0.647774803 |
| H | -1.301715257 | 2.386528421 | 0.798348252 |
| C | 0.360324702 | 2.877417542 | -0.585873284 |
| C | 1.970327954 | 2.772865162 | 1.062012632 |
| H | 2.919721427 | 2.876071279 | 1.560510549 |
| O | -4.101033418 | -3.155955269 | 1.508706763 |
| C | 0.725017271 | 2.507114692 | 1.685677188 |
| C | 1.751037928 | 2.986298391 | -0.322195696 |
| H | 2.505408720 | 3.282983532 | -1.032576470 |
| C | -0.353401622 | 3.452359479 | -1.810121050 |
| C | -1.800935019 | 2.959053547 | -1.937598981 |
| H | -1.852519181 | 1.884614829 | -2.109273003 |
| H | -2.281894310 | 3.450279146 | -2.785717512 |
| H | -2.390304223 | 3.192398933 | -1.050239443 |
| C | 0.446615092 | 2.590172639 | 3.184714348 |
| C | 0.427294790 | 4.097676656 | 3.538721063 |
| H | 1.389494448 | 4.566624760 | 3.325986877 |
| H | 0.210595784 | 4.230430898 | 4.601267751 |
| H | -0.338833382 | 4.624965531 | 2.968087741 |
| C | 0.394325797 | 3.168455779 | -3.120369279 |
| H | 1.432064424 | 3.502824039 | -3.078976632 |
| H | -0.087471529 | 3.699406042 | -3.943887529 |
| H | 0.385875564 | 2.105929925 | -3.362826171 |
| C | -0.387927275 | 4.986540693 | -1.596483976 |
| H | -0.913077965 | 5.242855292 | -0.674900424 |
| H | -0.907196964 | 5.468071489 | -2.428270801 |
| H | 0.619537307 | 5.402022707 | -1.537536774 |
| C | -0.917446413 | 1.994722713 | 3.560572014 |
| H | -1.737132320 | 2.494284419 | 3.043281473 |
| H | -1.087444551 | 2.113905097 | 4.632586556 |
| H | -0.974913443 | 0.929208827 | 3.335047852 |
| C | -4.130260470 | -2.187970121 | 0.897803105 |
| C | 1.543367450 | 1.912761465 | 4.018881979 |
| H | 1.570202118 | 0.837932864 | 3.846254755 |
| H | 1.355274334 | 2.074954048 | 5.082251204 |
| H | 2.532027960 | 2.320331713 | 3.798592777 |
| Zr | 1.444254255 | 0.515484194 | 0.080728542 |

| | | | |
|---|---|---|---|
| As | -1.412301252 | 0.000009611 | -0.280892632 |
| As | 1.690874386 | -0.000014266 | -2.082528310 |
| As | -0.454715334 | 1.224179703 | -2.249519669 |
| Fe | -3.896478751 | 0.000012363 | -0.358875796 |
| O | -6.816773954 | -0.000013597 | -0.475875099 |
| C | 1.235650980 | 2.697979147 | 0.600088731 |
| H | 0.712705000 | 3.286344262 | -0.134681395 |
| C | 0.700660492 | 2.311692339 | 1.855235511 |
| C | 2.871542818 | 1.560550703 | 1.680360290 |
| H | 3.830517199 | 1.155384168 | 1.955880849 |
| O | -3.840828757 | -2.568456649 | -1.813362107 |
| C | 2.592149211 | 2.295906616 | 0.498192009 |
| C | 1.720745960 | 1.571343643 | 2.506908636 |
| H | 1.667712160 | 1.167068804 | 3.503726032 |
| C | -0.525581495 | 2.914404116 | 2.533493696 |
| C | -1.578495897 | 3.391684968 | 1.524603809 |
| H | -1.983935482 | 2.568968887 | 0.937735095 |
| H | -2.411374960 | 3.857340897 | 2.054580523 |
| H | -1.174373382 | 4.135596556 | 0.836265241 |
| C | -5.674217037 | 0.000012680 | -0.429151273 |
| C | 3.641676588 | 2.899564319 | -0.434708193 |
| C | 4.292934758 | 4.063574268 | 0.353619746 |
| H | 4.799768657 | 3.702849811 | 1.250085935 |
| H | 5.029949012 | 4.572075455 | -0.272282094 |
| H | 3.544760452 | 4.796410599 | 0.660403708 |
| C | -1.172026428 | 1.941992332 | 3.526990686 |
| H | -0.461387345 | 1.600601901 | 4.281656234 |
| H | -1.994864553 | 2.431888400 | 4.050553549 |
| H | -1.578653698 | 1.069733470 | 3.021089843 |
| C | -0.016611333 | 4.148416393 | 3.319686223 |
| H | 0.454378490 | 4.873876713 | 2.654088180 |
| H | -0.852942262 | 4.640541974 | 3.821483572 |
| H | 0.713894537 | 3.861679140 | 4.078088642 |
| C | 3.023048063 | 3.483141238 | -1.713532433 |
| H | 2.298866802 | 4.267729284 | -1.488596213 |
| H | 3.807256784 | 3.930179293 | -2.327707195 |
| H | 2.525659561 | 2.721104774 | -2.312569805 |
| C | -3.839721253 | -1.567188258 | -1.252735081 |
| C | 4.743047312 | 1.900230071 | -0.812943209 |
| H | 4.353919859 | 1.096251368 | -1.435560582 |
| H | 5.528823207 | 2.408042831 | -1.376087171 |
| H | 5.207926773 | 1.454589785 | 0.068077874 |
| O | -4.064966808 | 0.000042944 | 2.585207617 |
| C | -3.958407100 | 0.000062374 | 1.441189477 |

| | | | |
|---|---|---|---|
| As | -0.134725718 | -0.442287564 | -1.948589527 |
| As | -0.119018638 | -1.113754572 | 1.637473406 |
| As | -0.221642236 | -2.470867680 | -0.477408010 |
| Fe | -4.212373407 | -0.658688205 | -0.069408121 |
| O | -7.135372790 | -0.676835461 | 0.005300251 |
| C | 3.164885193 | -1.498136411 | -0.477586484 |
| H | 2.853185098 | -2.471991071 | -0.815460484 |
| C | 3.498869206 | -0.415856265 | -1.333523725 |
| C | 3.826368274 | 0.205493349 | 0.861790556 |
| H | 4.140142384 | 0.776075483 | 1.720636367 |
| O | -4.148443995 | 1.959977569 | 1.293787906 |
| C | 3.408842863 | -1.151467103 | 0.876360171 |
| C | 3.891904686 | 0.648822627 | -0.482432229 |
| H | 4.255846450 | 1.611795625 | -0.799819815 |
| C | 3.771357659 | -0.501857994 | -2.833518152 |
| C | 3.014524923 | -1.660202346 | -3.499354139 |
| H | 1.932872799 | -1.541222626 | -3.424957522 |
| H | 3.268228752 | -1.703905767 | -4.560382549 |
| H | 3.281067143 | -2.623282365 | -3.061146455 |
| C | -5.993083743 | -0.669325404 | -0.024154330 |
| C | 3.620464592 | -2.129019686 | 2.033029033 |
| C | 5.139434388 | -2.430935351 | 2.064498813 |
| H | 5.720856074 | -1.525905177 | 2.247579853 |
| H | 5.362917461 | -3.146004630 | 2.859721723 |
| H | 5.473490740 | -2.860058745 | 1.118202249 |
| C | 3.433302515 | 0.806975244 | -3.560960431 |
| H | 3.954461856 | 1.660095257 | -3.122803859 |
| H | 3.737759724 | 0.741933380 | -4.607594108 |
| H | 2.364096074 | 1.011501811 | -3.536149908 |
| C | 5.288864926 | -0.768718544 | -2.988046502 |
| H | 5.578761928 | -1.690787960 | -2.481181271 |
| H | 5.547700970 | -0.866208551 | -4.045018128 |
| H | 5.879728737 | 0.046539863 | -2.567374873 |
| C | 2.879656576 | -3.458207666 | 1.827171659 |
| H | 3.181314734 | -3.950209456 | 0.901156940 |
| H | 3.114535121 | -4.138668139 | 2.647991995 |
| H | 1.798577895 | -3.326596781 | 1.806984193 |
| C | -4.164375966 | 0.940180619 | 0.765173427 |
| C | 3.220497584 | -1.535275028 | 3.390842338 |
| H | 2.145067859 | -1.367084691 | 3.451346491 |
| H | 3.493892522 | -2.224027878 | 4.192854735 |
| H | 3.725274057 | -0.588369413 | 3.587189609 |
| O | -4.256447151 | -0.799688394 | -3.021751928 |
| C | -4.222088783 | -0.748679078 | -1.878094180 |

**Table S13:** Cartesian coordinates of the optimized geometry of [Cp''$_2$Zr(μ,η$^{3:1:1}$-As$_4$)(Fe(CO)$_3$)] at the B3LYP/def2- SVP level of theory.

| | | | |
|---|---|---|---|
| As | 0.739518263 | 1.573521557 | 1.576914515 |
| C | -0.139333919 | -3.234947504 | 1.537058652 |
| H | 0.213554753 | -4.175642706 | 1.121910813 |
| C | -0.255722635 | -1.299693768 | 2.729000469 |
| H | 0.019274906 | -0.458469045 | 3.357377259 |
| C | 0.645124726 | -2.312074749 | 2.290250489 |
| C | 2.030855512 | -2.589959782 | 2.884244559 |
| C | -1.498152027 | -2.805676734 | 1.557310297 |
| H | -2.338922990 | -3.362273794 | 1.150136693 |
| C | -1.583828046 | -1.607084846 | 2.323028204 |
| C | 1.802102760 | -3.559084518 | 4.074176445 |
| H | 1.124191514 | -3.117977286 | 4.821616883 |
| H | 2.758954595 | -3.785485501 | 4.572932373 |
| H | 1.360124614 | -4.510093459 | 3.738007258 |
| C | 2.699320669 | -1.312303257 | 3.422653855 |
| H | 2.879895732 | -0.576903740 | 2.624441776 |
| H | 3.674070850 | -1.561210804 | 3.871525874 |
| H | 2.093805914 | -0.828517526 | 4.203995869 |
| C | 2.974115784 | -3.265494531 | 1.875856855 |
| H | 2.541764366 | -4.187807194 | 1.457117660 |
| H | 3.920483522 | -3.541320636 | 2.367765913 |
| H | 3.210260015 | -2.589092381 | 1.043685340 |
| C | -2.866143585 | -1.005725021 | 2.908535165 |
| O | -0.800126240 | 5.022200058 | 2.205778978 |
| C | -2.629689468 | 0.400158578 | 3.488181795 |
| H | -1.889605811 | 0.389494272 | 4.302672276 |
| H | -3.569141230 | 0.798163823 | 3.903248780 |
| H | -2.276611189 | 1.103486285 | 2.719562106 |
| C | -4.006972390 | -0.940828798 | 1.878304744 |
| H | -3.781301000 | -0.222664731 | 1.077630298 |
| H | -4.939580376 | -0.614933952 | 2.366068481 |
| H | -4.204023762 | -1.920822078 | 1.416287026 |
| C | -0.384266654 | 4.379476199 | 1.350679503 |
| C | -3.297444057 | -1.945736854 | 4.064660912 |
| H | -3.525269002 | -2.957716449 | 3.695146721 |
| H | -4.200087054 | -1.552035770 | 4.560346697 |
| H | -2.502837384 | -2.033637603 | 4.821931997 |

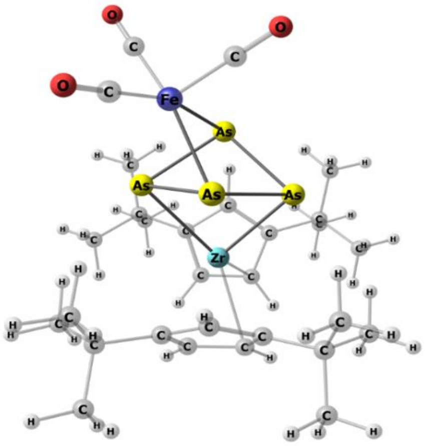

| | | | |
|---|---|---|---|
| Zr | -0.273288057 | -1.246370651 | 0.000000000 |
| As | 2.084475185 | 0.162055181 | 0.000000000 |
| As | -1.220100144 | 1.407541044 | 0.000000000 |
| As | 0.739518263 | 1.573521557 | -1.576914515 |
| Fe | 0.280852980 | 3.389215245 | 0.000000000 |
| C | -0.139333919 | -3.234947504 | -1.537058652 |
| H | 0.213554753 | -4.175642706 | -1.121910813 |
| C | -0.255722635 | -1.299693768 | -2.729000469 |
| H | 0.019274906 | -0.458469045 | -3.357377259 |
| C | 0.645124726 | -2.312074749 | -2.290250489 |
| C | 2.030855512 | -2.589959782 | -2.884244559 |
| O | 3.044256890 | 4.323417183 | 0.000000000 |
| C | -1.498152027 | -2.805676734 | -1.557310297 |
| H | -2.338922990 | -3.362273794 | -1.150136693 |
| C | -1.583828046 | -1.607084846 | -2.323028204 |
| C | 1.802102760 | -3.559084518 | -4.074176445 |
| H | 1.124191514 | -3.117977286 | -4.821616883 |
| H | 2.758954595 | -3.785485501 | -4.572932373 |
| H | 1.360124614 | -4.510093459 | -3.738007258 |
| C | 2.699320669 | -1.312303257 | -3.422653855 |
| H | 2.879895732 | -0.576903740 | -2.624441776 |
| H | 3.674070850 | -1.561210804 | -3.871525874 |
| H | 2.093805914 | -0.828517526 | -4.203995869 |
| C | 2.974115784 | -3.265494531 | -1.875856855 |
| H | 2.541764366 | -4.187807194 | -1.457117660 |
| H | 3.920483522 | -3.541320636 | -2.367765913 |
| H | 3.210260015 | -2.589092381 | -1.043685340 |
| C | -2.866143585 | -1.005725021 | -2.908535165 |
| O | -0.800126240 | 5.022200058 | -2.205778978 |
| C | -2.629689468 | 0.400158578 | -3.488181795 |
| H | -1.889605811 | 0.389494272 | -4.302672276 |
| H | -3.569141230 | 0.798163823 | -3.903248780 |
| H | -2.276611189 | 1.103486285 | -2.719562106 |
| C | -4.006972390 | -0.940828798 | -1.878304744 |
| H | -3.781301000 | -0.222664731 | -1.077630298 |
| H | -4.939580376 | -0.614933952 | -2.366068481 |
| H | -4.204023762 | -1.920822078 | -1.416287026 |
| C | -0.384266654 | 4.379476199 | -1.350679503 |
| C | 1.954520985 | 3.957036753 | 0.000000000 |
| C | -3.297444057 | -1.945736854 | -4.064660912 |
| H | -3.525269002 | -2.957716449 | -3.695146721 |
| H | -4.200087054 | -1.552035770 | -4.560346697 |
| H | -2.502837384 | -2.033637603 | -4.821931997 |

**Table S14:** Cartesian coordinates of the optimized geometry of the Isomers for [Cp''$_2$Zr(μ,η$^{1:1:1:1}$-As$_4$)(AlMe$_3$)$_2$] at the B3LYP/def2- SVP level of theory.

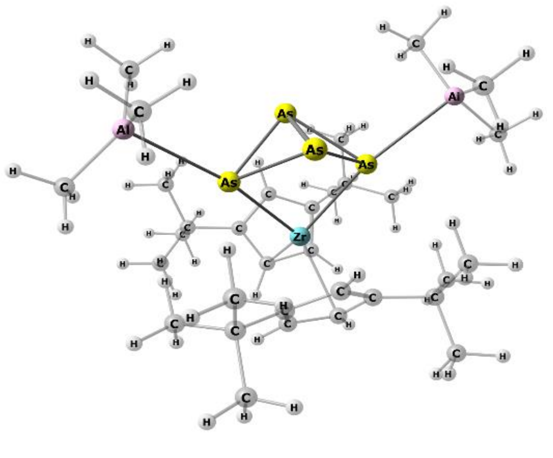

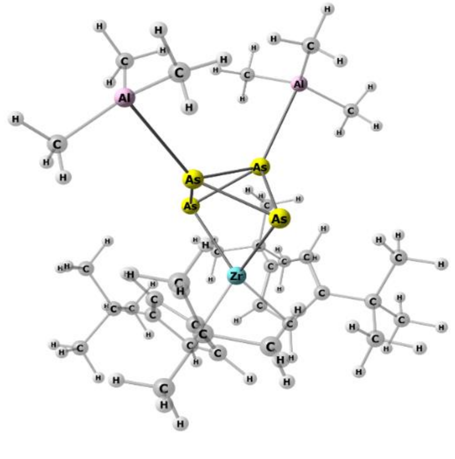

| | | | |
|---|---|---|---|
| As | -1.776120110 | -0.880677273 | 0.008344645 |
| As | 0.004411473 | -2.133812973 | 1.229491389 |
| Al | -3.997326414 | -2.638538042 | 0.074418289 |
| C | 0.178331886 | 1.194633430 | -2.672594461 |
| H | 0.329272191 | 0.289297483 | -3.255319801 |
| C | -2.448426496 | 0.018311495 | -3.646565297 |
| H | -2.388438370 | -0.735922059 | -2.849385076 |
| H | -1.628917784 | -0.167630109 | -4.357828659 |
| H | -3.392833521 | -0.146953423 | -4.188141290 |
| C | 1.225678307 | 2.037567321 | -2.196425379 |
| C | -0.823472387 | 2.942548631 | -1.611749920 |
| H | -1.567590263 | 3.655235734 | -1.261744573 |
| C | 0.586580603 | 3.112964306 | -1.514407516 |
| H | 1.083435722 | 3.972939545 | -1.070868048 |

| | | | |
|---|---|---|---|
| As | -0.561874598 | -0.056441055 | 1.828948356 |
| As | -1.741124750 | 1.214006620 | 0.042079552 |
| Al | -4.138158620 | 2.963583934 | 0.148611304 |
| C | 1.496845044 | -2.673950901 | -0.324499744 |
| H | 0.612724986 | -3.260251675 | -0.563909459 |
| C | 1.993726775 | -2.447216093 | 0.991348170 |
| C | 2.390454657 | -2.126140570 | -1.292201851 |
| C | 3.176822273 | -1.665067522 | 0.834872463 |
| H | 3.836594249 | -1.345969789 | 1.639827451 |
| C | 2.447175029 | -2.479069695 | -2.782164071 |
| C | 3.426675703 | -1.485711699 | -0.554051749 |
| H | 4.306166264 | -1.002261636 | -0.973934375 |
| C | 1.623499861 | -3.244575270 | 2.249946355 |
| C | 0.171738634 | -3.755491210 | 2.229007399 |

| | | | |
|---|---|---|---|
| C | -1.090803626 | 1.767663153 | -2.376174158 |
| C | -2.485208400 | 2.437714742 | -4.287308472 |
| H | -3.399345936 | 2.252838589 | -4.874668452 |
| H | -1.619217478 | 2.316218355 | -4.956628927 |
| H | -2.504845517 | 3.484593989 | -3.946580089 |
| C | -3.628031636 | -3.541355647 | 1.805554229 |
| H | -4.420017188 | -4.291998675 | 1.993215081 |
| H | -2.671383095 | -4.092578790 | 1.821326240 |
| H | -3.634224544 | -2.859236716 | 2.673524241 |
| C | 3.110754208 | 0.624883130 | -3.166391925 |
| H | 3.036221190 | -0.143859071 | -2.383167294 |
| H | 4.158992048 | 0.651884404 | -3.502288443 |
| H | 2.502256120 | 0.297396984 | -4.023348129 |
| C | 2.777436603 | 3.012484479 | -3.843406660 |
| H | 3.800690218 | 3.024816844 | -4.252852757 |
| H | 2.524810956 | 4.036347606 | -3.526725510 |
| H | 2.089179920 | 2.730075064 | -4.655274359 |
| C | -3.646076273 | -3.671941214 | -1.584739199 |
| H | -3.685511192 | -3.063273853 | -2.504786429 |
| H | -2.677829432 | -4.202722976 | -1.576633289 |
| H | -4.424419413 | -4.451838542 | -1.693039467 |
| C | -2.411152668 | 1.452404135 | -3.089888669 |
| C | 2.686190099 | 2.014083350 | -2.658946361 |
| C | -3.639737608 | 1.684069674 | -2.194498644 |
| H | -3.658030901 | 2.700824242 | -1.772804401 |
| H | -3.676797960 | 0.965832677 | -1.363313758 |
| H | -4.563212022 | 1.556292721 | -2.780781458 |
| C | 3.653739114 | 2.475034491 | -1.555165256 |
| H | 3.670527697 | 1.771706909 | -0.712035919 |
| H | 3.389211234 | 3.471323885 | -1.166806105 |
| H | 4.678560216 | 2.541462026 | -1.953011406 |
| C | -5.484939225 | -1.319431238 | 0.042392918 |
| H | -5.555905387 | -0.750906533 | -0.900986445 |
| H | -6.443353996 | -1.863123596 | 0.150045752 |
| H | -5.450057819 | -0.590265915 | 0.870696080 |
| Zr | -0.000002370 | 1.198491741 | -0.000006360 |
| As | 1.776123094 | -0.880668737 | -0.008355569 |
| As | -0.004400878 | -2.133826970 | -1.229486912 |
| Al | 3.997337061 | -2.638522072 | -0.074400166 |
| C | -0.178339844 | 1.194630260 | 2.672578813 |
| H | -0.329278765 | 0.289291431 | 3.255299809 |
| C | 2.448442339 | 0.018330136 | 3.646567546 |
| H | 2.388475285 | -0.735910877 | 2.849393326 |
| H | 1.628932649 | -0.167622202 | 4.357826595 |
| H | 3.392848264 | -0.146912904 | 4.188152392 |
| C | -1.225687922 | 2.037567332 | 2.196420484 |
| C | 0.823457797 | 2.942550710 | 1.611736198 |
| H | 1.567573488 | 3.655240147 | 1.261730655 |
| C | -0.586594870 | 3.112961147 | 1.514393890 |
| H | -1.083453655 | 3.972933825 | 1.070853490 |
| C | 1.090793376 | 1.767668551 | 2.376164405 |
| C | 2.485197155 | 2.437732273 | 4.287294501 |
| H | 3.399323086 | 2.252848167 | 4.874670225 |
| H | 1.619193782 | 2.316247803 | 4.956600923 |
| H | 2.504851269 | 3.484609568 | 3.946561017 |
| C | 3.628070144 | -3.541342264 | -1.805540999 |
| H | 4.420061443 | -4.291981583 | -1.993192411 |
| H | 2.671424424 | -4.092570133 | -1.821324425 |
| H | 3.634270387 | -2.859223328 | -2.673511033 |
| C | -3.110783826 | 0.624884331 | 3.166361015 |
| H | -3.036255094 | -0.143841068 | 2.383120133 |
| H | -4.159023376 | 0.651890574 | 3.502251866 |
| H | -2.502295364 | 0.297372766 | 4.023314208 |
| C | -2.777430295 | 3.012459819 | 3.843434495 |
| H | -3.800678598 | 3.024783812 | 4.252894287 |
| H | -2.524806072 | 4.036330842 | 3.526780067 |
| H | -2.089163812 | 2.730026221 | 4.655285456 |
| C | 3.646069558 | -3.671923036 | 1.584754789 |
| H | 3.685488373 | -3.063251051 | 2.504799647 |
| H | 2.677826574 | -4.202711620 | 1.576638612 |
| H | 4.424417027 | -4.451814179 | 1.693068900 |
| C | 2.411146466 | 1.452417603 | 3.089878699 |
| C | -2.686200095 | 2.014090483 | 2.658947414 |
| C | 3.639728935 | 1.684095244 | 2.194487600 |
| H | 3.658019941 | 2.700854770 | 1.772805116 |
| H | 3.676788879 | 0.965868359 | 1.363293586 |
| H | 4.563205365 | 1.556313459 | 2.780766175 |
| C | -3.653750910 | 2.475088943 | 1.555188058 |
| H | -3.670554759 | 1.771789408 | 0.712036358 |
| H | -3.389210350 | 3.471386539 | 1.166858837 |
| H | -4.678568572 | 2.541519029 | 1.953042717 |
| C | 5.484943459 | -1.319408181 | -0.042347785 |
| H | 5.555886707 | -0.750884731 | 0.901034261 |
| H | 6.443363204 | -1.863095936 | -0.149980489 |
| H | 5.450076975 | -0.590241409 | -0.870650227 |
| H | -0.558231086 | -2.934396798 | 2.261943888 |
| H | -0.009928476 | -4.394302730 | 3.107695182 |
| H | -0.038643351 | -4.364600868 | 1.336488949 |
| C | 3.412417763 | -3.686532800 | -2.910038372 |
| H | 4.421363177 | -3.432757336 | -2.549452481 |
| H | 3.495213274 | -4.001451092 | -3.963285299 |
| H | 3.051464024 | -4.546627168 | -2.324847913 |
| C | 2.566238634 | -4.478187738 | 2.255872553 |
| H | 2.425594011 | -5.088217174 | 1.350128577 |
| H | 2.356938274 | -5.114125603 | 3.131649237 |
| H | 3.623887792 | -4.174960723 | 2.298040873 |
| C | 1.074223068 | -2.898393488 | -3.335989867 |
| H | 0.658669631 | -3.762917716 | -2.796218040 |
| H | 1.168898816 | -3.188686072 | -4.394254760 |
| H | 0.340088797 | -2.079987360 | -3.283201119 |
| C | 1.863502625 | -2.448373880 | 3.545063502 |
| H | 2.901663862 | -2.090734230 | 3.623205645 |
| H | 1.671774781 | -3.088151640 | 4.420958499 |
| H | 1.194726751 | -1.577987474 | 3.617623503 |
| C | 3.001614517 | -1.315371797 | -3.623066239 |
| H | 2.347854566 | -0.434177658 | -3.566772501 |
| H | 3.079401350 | -1.612390462 | -4.681032062 |
| H | 4.009286540 | -1.015412537 | -3.294579266 |
| Zr | 1.475561090 | 0.000251885 | 0.000282186 |
| As | -0.559947588 | 0.055645371 | -1.830450066 |
| As | -1.740325788 | -1.215606238 | -0.044866230 |
| Al | -4.138275569 | -2.963636921 | -0.148607791 |
| C | 1.495182956 | 2.674042189 | 0.325207145 |
| H | 0.610470299 | 3.259720715 | 0.563963388 |
| C | 1.993320795 | 2.447771956 | -0.990258028 |
| C | 2.388364592 | 2.126718527 | 1.293592619 |
| C | 3.176757031 | 1.666330854 | -0.832874051 |
| H | 3.837372521 | 1.347692959 | -1.637321082 |
| C | 2.443806085 | 2.479771035 | 2.783581928 |
| C | 3.425591387 | 1.487020778 | 0.556228160 |
| H | 4.305054510 | 1.004111973 | 0.976790128 |
| C | 1.623804789 | 3.245155381 | -2.249066010 |
| C | 0.171755699 | 3.755303199 | -2.229425163 |
| H | -0.557770484 | 2.933856598 | -2.263337228 |
| H | -0.009359221 | 4.394274239 | -3.108110300 |
| H | -0.039839088 | 4.364043332 | -1.336942697 |
| C | 3.408038683 | 3.687985445 | 2.912012119 |
| H | 4.417397940 | 3.435006551 | 2.552024939 |
| H | 3.489961603 | 4.002974238 | 3.965307428 |
| H | 3.046747356 | 4.547795623 | 2.326611951 |
| C | 2.565869877 | 4.479292586 | -2.253817070 |
| H | 2.424009755 | 5.089042236 | -1.348075269 |
| H | 2.357060906 | 5.115310970 | -3.129651344 |
| H | 3.623728345 | 4.176666886 | -2.295030246 |
| C | 1.070195310 | 2.898099911 | 3.336495269 |
| H | 0.654256171 | 3.762176164 | 2.796306122 |
| H | 1.163996655 | 3.188674134 | 4.394760934 |
| H | 0.336770369 | 2.079095490 | 3.283429718 |
| C | 1.865494776 | 2.449363462 | -3.544121921 |
| H | 2.903893804 | 2.092198485 | -3.621285653 |
| H | 1.674387355 | 3.089270807 | -4.420057691 |
| H | 1.197173836 | 1.578704589 | -3.617601055 |
| C | 2.998590502 | 1.316537728 | 3.624898526 |
| H | 2.345627899 | 0.434781246 | 3.568096597 |
| H | 3.075314551 | 1.613616053 | 4.682924817 |
| H | 4.006770879 | 1.017446361 | 3.297180691 |
| C | -4.591656718 | 2.797852621 | -1.769870182 |
| H | -3.802498756 | 3.176717840 | -2.441878530 |
| H | -4.822254499 | 1.761952982 | -2.071288321 |
| H | -5.498694130 | 3.396332939 | -1.982300668 |
| C | -5.151305850 | 1.919587042 | 1.488305499 |
| H | -6.118129332 | 2.422053159 | 1.685203759 |
| H | -5.392036233 | 0.897766703 | 1.149399751 |
| H | -4.636399824 | 1.839700518 | 2.460908051 |
| C | -3.141120488 | 4.567856034 | 0.756612455 |
| H | -2.389649530 | 4.918225937 | 0.027715868 |
| H | -3.850079447 | 5.406180611 | 0.899181389 |
| H | -2.634177590 | 4.423754989 | 1.726727700 |
| C | -3.144110049 | -4.569067041 | -0.758185863 |
| H | -2.394934672 | -4.923084530 | -0.028697161 |
| H | -3.855009628 | -5.405155013 | -0.904181199 |
| H | -2.634915448 | -4.424117809 | -1.727006164 |
| C | -5.152692162 | -1.919024108 | -1.486905719 |
| H | -6.118911879 | -2.422322404 | -1.684621156 |
| H | -5.394549189 | -0.897975331 | -1.146489932 |
| H | -4.637867243 | -1.837151154 | -2.459389826 |
| C | -4.588632433 | -2.797428268 | 1.770593621 |
| H | -4.820660014 | -1.761806886 | 2.071851556 |
| H | -5.494112725 | -3.397587628 | 1.984945163 |
| H | -3.797589441 | -3.174438477 | 2.441433540 |

**Table S15:** Cartesian coordinates of the optimized geometry of the Isomers for [Cp''$_2$Zr(μ,η$^{1:1:1}$-As$_4$)(AlMe$_3$)] at the B3LYP/def2- SVP level of theory.

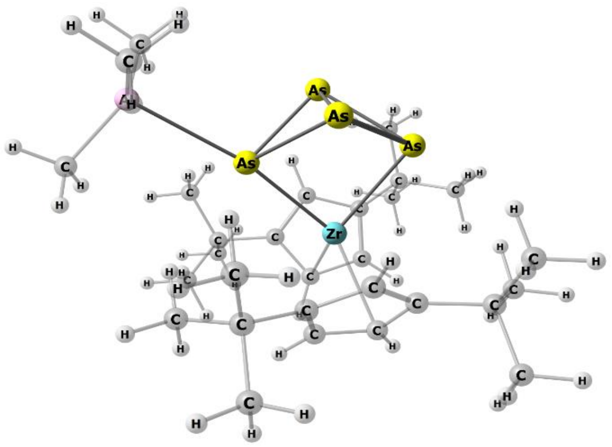

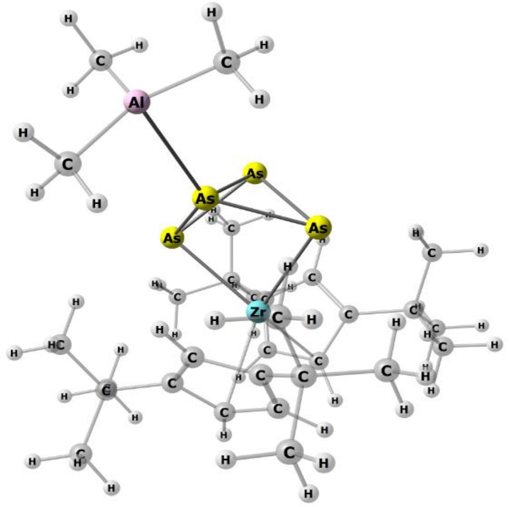

| | | | |
|---|---|---|---|
| As | 1.287504482 | -0.031353714 | -2.123954713 |
| As | -0.941737221 | 1.072696942 | -2.321526140 |
| C | 0.734600936 | -2.593766948 | 0.843503233 |
| H | 0.093437601 | -3.235099912 | 0.243876550 |
| C | 2.219963389 | -3.748220474 | -1.552320669 |
| H | 1.717844730 | -3.021105607 | -2.205393959 |
| H | 1.459772833 | -4.442569024 | -1.162394889 |
| H | 2.909618211 | -4.337867902 | -2.176915143 |
| C | 0.369508105 | -2.033364558 | 2.103781497 |
| C | 2.543366927 | -1.431840424 | 1.591909768 |
| H | 3.551875912 | -1.036725997 | 1.698163702 |
| C | 1.502502515 | -1.297347094 | 2.551837589 |
| H | 1.589710941 | -0.774940319 | 3.502131785 |
| C | 2.091310716 | -2.284075689 | 0.540299518 |
| C | 3.665395164 | -4.172027017 | 0.442877658 |
| H | 4.316265098 | -4.804349120 | -0.183390521 |
| H | 2.905906821 | -4.819540608 | 0.908145885 |
| H | 4.280494754 | -3.741713102 | 1.248334623 |
| C | -1.974654708 | -3.024784934 | 2.123531387 |
| H | -2.355690998 | -2.299890157 | 1.389563575 |
| H | -2.816019043 | -3.309442161 | 2.774349261 |
| H | -1.663256752 | -3.928989682 | 1.578258254 |
| C | -0.318106271 | -3.549894879 | 3.925935942 |
| H | -1.141862229 | -3.913483640 | 4.561897223 |
| H | 0.481412327 | -3.175934086 | 4.584395088 |
| H | 0.081731259 | -4.407673494 | 3.362933245 |
| C | 3.003331443 | -3.062936397 | -0.418455596 |
| C | -0.828028232 | -2.441712670 | 2.967526416 |
| C | 4.119041574 | -2.192609640 | -1.023412718 |
| H | 4.711775979 | -1.684252516 | -0.247656490 |
| H | 3.713361224 | -1.428286056 | -1.702435056 |
| H | 4.811921953 | -2.821041856 | -1.605501410 |
| C | -1.358165245 | -1.269907375 | 3.813238514 |
| H | -1.776558395 | -0.471674285 | 3.185263719 |
| H | -0.569494147 | -0.831903874 | 4.445579206 |
| H | -2.158319314 | -1.617963958 | 4.485537391 |
| Zr | 0.796232803 | 0.073229501 | 0.556943280 |
| As | -1.792196853 | -0.091179058 | -0.270969732 |
| As | -0.822953460 | -1.369330567 | -2.189573560 |
| Al | -4.572770054 | -0.292605516 | -0.575140945 |
| C | 0.904061451 | 2.749961937 | 0.311780939 |
| H | 0.583034015 | 3.278641548 | -0.581901094 |
| C | -2.002777970 | 3.660477356 | 0.530183490 |
| H | -2.328623878 | 2.806579558 | -0.079739258 |
| H | -1.401372456 | 4.327983469 | -0.106305291 |
| H | -2.905315640 | 4.218551757 | 0.824811359 |
| C | 2.235398230 | 2.298392813 | 0.553946015 |
| C | 0.906065446 | 1.805949836 | 2.383888261 |
| H | 0.606777335 | 1.496324116 | 3.383432656 |

| | | | |
|---|---|---|---|
| As | -0.512646515 | -0.269833241 | -2.010681174 |
| As | -0.515440055 | -2.471310438 | -0.812143173 |
| C | -0.892919026 | 2.276917207 | 0.995245047 |
| H | -1.939596461 | 2.020377966 | 1.139840259 |
| C | -0.348382821 | 2.834386431 | -0.196466105 |
| C | 0.107038087 | 2.202914891 | 2.009239315 |
| C | 1.041503319 | 3.029697228 | 0.060611383 |
| H | 1.756807548 | 3.480305155 | -0.624969756 |
| C | -0.136178486 | 2.056313833 | 3.515386347 |
| C | 1.313990518 | 2.660429417 | 1.406956790 |
| H | 2.272181764 | 2.781418423 | 1.907521171 |
| C | -1.141762749 | 3.510175893 | -1.323869548 |
| C | -2.546778824 | 2.908871753 | -1.506161564 |
| H | -2.508602627 | 1.862326740 | -1.838814257 |
| H | -3.099706791 | 3.479576081 | -2.268999789 |
| H | -3.136728486 | 2.951537712 | -0.577716074 |
| C | -0.245491920 | 3.496456825 | 4.082297067 |
| H | 0.685566187 | 4.062527412 | 3.923503415 |
| H | -0.446776327 | 3.467669313 | 5.165967420 |
| H | -1.064277822 | 4.051659567 | 3.598624732 |
| C | -1.306786447 | 4.991783860 | -0.890688301 |
| H | -1.842310494 | 5.065772338 | 0.068627036 |
| H | -1.883149696 | 5.549866363 | -1.646972423 |
| H | -0.330271928 | 5.486363823 | -0.771448352 |
| C | -1.451029153 | 1.318161101 | 3.823245938 |
| H | -2.323055071 | 1.839120984 | 3.399272571 |
| H | -1.602924285 | 1.261011599 | 4.912781936 |
| H | -1.448190307 | 0.289051365 | 3.433637585 |
| C | -0.397770921 | 3.481766949 | -2.670798904 |
| H | 0.604197115 | 3.931826167 | -2.597116047 |
| H | -0.960044240 | 4.056948423 | -3.423455114 |
| H | -0.286007229 | 2.455892536 | -3.051809043 |
| C | 1.028804320 | 1.340926126 | 4.223212867 |
| H | 1.130436885 | 0.300497302 | 3.884951948 |
| H | 0.859502907 | 1.326068205 | 5.311820793 |
| H | 1.989094558 | 1.852585709 | 4.050809729 |
| Zr | 0.955790337 | 0.507620811 | 0.152687729 |
| As | -0.468457693 | -1.408814249 | 1.465500420 |
| As | -2.177329012 | -0.789166461 | -0.232353847 |
| Al | -5.042577536 | -1.316326761 | -0.316626216 |
| C | 2.846181151 | -1.217105159 | -0.696070816 |
| H | 2.616355720 | -2.140715482 | -1.221537569 |
| C | 3.035075394 | -1.107313707 | 0.711276258 |
| C | 3.110517284 | 0.033230858 | -1.329736785 |
| C | 3.339908520 | 0.263799908 | 0.961305491 |
| H | 3.584185131 | 0.693697821 | 1.931006650 |
| C | 3.412974896 | 0.255882682 | -2.815113324 |
| C | 3.401737235 | 0.952174670 | -0.281111010 |
| H | 3.695413241 | 1.991967953 | -0.406550897 |

| | | | |
|---|---|---|---|
| C | 2.218150121 | 1.697610965 | 1.845667595 |
| H | 3.078984602 | 1.288738984 | 2.370728374 |
| C | 0.084953804 | 2.505574479 | 1.449651800 |
| C | -0.831403388 | 4.489390891 | 2.579035500 |
| H | -1.729303423 | 5.070834681 | 2.845394339 |
| H | -0.170209781 | 5.136765710 | 1.981951184 |
| H | -0.302512424 | 4.233746759 | 3.510401677 |
| C | -4.691367417 | -2.102791396 | -1.389028000 |
| H | -5.755848184 | -2.348004106 | -1.569869356 |
| H | -4.184442622 | -2.181024823 | -2.366843361 |
| H | -4.292566684 | -2.904102676 | -0.742598831 |
| C | 3.164025926 | 3.187661357 | -1.643436423 |
| H | 2.697915582 | 2.388631567 | -2.239263891 |
| H | 4.087159115 | 3.494591427 | -2.160112516 |
| H | 2.484910817 | 4.054085987 | -1.644763018 |
| C | 4.093889044 | 3.924412346 | 0.561409171 |
| H | 4.986928389 | 4.307652659 | 0.040686873 |
| H | 4.392284125 | 3.636585130 | 1.581473504 |
| H | 3.367071321 | 4.747672712 | 0.642926767 |
| C | -4.903049318 | 1.266857049 | -1.762939484 |
| H | -4.619508846 | 2.233410524 | -1.310785439 |
| H | -4.389712286 | 1.192969147 | -2.737823706 |
| H | -5.985636261 | 1.331821182 | -1.985359805 |
| C | -1.232432947 | 3.215970943 | 1.786189263 |
| C | 3.492626233 | 2.722628953 | -0.213680415 |
| C | -2.153870362 | 2.363016393 | 2.674706937 |
| H | -1.652015556 | 2.045130941 | 3.601451338 |
| H | -2.509233348 | 1.464841145 | 2.149549233 |
| H | -3.041055314 | 2.946226979 | 2.967518240 |
| C | 4.546590904 | 1.602175505 | -0.272497200 |
| H | 4.193998547 | 0.747336555 | -0.865751728 |
| H | 4.812715682 | 1.236133902 | 0.731835456 |
| H | 5.472149329 | 1.975902381 | -0.739144748 |
| C | -5.145990265 | -0.133335465 | 1.324091987 |
| H | -4.975670492 | 0.866081993 | 1.760457913 |
| H | -6.236148135 | -0.317381133 | 1.383580512 |
| H | -4.671957582 | -0.873893120 | 1.991886608 |

| | | | |
|---|---|---|---|
| C | 3.311842165 | -2.263507692 | 1.682925524 |
| C | 2.673555791 | -3.587567389 | 1.225586045 |
| H | 1.575703630 | -3.535610687 | 1.214194674 |
| H | 2.961043397 | -4.397096087 | 1.915183668 |
| H | 3.013324347 | -3.881148751 | 0.220244086 |
| C | 4.951310622 | 0.133419282 | -2.971901315 |
| H | 5.476064658 | 0.895772647 | -2.375391039 |
| H | 5.241959104 | 0.265704356 | -4.027281837 |
| H | 5.306339470 | -0.855468459 | -2.642096680 |
| C | 4.852763684 | -2.450542496 | 1.688630558 |
| H | 5.228856157 | -2.674420900 | 0.678149704 |
| H | 5.134921808 | -3.285495986 | 2.351128829 |
| H | 5.365293837 | -1.543697090 | 2.045461317 |
| C | 2.751563222 | -0.808028333 | -3.708880500 |
| H | 3.086916334 | -1.824762252 | -3.452288612 |
| H | 3.018741095 | -0.632251658 | -4.763021641 |
| H | 1.653668410 | -0.783804874 | -3.635823776 |
| C | 2.859525144 | -1.951506479 | 3.120685822 |
| H | 3.307062642 | -1.020173296 | 3.500025723 |
| H | 3.169605581 | -2.763082395 | 3.798115215 |
| H | 1.765579761 | -1.859461245 | 3.191438307 |
| C | 2.982895009 | 1.656734674 | -3.285616173 |
| H | 1.895072726 | 1.790554409 | -3.210304365 |
| H | 3.270619385 | 1.810310257 | -4.337930239 |
| H | 3.466190633 | 2.452461144 | -2.696866837 |
| C | -5.576440428 | 0.219199364 | 0.821846791 |
| H | -5.317553371 | 1.199893791 | 0.385782139 |
| H | -6.676247390 | 0.216094108 | 0.949142315 |
| H | -5.146793746 | 0.176144929 | 1.837841423 |
| C | -4.983178422 | -3.117426857 | 0.503983812 |
| H | -6.012941066 | -3.515935781 | 0.586872381 |
| H | -4.410101840 | -3.845948637 | -0.094498576 |
| H | -4.564122879 | -3.117446617 | 1.524835509 |
| C | -5.258090998 | -1.154999954 | -2.279167491 |
| H | -4.675007961 | -1.901728109 | -2.844654814 |
| H | -6.320955722 | -1.322521749 | -2.540829532 |
| H | -4.993883022 | -0.157183470 | -2.669524323 |

**Table S16:** Cartesian coordinates of the optimized geometry of the Isomers for $[Cp''_2Zr(\mu,\eta^{1:1:1:1}-As_4)(AlEt_3)_2]$ at the B3LYP/def2- SVP level of theory.

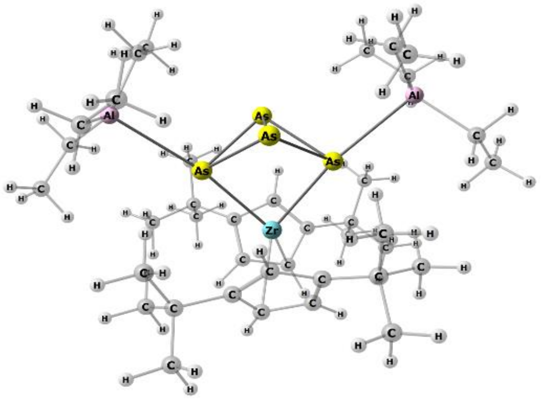

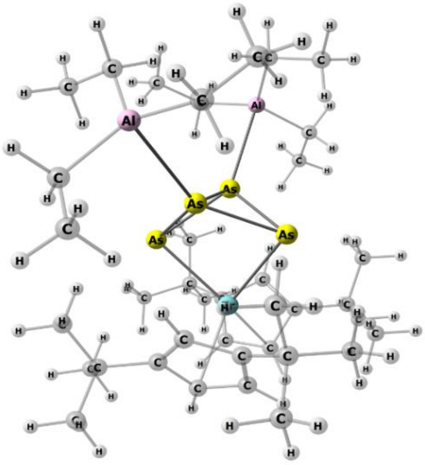

| | | | |
|---|---|---|---|
| As | 1.789479893 | 0.610237842 | -0.036213091 |
| As | -0.022145310 | 1.849806301 | -1.226861404 |
| Al | 4.016914730 | 2.441809060 | -0.087343100 |
| C | -0.237659840 | -1.502037760 | 2.673848347 |
| H | -0.462944310 | -0.618770175 | 3.266554859 |
| C | 1.071694585 | -1.993504383 | 2.407564307 |
| C | -1.216930621 | -2.403377388 | 2.157938386 |
| C | 0.898299293 | -3.171471550 | 1.621040510 |
| H | 1.696872139 | -3.826204420 | 1.277363054 |
| C | -2.693507710 | -2.472121465 | 2.558304685 |
| C | -0.494346371 | -3.430183291 | 1.487394626 |
| H | -0.924926048 | -4.311627273 | 1.017604548 |
| C | 2.350620427 | -1.624056836 | 3.168894277 |
| C | 2.341317152 | -0.173420242 | 3.681005607 |
| H | 2.340249939 | 0.555606885 | 2.859334460 |
| H | 3.241859099 | 0.013563655 | 4.286414046 |
| H | 1.470243388 | 0.030552432 | 4.322763014 |
| C | -2.774920275 | -3.462023372 | 3.750102794 |

| | | | |
|---|---|---|---|
| As | 0.183572246 | -0.126844455 | 1.823585526 |
| As | 1.370336877 | -1.215327462 | -0.075460724 |
| Al | 3.666100663 | -3.193206458 | 0.193291734 |
| C | -1.862711145 | 2.687074846 | 0.038614387 |
| H | -0.966556189 | 3.290140707 | -0.083956544 |
| C | -2.406927056 | 2.284079632 | 1.292367351 |
| C | -2.723871022 | 2.289037710 | -1.026216071 |
| C | -3.586926735 | 1.542675741 | 0.987688343 |
| H | -4.277966514 | 1.122981275 | 1.716607101 |
| C | -2.727353511 | 2.854232366 | -2.450560959 |
| C | -3.786302937 | 1.557884761 | -0.420844663 |
| H | -4.652411288 | 1.146186401 | -0.934225867 |
| C | -2.076190481 | 2.894153646 | 2.661868784 |
| C | -0.641024390 | 3.445215835 | 2.733454586 |
| H | 0.113414144 | 2.653468498 | 2.622839904 |
| H | -0.474922430 | 3.926928647 | 3.709899548 |
| H | -0.453576679 | 4.205950740 | 1.960645894 |
| C | -3.667203961 | 4.088524377 | -2.426358212 |

| | | | |
|---|---|---|---|
| H | -2.426600416 | -4.466311503 | 3.462882142 |
| H | -3.815549436 | -3.549273509 | 4.102743094 |
| H | -2.157158469 | -3.118739714 | 4.594529139 |
| C | 2.387324309 | -2.568512048 | 4.401046803 |
| H | 1.499230908 | -2.427459895 | 5.036482166 |
| H | 3.280937076 | -2.360257263 | 5.011695355 |
| H | 2.420806401 | -3.625976925 | 4.096147614 |
| C | -3.233093754 | -1.109593911 | 3.023837019 |
| H | -2.672740006 | -0.718871444 | 3.886905390 |
| H | -4.284826540 | -1.206902728 | 3.334756112 |
| H | -3.195924291 | -0.354046320 | 2.225456905 |
| C | 3.620505926 | -1.868015650 | 2.336169252 |
| H | 3.690333806 | -2.909878767 | 1.987598907 |
| H | 4.515747236 | -1.667170960 | 2.944772408 |
| H | 3.666382699 | -1.207674332 | 1.458946528 |
| C | -3.571840870 | -3.007410854 | 1.413580411 |
| H | -3.552414832 | -2.335052874 | 0.544975830 |
| H | -4.618034230 | -3.095913627 | 1.745964336 |
| H | -3.249973995 | -4.007303194 | 1.081728374 |
| C | 3.310208982 | 3.403050500 | -2.907652873 |
| H | 3.654447752 | 3.569280938 | -3.944933681 |
| H | 3.064880043 | 4.394573880 | -2.490252528 |
| H | 2.356687280 | 2.850221277 | -2.978494535 |
| C | 3.157697225 | 3.987400997 | 2.390978701 |
| H | 2.766469637 | 4.927976996 | 2.819576067 |
| H | 4.158314844 | 3.828042718 | 2.828191437 |
| H | 2.516268723 | 3.175755573 | 2.777554440 |
| C | 6.181176938 | 0.313610479 | 0.165323044 |
| H | 6.683086795 | 0.720787018 | -0.728941406 |
| H | 5.526816620 | -0.501135740 | -0.191601783 |
| H | 6.965704224 | -0.159691296 | 0.784029414 |
| C | 5.394473623 | 1.398190937 | 0.917791654 |
| H | 6.103571983 | 2.169401096 | 1.284564580 |
| H | 4.956887700 | 0.977881783 | 1.842249882 |
| C | 4.340648919 | 2.657439147 | -2.046173394 |
| H | 5.300230224 | 3.217490465 | -2.070769320 |
| H | 4.589577321 | 1.685008562 | -2.509895307 |
| C | 3.201759475 | 4.001903401 | 0.854779624 |
| H | 2.195742396 | 4.221914663 | 0.449534224 |
| Zr | 0.001064187 | -1.465709212 | 0.000663721 |
| As | -1.790339764 | 0.607591205 | 0.036231443 |
| As | 0.019416190 | 1.849910490 | 1.227014532 |
| Al | -4.020752869 | 2.437751419 | 0.085335449 |
| C | 0.240050955 | -1.503124213 | -2.672493288 |
| H | 0.464348510 | -0.619892131 | -3.265632021 |
| C | -1.068762986 | -1.995990251 | -2.406153526 |
| C | 1.220298119 | -2.403055870 | -2.155947505 |
| C | -0.894124217 | -3.173286861 | -1.618898935 |
| H | -1.691997035 | -3.828742391 | -1.274959960 |
| C | 2.696997687 | -2.470481217 | -2.556124888 |
| C | 0.498805346 | -3.430331095 | -1.484957442 |
| H | 0.930342542 | -4.311040632 | -1.014675763 |
| C | -2.347984039 | -1.628490225 | -3.167936381 |
| C | -2.340306609 | -0.178144109 | -3.680895914 |
| H | -2.340262962 | 0.551386554 | -2.859667895 |
| H | -3.240967192 | 0.007409769 | -4.286567539 |
| H | -1.469369055 | 0.026503937 | -4.322621470 |
| C | 2.779381589 | -3.460396528 | -3.747847547 |
| H | 2.431676887 | -4.464904812 | -3.460651745 |
| H | 3.820154802 | -3.546952897 | -4.100230370 |
| H | 2.161589074 | -3.117579398 | -4.592440493 |
| C | -2.383294847 | -2.573711337 | -4.399548390 |
| H | -1.495170944 | -2.432043544 | -5.034803288 |
| H | -3.276962823 | -2.366787448 | -5.010564689 |
| H | -2.415694681 | -3.631036087 | -4.094044341 |
| C | 3.235579336 | -1.107559420 | -3.021626889 |
| H | 2.674519740 | -0.716813796 | -3.884218905 |
| H | 4.287161038 | -1.204282962 | -3.333246872 |
| H | 3.198647749 | -0.352289103 | -2.222983155 |
| C | -3.617758110 | -1.873448464 | -2.335341889 |
| H | -3.686290913 | -2.915101408 | -1.985887786 |
| H | -4.513102593 | -1.674330590 | -2.944364492 |
| H | -3.664775016 | -1.212395196 | -1.458720204 |
| C | 3.575644829 | -3.004895252 | -1.411241252 |
| H | 3.555357597 | -2.332506298 | -0.542677312 |
| H | 4.621987507 | -3.092352587 | -1.743433826 |
| H | 3.254737691 | -4.005100187 | -1.079407900 |
| C | -3.316757635 | 3.421490660 | 2.897108043 |
| H | -3.658509150 | 3.589065378 | 3.934989488 |
| H | -3.084682909 | 4.413601274 | 2.473553938 |
| H | -2.356621286 | 2.880114198 | 2.966570992 |
| C | -3.164919066 | 3.977486697 | -2.398161954 |
| H | -2.775098491 | 4.917436293 | -2.829407354 |
| H | -4.165580967 | 3.815763456 | -2.834411609 |
| H | -2.522704852 | 3.165645674 | -2.782988725 |
| C | -6.179414884 | 0.302795915 | -0.160150394 |
| H | -6.679731441 | 0.709003087 | 0.735450795 |
| H | -5.521983167 | -0.510208892 | 0.195075191 |

| | | | |
|---|---|---|---|
| H | -4.691740720 | 3.807100836 | -2.137113790 |
| H | -3.710178001 | 4.556601011 | -3.423551842 |
| H | -3.309653097 | 4.844776415 | -1.710358470 |
| C | -3.056202912 | 4.085402311 | 2.838238053 |
| H | -2.937612471 | 4.820181738 | 2.026660565 |
| H | -2.864415859 | 4.599832813 | 3.794309478 |
| H | -4.104231300 | 3.747565008 | 2.837094167 |
| C | -1.330748296 | 3.321762318 | -2.897227171 |
| H | -0.922791352 | 4.098104956 | -2.232442680 |
| H | -1.385378053 | 3.754332608 | -3.908708217 |
| H | -0.607586749 | 2.492461843 | -2.932111038 |
| C | -2.300219294 | 1.909643817 | 3.823177230 |
| H | -3.319261209 | 1.493776117 | 3.821336804 |
| H | -2.159382506 | 2.426143064 | 4.785840352 |
| H | -1.587304412 | 1.072474138 | 3.789330222 |
| C | -3.277681096 | 1.840347130 | -3.469393954 |
| H | -2.639177315 | 0.948879981 | -3.538278677 |
| H | -3.327753414 | 2.296761137 | -4.470771440 |
| H | -4.296685837 | 1.511439800 | -3.211094379 |
| C | 4.674040884 | -1.722456139 | 2.641944425 |
| H | 5.459556762 | -1.096764019 | 3.101697979 |
| H | 4.619397390 | -2.653489749 | 3.231873863 |
| H | 3.716619358 | -1.196336603 | 2.797762936 |
| C | 1.641476081 | -5.440507651 | 0.551914164 |
| H | 1.149840016 | -6.159419498 | 1.232260473 |
| H | 2.147098116 | -6.033599881 | -0.228835845 |
| H | 0.835849170 | -4.884276247 | 0.040883395 |
| C | 4.691603441 | -2.535429882 | -2.589279234 |
| H | 5.692812273 | -2.328026458 | -2.174769133 |
| H | 4.166223462 | -1.566511604 | -2.634711651 |
| H | 4.839608232 | -2.866978997 | -3.632789146 |
| C | 3.925489095 | -3.565175518 | -1.742883577 |
| H | 4.462628096 | -4.537040916 | -1.771108314 |
| H | 2.947230119 | -3.789548857 | -2.209246640 |
| C | 4.935679816 | -2.003803579 | 1.153418492 |
| H | 5.924696262 | -2.495984507 | 1.039392918 |
| H | 5.045095796 | -1.053763536 | 0.598024077 |
| C | 2.614754329 | -4.498000473 | 1.275272497 |
| H | 3.382825097 | -5.101608312 | 1.803011395 |
| Zr | -1.854230656 | 0.000071365 | -0.000070774 |
| As | 0.183617792 | 0.126932303 | -1.823650768 |
| As | 1.370365506 | 1.215358202 | 0.075471168 |
| Al | 3.666072866 | 3.193076264 | -0.193157589 |
| C | -1.862823511 | -2.687136045 | -0.038517357 |
| H | -0.966716456 | -3.290252617 | 0.084187770 |
| C | -2.406878086 | -2.284202173 | -1.292347068 |
| C | -2.724061775 | -2.288928213 | 1.026192592 |
| C | -3.586822455 | -1.542629682 | -0.987863008 |
| H | -4.277854773 | -1.123092254 | -1.716878738 |
| C | -2.727727174 | -2.854078344 | 2.450550100 |
| C | -3.786367173 | -1.557747900 | 0.420650641 |
| H | -4.652476758 | -1.145911361 | 0.933909228 |
| C | -2.076019213 | -2.894281056 | -2.661808495 |
| C | -0.640857012 | -3.445353157 | -2.733247519 |
| H | 0.113574460 | -2.653619737 | -2.622482524 |
| H | -0.474617674 | -3.927014522 | -3.709694650 |
| H | -0.453540888 | -4.206128745 | -1.960451866 |
| C | -3.667457050 | -4.088462013 | 2.426237803 |
| H | -4.691978924 | -3.807140206 | 2.136843799 |
| H | -3.710529348 | -4.556521746 | 3.423435349 |
| H | -3.309734399 | -4.844693667 | 1.710303070 |
| C | -3.056030750 | -4.085530964 | -2.838270856 |
| H | -2.937546964 | -4.820284917 | -2.026655326 |
| H | -2.864131383 | -4.599989804 | -3.794303991 |
| H | -4.104053681 | -3.747681089 | -2.837264652 |
| C | -1.331142518 | -3.321461883 | 2.897455956 |
| H | -0.923040378 | -4.097835214 | 2.232793519 |
| H | -1.385880337 | -3.753946759 | 3.908966937 |
| H | -0.608043815 | -2.492109411 | 2.932355073 |
| C | -2.299955828 | -1.909788669 | -3.823152337 |
| H | -3.318988127 | -1.493897786 | -3.821375418 |
| H | -2.159082467 | -2.426330525 | -4.785787159 |
| H | -1.587028066 | -1.072631674 | -3.789314685 |
| C | -3.278326491 | -1.840211449 | 3.469242350 |
| H | -2.639919872 | -0.948688234 | 3.538224415 |
| H | -3.328560833 | -2.296591345 | 4.470627742 |
| H | -4.297310869 | -1.511402726 | 3.210729104 |
| C | 1.641274110 | 5.440504000 | -0.551178047 |
| H | 1.150013104 | 6.159861837 | -1.231317108 |
| H | 2.146435136 | 6.033068693 | 0.230268515 |
| H | 0.835363793 | 4.883894229 | -0.040993244 |
| C | 4.693139229 | 2.534962772 | 2.588719984 |
| H | 4.841595626 | 2.866266164 | 3.632252158 |
| H | 5.694223271 | 2.328296487 | 2.173533002 |
| H | 4.168427676 | 1.565688379 | 2.634203138 |
| C | 4.673225274 | 1.722243140 | -2.642179364 |
| H | 4.618162185 | 2.653139011 | -3.232294606 |
| H | 3.715831667 | 1.195915119 | -2.797454219 |

| | | | |
|---|---|---|---|
| H | -6.964565434 | -0.172677011 | -0.776398927 |
| C | -5.397589683 | 1.389015961 | -0.915308077 |
| H | -6.109731659 | 2.157976122 | -1.280944293 |
| H | -4.960994984 | 0.969277176 | -1.840494093 |
| C | -4.342069332 | 2.659508326 | 2.043827578 |
| H | -5.307831167 | 3.208776943 | 2.068779391 |
| H | -4.578296675 | 1.687007285 | 2.514010681 |
| C | -3.208078746 | 3.995892953 | -0.862009207 |
| H | -2.202013437 | 4.217833468 | -0.457902324 |
| H | -3.823539584 | 4.857491137 | -0.527959314 |
| H | 3.816714272 | 4.863164938 | 0.518942802 |

| | | | |
|---|---|---|---|
| H | 5.458645943 | 1.096587692 | -3.102147521 |
| C | 4.935504357 | 2.003959549 | -1.153840236 |
| H | 5.924421936 | 2.496452231 | -1.040389920 |
| H | 5.045456557 | 1.054059669 | -0.598312362 |
| C | 2.614956126 | 4.498481479 | -1.274607813 |
| H | 3.383256392 | 5.102413174 | -1.801646604 |
| H | 2.093881930 | 3.963949364 | -2.091560620 |
| C | 3.925831456 | 3.564463886 | 1.743111552 |
| H | 2.947613074 | 3.787895474 | 2.210009930 |
| H | 4.462254243 | 4.536715377 | 1.771455724 |
| H | 2.093236725 | -3.963039586 | 2.091664688 |

**Table S17:** Cartesian coordinates of the optimized geometry of the Isomers for [Cp''$_2$Zr(µ,η$^{1:1:1}$-As$_4$)(AlEt$_3$)] at the B3LYP/def2- SVP level of theory.

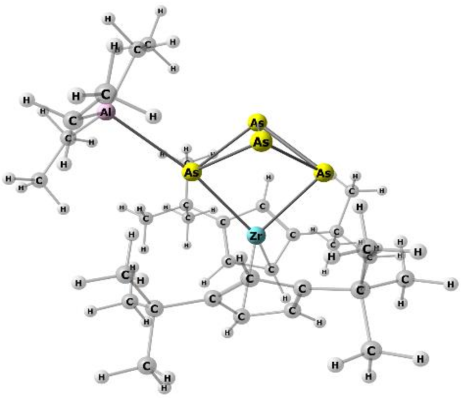

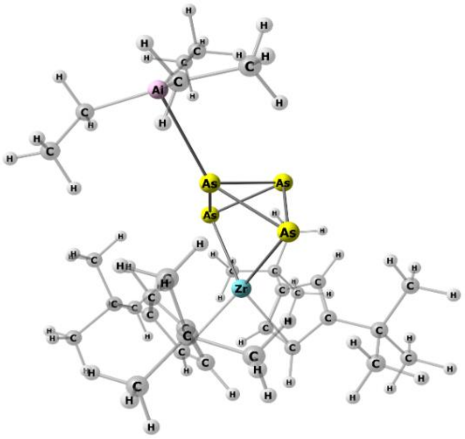

| | | | |
|---|---|---|---|
| As | -1.439685180 | -0.083500557 | -2.154151752 |
| As | 0.751221039 | 1.111538267 | -2.228349430 |
| C | -0.948770267 | -2.598466370 | 0.872783472 |
| H | -0.241432130 | -3.220514454 | 0.330057659 |
| C | -2.293424838 | -2.351294170 | 0.473135783 |
| C | -0.695054208 | -2.005421158 | 2.145574075 |
| C | -2.850687921 | -1.503530346 | 1.476823448 |
| H | -3.878730802 | -1.147822194 | 1.506627615 |
| C | 0.461037337 | -2.339348316 | 3.093194572 |
| C | -1.885280280 | -1.313355325 | 2.503791919 |
| H | -2.059909857 | -0.783314311 | 3.437659300 |
| C | -3.106469073 | -3.182178748 | -0.529710601 |
| C | -2.227988256 | -3.826293962 | -1.617033638 |
| H | -1.740297525 | -3.073809243 | -2.251839640 |
| H | -2.848834462 | -4.461484189 | -2.268675430 |
| H | -1.446310860 | -4.470120039 | -1.185124882 |
| C | -0.066013525 | -3.423366980 | 4.068957662 |
| H | -0.909980320 | -3.048824229 | 4.668942759 |
| H | 0.733427612 | -3.734328401 | 4.761302212 |
| H | -0.409822457 | -4.315523456 | 3.522613898 |
| C | -3.752401381 | -4.322773788 | 0.302193301 |
| H | -2.984965923 | -4.927979922 | 0.809394653 |
| H | -4.335759269 | -4.990212378 | -0.353490324 |
| H | -4.429890037 | -3.922965268 | 1.072574253 |
| C | 1.669388773 | -2.915905064 | 2.336907375 |
| H | 1.417393435 | -3.852883092 | 1.816880633 |
| H | 2.485144724 | -3.141315374 | 3.041255819 |
| H | 2.062500370 | -2.210244885 | 1.590995445 |
| C | -4.234802558 | -2.376613836 | -1.197802937 |
| H | -4.901686798 | -1.910274628 | -0.456738601 |
| H | -4.852974662 | -3.042451575 | -1.820855468 |
| H | -3.837786914 | -1.584659960 | -1.850056776 |
| C | 0.905901671 | -1.115209624 | 3.913785562 |
| H | 1.327760382 | -0.329196001 | 3.272014423 |
| H | 1.681581261 | -1.405697279 | 4.639929589 |
| H | 0.070305858 | -0.680482455 | 4.485088902 |
| Zr | -1.094280072 | 0.057079747 | 0.543040047 |
| As | 1.555624301 | -0.020550746 | -0.134692181 |
| As | 0.724562126 | -1.331320517 | -2.102753983 |
| Al | 4.386564618 | -0.149001851 | -0.372229507 |
| C | -1.338223576 | 2.725818294 | 0.245358154 |
| H | -1.005424692 | 3.257070875 | -0.642429197 |
| C | -0.561444541 | 2.544575155 | 1.423238949 |
| C | -2.653580752 | 2.206975136 | 0.435982822 |
| C | -1.385160862 | 1.813790026 | 2.331363392 |
| H | -1.117895088 | 1.539408987 | 3.350192598 |
| C | -3.896359718 | 2.548818008 | -0.392746665 |
| C | -2.663349502 | 1.627061674 | 1.736776056 |
| H | -3.524680515 | 1.182501698 | 2.230654145 |

| | | | |
|---|---|---|---|
| As | -0.306723353 | -0.363632174 | -1.953216822 |
| As | -0.477699612 | -2.421348374 | -0.536178656 |
| C | -0.357725580 | 2.518026364 | 0.732490868 |
| H | -1.424388798 | 2.389983160 | 0.897091647 |
| C | 0.235614027 | 2.891517444 | -0.507241738 |
| C | 0.633384161 | 2.449850014 | 1.755703631 |
| C | 1.638884340 | 2.970123644 | -0.263108855 |
| H | 2.392683340 | 3.276445600 | -0.986121862 |
| C | 0.381002892 | 2.491656144 | 3.266684047 |
| C | 1.878210249 | 2.715725365 | 1.115778585 |
| H | 2.845575704 | 2.793860566 | 1.607086302 |
| C | -0.490973266 | 3.517566785 | -1.705602034 |
| C | -1.960441917 | 3.070595613 | -1.801153855 |
| H | -2.051297984 | 1.989410345 | -1.976129350 |
| H | -2.453992858 | 3.585962622 | -2.640256926 |
| H | -2.526241306 | 3.317874913 | -0.889954591 |
| C | 0.395341471 | 3.989588076 | 3.669700566 |
| H | 1.369313856 | 4.454000025 | 3.450111122 |
| H | 0.200525863 | 4.098030644 | 4.749507296 |
| H | -0.377806131 | 4.554281696 | 3.125791398 |
| C | -0.467933193 | 5.049898577 | -1.459541784 |
| H | -0.958364335 | 5.306038315 | -0.507466285 |
| H | -1.000021872 | 5.575275126 | -2.269695827 |
| H | 0.562920517 | 5.435152484 | -1.421938295 |
| C | -0.990402805 | 1.903830313 | 3.643823063 |
| H | -1.816941197 | 2.441997643 | 3.155307651 |
| H | -1.146822975 | 1.983709650 | 4.731347933 |
| H | -1.072185170 | 0.840097501 | 3.373380722 |
| C | 0.214254626 | 3.232526428 | -3.043390997 |
| H | 1.271789378 | 3.537755664 | -3.025513675 |
| H | -0.275693268 | 3.794187270 | -3.854652719 |
| H | 0.169262695 | 2.165030621 | -3.304302559 |
| C | 1.482575657 | 1.763027211 | 4.056740615 |
| H | 1.496055797 | 0.688070285 | 3.831246288 |
| H | 1.312773839 | 1.878013385 | 5.139219500 |
| H | 2.482508479 | 2.171040434 | 3.839359528 |
| Zr | 1.291274663 | 0.490107170 | 0.087420606 |
| As | -0.274213925 | -1.141746858 | 1.615100739 |
| As | -1.970290456 | -0.544340998 | -0.104639298 |
| Al | -4.884256176 | -0.850454944 | -0.096106895 |
| C | 2.970160898 | -1.524689440 | -0.547234539 |
| H | 2.630147894 | -2.488298975 | -0.918360747 |
| C | 3.231252005 | -1.227802726 | 0.820599901 |
| C | 3.319412236 | -0.415146841 | -1.371366212 |
| C | 3.674884397 | 0.128053949 | 0.850193509 |
| H | 4.009403096 | 0.667506203 | 1.734368787 |
| C | 3.577989026 | -0.452130126 | -2.881489202 |
| C | 3.741895070 | 0.616763275 | -0.483766479 |
| H | 4.128718506 | 1.591026820 | -0.775247353 |

| | | | |
|---|---|---|---|
| C | 0.695929351 | 3.332814492 | 1.808627748 |
| C | 1.484365478 | 3.819037376 | 0.579551166 |
| H | 1.863020402 | 2.983948008 | -0.025606399 |
| H | 2.352716794 | 4.413615794 | 0.903541836 |
| H | 0.872694756 | 4.463677380 | -0.070742586 |
| C | -4.591515078 | 3.734216533 | 0.326834517 |
| H | -4.912797261 | 3.455943623 | 1.342567947 |
| H | -5.483224056 | 4.054343337 | -0.236975489 |
| H | -3.913992005 | 4.598067340 | 0.412822461 |
| C | 0.191896187 | 4.581883560 | 2.580429406 |
| H | -0.488181317 | 5.185568352 | 1.959217713 |
| H | 1.043191898 | 5.218649333 | 2.872443133 |
| H | -0.349893884 | 4.297120540 | 3.495754633 |
| C | -3.534683335 | 2.998723664 | -1.819393801 |
| H | -2.894934479 | 3.894576825 | -1.815018495 |
| H | -4.450737515 | 3.253876619 | -2.375403697 |
| H | -3.012639374 | 2.209995600 | -2.381717089 |
| C | 1.627442988 | 2.534763849 | 2.735616640 |
| H | 1.108932905 | 2.198128022 | 3.646387551 |
| H | 2.475465336 | 3.161095040 | 3.053551226 |
| H | 2.041447593 | 1.651858078 | 2.229317089 |
| C | -4.884561225 | 1.371013854 | -0.464428200 |
| H | -4.448927660 | 0.513216508 | -0.994745392 |
| H | -5.798649046 | 1.672218051 | -1.000921822 |
| H | -5.191940482 | 1.033368955 | 0.538094108 |
| C | 4.344301719 | -3.042778929 | -1.357085157 |
| H | 4.683791065 | -4.084548372 | -1.208297078 |
| H | 4.766944337 | -2.703350777 | -2.318242077 |
| H | 3.249677153 | -3.084831857 | -1.496651349 |
| C | 4.635090396 | 2.159543508 | -2.342504917 |
| H | 4.882519495 | 2.496778622 | -3.365895192 |
| H | 5.353688292 | 2.648030148 | -1.662071818 |
| H | 3.643156400 | 2.582699783 | -2.104395010 |
| C | 4.946076921 | 0.338542331 | 2.579062594 |
| H | 5.533078215 | -0.595145189 | 2.614930651 |
| H | 3.921535050 | 0.071695967 | 2.892979524 |
| H | 5.353842812 | 1.002647221 | 3.363543534 |
| C | 4.972220987 | 0.971078003 | 1.178796129 |
| H | 6.016643666 | 1.253198868 | 0.929790795 |
| H | 4.429883518 | 1.935241608 | 1.186772624 |
| C | 4.736531192 | -2.111064082 | -0.200656892 |
| H | 5.837940827 | -2.154948598 | -0.061157306 |
| H | 4.331892126 | -2.499041802 | 0.752578712 |
| C | 4.655930973 | 0.630259597 | -2.192348962 |
| H | 3.960023652 | 0.170158065 | -2.919619402 |
| H | 5.653068310 | 0.249725904 | -2.498600786 |

| | | | |
|---|---|---|---|
| C | 3.433955129 | -2.252765764 | 1.944464791 |
| C | 2.672733333 | -3.565434169 | 1.684423964 |
| H | 1.584912023 | -3.412283913 | 1.646453316 |
| H | 2.881047820 | -4.284360372 | 2.492546210 |
| H | 2.984991091 | -4.037725599 | 0.740254703 |
| C | 5.097356251 | -0.709436658 | -3.059911604 |
| H | 5.697071779 | 0.100568529 | -2.616488390 |
| H | 5.352089171 | -0.775598001 | -4.130654019 |
| H | 5.398368262 | -1.653209528 | -2.578810780 |
| C | 4.952315598 | -2.574125693 | 1.959415715 |
| H | 5.283069424 | -2.969399301 | 0.986201201 |
| H | 5.175877539 | -3.331193553 | 2.729222645 |
| H | 5.551324088 | -1.676878510 | 2.179615217 |
| C | 2.813516728 | -1.595244024 | -3.573051750 |
| H | 3.102059074 | -2.580271817 | -3.174816950 |
| H | 3.039895982 | -1.599850528 | -4.651060743 |
| H | 1.723456543 | -1.490015085 | -3.463335568 |
| C | 3.042697711 | -1.701968428 | 3.327023430 |
| H | 3.573426026 | -0.766914859 | 3.562974622 |
| H | 3.299659669 | -2.431779998 | 4.111284708 |
| H | 1.962087733 | -1.507556861 | 3.393706722 |
| C | 3.223896547 | 0.882380514 | -3.561264755 |
| H | 2.145850205 | 1.087079848 | -3.508906561 |
| H | 3.511805611 | 0.856418502 | -4.624459315 |
| H | 3.754351405 | 1.730085631 | -3.099561034 |
| C | -4.992081099 | 1.801854476 | 1.392128190 |
| H | -5.255150387 | 2.350608769 | 2.314726350 |
| H | -5.588550702 | 2.243185502 | 0.575575304 |
| H | -3.936469597 | 2.042995457 | 1.171614183 |
| C | -4.844351004 | -0.880210893 | -3.133011895 |
| H | -5.132135642 | -0.387015196 | -4.079238771 |
| H | -5.328278629 | -1.871859445 | -3.129560410 |
| H | -3.756818022 | -1.060709686 | -3.186433873 |
| C | -4.606439612 | -3.423413166 | 1.484202248 |
| H | -5.218707093 | -2.996993524 | 2.297289515 |
| H | -3.553222081 | -3.221413002 | 1.745904708 |
| H | -4.740096249 | -4.519688035 | 1.524288885 |
| C | -4.977574223 | -2.827893431 | 0.116503433 |
| H | -6.026511552 | -3.095972318 | -0.130741458 |
| H | -4.379764929 | -3.307168879 | -0.681284566 |
| C | -5.214627538 | 0.286248990 | 1.510259708 |
| H | -6.274135442 | 0.094373121 | 1.781337496 |
| H | -4.641277483 | -0.115623685 | 2.366872698 |
| C | -5.223491024 | -0.055888316 | -1.891881305 |
| H | -4.756986285 | 0.946126825 | -1.945443309 |
| H | -6.313962962 | 0.153971210 | -1.908577610 |

**Table S18:** Cartesian coordinates of the optimized geometry of the Isomers for [Cp''$_2$Zr(μ,η$^{1:1:1:1}$-As$_4$)(Al$^i$Bu$_3$)$_2$] at the B3LYP/def2- SVP level of theory.

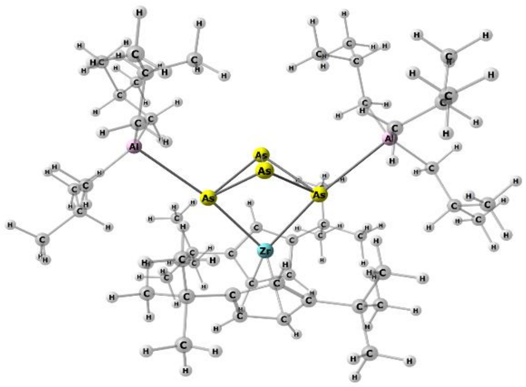

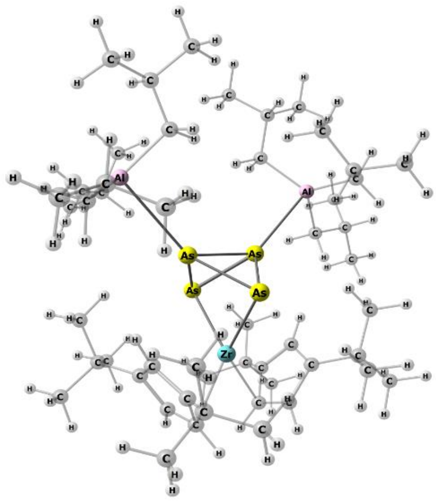

| | | | |
|---|---|---|---|
| As | 0.109464812 | 1.793591151 | 0.012816721 |
| As | -1.225551574 | 0.084032309 | -1.213839675 |
| Al | 0.280202749 | 4.024749469 | -1.914201949 |
| C | -3.304126327 | -2.162877552 | 2.239341521 |
| C | -1.456161204 | 0.580242674 | 4.041242675 |
| H | -0.962022103 | 0.983370468 | 4.922316731 |
| C | -2.467752716 | -0.930783939 | 2.607203362 |
| C | -2.081941680 | 1.339619254 | 3.012524998 |
| C | -1.672775444 | -0.802170769 | 3.785072684 |
| H | -1.375294834 | -1.618665314 | 4.440396396 |
| C | -2.399528316 | 2.835772806 | 3.081500072 |

| | | | |
|---|---|---|---|
| As | -0.570980156 | -0.115670125 | -1.808159263 |
| As | 0.673235595 | -1.012087452 | 0.159012070 |
| C | -2.607934108 | -3.028336640 | 2.644517703 |
| C | -4.413306379 | -1.901630878 | -0.466487082 |
| H | -5.312342351 | -1.586946927 | -0.992420352 |
| C | -2.983994184 | -2.465924218 | 1.266761072 |
| C | -3.271284867 | -2.516645984 | -1.055070202 |
| C | -4.232209706 | -1.854840597 | 0.943385777 |
| H | -4.972805643 | -1.505498161 | 1.660492182 |
| C | -3.199229029 | -3.078357336 | -2.479123491 |
| C | -1.764681091 | -3.463902306 | -2.880687075 |

| | | | |
|---|---|---|---|
| C | 0.000000000 | 6.103775875 | 0.465828277 |
| H | -0.220215704 | 5.280494485 | 1.171919592 |
| C | -2.813414898 | 3.405307081 | 1.714223596 |
| H | -2.005257229 | 3.326007552 | 0.972271346 |
| H | -3.066682569 | 4.472145275 | 1.810452050 |
| H | -3.699615035 | 2.893735596 | 1.308539489 |
| C | -3.813441527 | -2.127480870 | 0.787934054 |
| H | -4.401725197 | -1.220227024 | 0.580527704 |
| H | -4.471876675 | -2.990599481 | 0.603324491 |
| H | -2.992969793 | -2.177509065 | 0.059057280 |
| C | 0.821125348 | 5.526828103 | -0.704232205 |
| H | 1.825803355 | 5.245395131 | -0.331092067 |
| H | 1.035993754 | 6.353468319 | -1.418268583 |
| C | 1.901163614 | 3.445582862 | -2.955208254 |
| H | 2.604063293 | 4.288506503 | -2.794897103 |
| H | 2.390385615 | 2.596588399 | -2.435528589 |
| C | -1.604527206 | 3.826114343 | -2.578279285 |
| H | -1.680765232 | 2.887751385 | -3.163208204 |
| H | -2.255677117 | 3.661093567 | -1.698106349 |
| C | -4.537548339 | -2.122271840 | 3.182211329 |
| H | -4.236527977 | -2.172212884 | 4.240127997 |
| H | -5.201940937 | -2.976929520 | 2.974816737 |
| H | -5.116890622 | -1.197023994 | 3.038593894 |
| C | -2.655740055 | 0.391406600 | 2.113416577 |
| H | -3.235395892 | 0.650032929 | 1.230576509 |
| C | -2.551516648 | -3.481413125 | 2.487694972 |
| H | -1.679241863 | -3.586119988 | 1.827023246 |
| H | -3.216793994 | -4.336991114 | 2.293017192 |
| H | -2.205979362 | -3.568655525 | 3.529207817 |
| C | -1.215424021 | 3.643760689 | 3.641086571 |
| H | -0.923858295 | 3.300465320 | 4.646356177 |
| H | -1.486038620 | 4.707717264 | 3.726386985 |
| H | -0.336491386 | 3.573678894 | 2.985861497 |
| C | -2.212031176 | 4.974889649 | -3.419984738 |
| H | -2.191835872 | 5.895258449 | -2.806830970 |
| C | -1.347775977 | 6.679635765 | 0.012274478 |
| H | -1.200252763 | 7.495581257 | -0.716937169 |
| H | -1.915107858 | 7.097338916 | 0.861296712 |
| H | -1.977592679 | 5.916107292 | -0.468517256 |
| C | -3.600750400 | 2.981366126 | 4.052610588 |
| H | -4.473883998 | 2.415384843 | 3.692298467 |
| H | -3.893783270 | 4.040275128 | 4.140822303 |
| H | -3.350324549 | 2.611547155 | 5.059047998 |
| C | -3.682854732 | 4.702801689 | -3.772965066 |
| H | -3.775543768 | 3.795344068 | -4.395208956 |
| H | -4.131362291 | 5.540353291 | -4.334816317 |
| H | -4.289176039 | 4.544369513 | -2.865994267 |
| C | 3.277871399 | 3.036072841 | -5.061365631 |
| H | 3.846903156 | 2.214785056 | -4.590795984 |
| H | 3.844696313 | 3.967161369 | -4.896472836 |
| H | 3.254255277 | 2.844721257 | -6.148119659 |
| C | -1.402329743 | 5.262630053 | -4.691561011 |
| H | -0.364437461 | 5.557052220 | -4.460794380 |
| H | -1.850885261 | 6.080336287 | -5.279878268 |
| H | -1.354609354 | 4.371973355 | -5.341489063 |
| C | 0.791863476 | 7.164437533 | 1.248421324 |
| H | 1.744831483 | 6.757589236 | 1.624871981 |
| H | 0.223690028 | 7.547972821 | 2.113863610 |
| H | 1.034807439 | 8.025527559 | 0.601448394 |
| C | 1.862726777 | 3.128523165 | -4.467502739 |
| H | 1.351411710 | 3.962292695 | -4.984450924 |
| C | 1.073826900 | 1.850836148 | -4.780165032 |
| H | 1.039784499 | 1.651684720 | -5.864291419 |
| H | 0.035219107 | 1.911425562 | -4.420532781 |
| H | 1.540335640 | 0.972853499 | -4.299648240 |
| Zr | 0.000000000 | 0.000000000 | 2.076595048 |
| As | -0.109464812 | -1.793591151 | 0.012816721 |
| As | 1.225551574 | -0.084032309 | -1.213839675 |
| Al | -0.280202749 | -4.024749469 | -1.914201949 |
| C | 3.304126327 | 2.162877552 | 2.239341521 |
| C | 1.456161204 | -0.580242674 | 4.041242675 |
| H | 0.962022103 | -0.983370468 | 4.922316731 |
| C | 2.467752716 | 0.930783939 | 2.607203362 |
| C | 2.081941680 | -1.339619254 | 3.012524998 |
| C | 1.672775444 | 0.802170769 | 3.785072684 |
| H | 1.375294834 | 1.618665314 | 4.440396396 |
| C | 2.399528316 | -2.835772806 | 3.081500072 |
| C | 0.000000000 | -6.103775875 | 0.465828277 |
| H | 0.220215704 | -5.280494485 | 1.171919592 |
| C | 2.813414898 | -3.405307081 | 1.714223596 |
| H | 2.005257229 | -3.326007552 | 0.972271346 |
| H | 3.066682569 | -4.472145275 | 1.810452050 |
| H | 3.699615035 | -2.893735596 | 1.308539489 |
| C | 3.813441527 | 2.127480870 | 0.787934054 |
| H | 4.401725197 | 1.220227024 | 0.580527704 |
| H | 4.471876675 | 2.990599481 | 0.603324491 |
| H | 2.992969793 | 2.177509065 | 0.059057280 |
| C | -0.821125348 | -5.526828103 | -0.704232205 |
| H | -1.090228643 | -2.594181400 | -2.889840000 |
| H | -1.762469575 | -3.894061020 | -3.894733149 |
| H | -1.334217328 | -4.218037127 | -2.204781601 |
| C | -1.125503630 | -3.430444270 | 2.731960957 |
| H | -0.857406656 | -4.177861350 | 1.969719945 |
| H | -0.917368418 | -3.879746040 | 3.715731017 |
| H | -0.456334160 | -2.567159265 | 2.613764604 |
| C | -3.464388234 | -4.310200145 | 2.825607920 |
| H | -4.540913017 | -4.079597268 | 2.809954799 |
| H | -3.232420624 | -4.791959054 | 3.789800057 |
| H | -3.262996512 | -5.037775073 | 2.024028946 |
| C | -2.385258570 | -2.816263507 | 0.021981927 |
| H | -1.428166055 | -3.319237703 | -0.090774437 |
| C | -2.941266582 | -2.059637865 | 3.792663330 |
| H | -2.334554219 | -1.143467605 | 3.740047130 |
| H | -2.735277131 | -2.539967439 | 4.762316084 |
| H | -4.002681614 | -1.768459184 | 3.790735510 |
| C | -3.770842785 | -2.094615063 | -3.516075913 |
| H | -4.808638107 | -1.808650212 | -3.282536615 |
| H | -3.778020889 | -2.557589438 | -4.515738288 |
| H | -3.168866267 | -1.177073509 | -3.575569840 |
| C | -4.066888140 | -4.364288733 | -2.486146123 |
| H | -3.694541321 | -5.097158153 | -1.753511758 |
| H | -4.043886979 | -4.836201754 | -3.482181840 |
| H | -5.116500534 | -4.142145865 | -2.238182655 |
| Zr | -2.668979688 | -0.140401393 | -0.046229956 |
| As | -0.691809150 | 0.271784881 | 1.815119621 |
| As | 0.448820229 | 1.398746620 | -0.097447870 |
| Al | 2.642184777 | 3.578308713 | -0.433143789 |
| C | -3.058661327 | 2.722762787 | -2.722081117 |
| C | -4.799596013 | 1.171629222 | 0.238364999 |
| H | -5.640947187 | 0.653943364 | 0.694087200 |
| C | -3.402681277 | 2.065080744 | -1.378236652 |
| C | -3.869639749 | 2.011838993 | 0.914065987 |
| C | -4.505456580 | 1.188626364 | -1.152708521 |
| H | -5.090563589 | 0.693938173 | -1.925677655 |
| C | -4.036655349 | 2.554968984 | 2.336937565 |
| C | 2.707089118 | 2.457531514 | -3.359050729 |
| H | 2.056378524 | 1.622837109 | -3.034295202 |
| C | -2.758873504 | 3.241118415 | 2.849169574 |
| H | -1.910915546 | 2.542265577 | 2.906718071 |
| H | -2.926722246 | 3.642419648 | 3.861042201 |
| H | -2.460550132 | 4.085691818 | 2.209499237 |
| C | -1.682314954 | 3.410720130 | -2.710425009 |
| H | -1.605670303 | 4.165020155 | -1.912211835 |
| H | -1.518349781 | 3.932874552 | -3.666248568 |
| H | -0.860978094 | 2.691968296 | -2.582086609 |
| C | 2.461304437 | 3.656679340 | -2.418225091 |
| H | 1.451499941 | 4.068892150 | -2.611068605 |
| H | 3.143078486 | 4.483237311 | -2.719609812 |
| C | 1.651529454 | 5.067153771 | 0.457136453 |
| H | 1.940961797 | 5.952049751 | -0.148860148 |
| H | 0.563862241 | 4.964739087 | 0.263300174 |
| C | 4.112943653 | 2.609730712 | 0.507649206 |
| H | 3.907928305 | 2.624673180 | 1.595985279 |
| H | 4.081548397 | 1.540423838 | 0.226305786 |
| C | -4.139990315 | 3.814776991 | -2.942703145 |
| H | -5.148458818 | 3.376073949 | -2.997071578 |
| H | -3.950887756 | 4.355136819 | -3.884838693 |
| H | -4.134631120 | 4.548846908 | -2.121970341 |
| C | -2.991592582 | 2.517006915 | -0.090772173 |
| H | -2.182364726 | 3.219779966 | 0.092146407 |
| C | -3.121882117 | 1.735929817 | -3.901599700 |
| H | -2.334811500 | 0.970350168 | -3.834025390 |
| H | -2.977381739 | 2.275933493 | -4.850744846 |
| H | -4.094649085 | 1.224303975 | -3.961747362 |
| C | -4.454229090 | 1.455015159 | 3.328912545 |
| H | -5.377379452 | 0.944931631 | 3.011147560 |
| H | -4.648711217 | 1.891701475 | 4.321465503 |
| H | -3.667004890 | 0.697365543 | 3.443671564 |
| C | 5.547049182 | 3.144110297 | 0.267436426 |
| H | 5.765164879 | 3.064077149 | -0.814339973 |
| C | 4.155297126 | 1.955751798 | -3.296379574 |
| H | 4.860396801 | 2.752093474 | -3.593993278 |
| H | 4.315883900 | 1.101120248 | -3.973919190 |
| H | 4.431262104 | 1.629744729 | -2.281932977 |
| C | -5.165777043 | 3.616389673 | 2.270612997 |
| H | -4.912736585 | 4.421244948 | 1.562924391 |
| H | -5.322667234 | 4.071434121 | 3.262472976 |
| H | -6.118273691 | 3.169218080 | 1.946235101 |
| C | 6.600658707 | 2.302127190 | 1.002781642 |
| H | 6.442117415 | 2.342859905 | 2.094582566 |
| H | 7.624507726 | 2.660577105 | 0.799171805 |
| H | 6.548631905 | 1.243974531 | 0.700256629 |
| C | 1.297696610 | 6.760010169 | 2.335846264 |
| H | 0.204213169 | 6.781578217 | 2.181872248 |
| H | 1.732594656 | 7.566414436 | 1.722833337 |
| H | 1.489707808 | 7.002076216 | 3.395422124 |

| | | | |
|---|---|---|---|
| H | -1.825803355 | -5.245395131 | -0.331092067 |
| H | -1.035993754 | -6.353468319 | -1.418268583 |
| C | -1.901163614 | -3.445582862 | -2.955208254 |
| H | -2.604063293 | -4.288506503 | -2.794897103 |
| H | -2.390385615 | -2.596588399 | -2.435528589 |
| C | 1.604527206 | -3.826114343 | -2.578279285 |
| H | 1.680765232 | -2.887751385 | -3.163208204 |
| H | 2.255677117 | -3.661093567 | -1.698106349 |
| C | 4.537548339 | 2.122271840 | 3.182211329 |
| H | 4.236527977 | 2.172212884 | 4.240127997 |
| H | 5.201940937 | 2.976929520 | 2.974816737 |
| H | 5.116890622 | 1.197023994 | 3.038593894 |
| C | 2.655740055 | -0.391406600 | 2.113416577 |
| H | 3.235395892 | -0.650032929 | 1.230576509 |
| C | 2.551516648 | 3.481413125 | 2.487694972 |
| H | 1.679241863 | 3.586119988 | 1.827023246 |
| H | 3.216793994 | 4.336991114 | 2.293017192 |
| H | 2.205979362 | 3.568655525 | 3.529207817 |
| C | 1.215424021 | -3.643760689 | 3.641086571 |
| H | 0.923858295 | -3.300465320 | 4.646356177 |
| H | 1.486038620 | -4.707717264 | 3.726386985 |
| H | 0.336491386 | -3.573678894 | 2.985861497 |
| C | 2.212031176 | -4.974889649 | -3.419984738 |
| H | 2.191835872 | -5.895258449 | -2.806830970 |
| C | 1.347775977 | -6.679635765 | 0.012274478 |
| H | 1.200252763 | -7.495581257 | -0.716937169 |
| H | 1.915107858 | -7.097338916 | 0.861296712 |
| H | 1.977592679 | -5.916107292 | -0.468517256 |
| C | 3.600750400 | -2.981366126 | 4.052610588 |
| H | 4.473883998 | -2.415384843 | 3.692298467 |
| H | 3.893783270 | -4.040275128 | 4.140822303 |
| H | 3.350324549 | -2.611547155 | 5.059047998 |
| C | 3.682854732 | -4.702801689 | -3.772965066 |
| H | 3.775543768 | -3.795344068 | -4.395208956 |
| H | 4.131362291 | -5.540353291 | -4.334816317 |
| H | 4.289176039 | -4.544369513 | -2.865994267 |
| C | -3.277871399 | -3.036072841 | -5.061365631 |
| H | -3.846903156 | -2.214785056 | -4.590795984 |
| H | -3.844696313 | -3.967161369 | -4.896472836 |
| H | -3.254255277 | -2.844721257 | -6.148119659 |
| C | 1.402329743 | -5.262630053 | -4.691561011 |
| H | 0.364437461 | -5.557052220 | -4.460794380 |
| H | 1.850885261 | -6.080336287 | -5.279878268 |
| H | 1.354609354 | -4.371973355 | -5.341489063 |
| C | -0.791863476 | -7.164437533 | 1.248421324 |
| H | -1.744831483 | -6.757589236 | 1.624871981 |
| H | -0.223690028 | -7.547972821 | 2.113863610 |
| H | -1.034807439 | -8.025527559 | 0.601448394 |
| C | -1.862726777 | -3.128523165 | -4.467502739 |
| H | -1.351411710 | -3.962292695 | -4.984450924 |
| C | -1.073826900 | -1.850836148 | -4.780165032 |
| H | -1.039784499 | -1.651684720 | -5.864291419 |
| H | -0.035219107 | -1.911425562 | -4.420532781 |
| H | -1.540335640 | -0.972853499 | -4.299648240 |
| C | 5.684650984 | 4.625327679 | 0.648578835 |
| H | 4.995447264 | 5.267593344 | 0.072029095 |
| H | 6.705776539 | 4.998468851 | 0.465164256 |
| H | 5.460504889 | 4.781098379 | 1.718030561 |
| C | 2.320470109 | 2.788075775 | -4.809373626 |
| H | 1.266355373 | 3.102923062 | -4.881429448 |
| H | 2.458981243 | 1.920493745 | -5.477143810 |
| H | 2.939789924 | 3.614152354 | -5.201376437 |
| C | 1.875997791 | 5.389257999 | 1.949337450 |
| H | 2.966874999 | 5.436146966 | 2.129748685 |
| C | 1.310583008 | 4.295818965 | 2.864502903 |
| H | 1.504060365 | 4.516156819 | 3.927569047 |
| H | 1.747525731 | 3.308331963 | 2.645327804 |
| H | 0.217411941 | 4.207886735 | 2.739461208 |
| H | 2.254464259 | -1.557793213 | 2.986787245 |
| C | 3.219872525 | -2.075717921 | 3.147578991 |
| C | 3.268911158 | -3.304337310 | 2.210260332 |
| Al | 3.065383587 | -3.180560153 | 0.229286886 |
| H | 2.500234229 | -4.030742418 | 2.538745664 |
| H | 4.233192361 | -3.830260574 | 2.382288309 |
| C | 1.757622333 | -4.475222996 | -0.557883220 |
| H | 0.806545832 | -4.338227425 | -0.006317716 |
| H | 1.532149384 | -4.199117157 | -1.606912546 |
| C | 4.300448541 | -2.181659248 | -0.976013989 |
| H | 4.152091297 | -2.586638036 | -1.997499628 |
| H | 3.938916872 | -1.136507474 | -1.039872215 |
| C | 5.813939040 | -2.160330040 | -0.659444987 |
| H | 5.948735514 | -1.702889154 | 0.338282235 |
| C | 4.332652564 | -1.064015430 | 2.847321560 |
| H | 5.327902014 | -1.525688670 | 2.971986126 |
| H | 4.282814990 | -0.196893375 | 3.526172953 |
| H | 4.272567091 | -0.678301409 | 1.817553095 |
| C | 6.592806828 | -1.296239098 | -1.663090659 |
| H | 6.507003436 | -1.707513269 | -2.684193508 |
| H | 7.666242560 | -1.245676137 | -1.412041564 |
| H | 6.204569377 | -0.265583870 | -1.689545262 |
| C | 0.953814860 | -6.870670551 | -0.938890527 |
| H | 0.669723012 | -6.662028027 | -1.985317857 |
| H | 0.064352989 | -6.694644767 | -0.311647459 |
| H | 1.206076904 | -7.942830120 | -0.869033913 |
| C | 6.411986485 | -3.572689554 | -0.596767710 |
| H | 5.931111530 | -4.189966221 | 0.182142482 |
| H | 7.490457445 | -3.547342250 | -0.368691708 |
| H | 6.289575564 | -4.097183008 | -1.560535992 |
| C | 3.261647486 | -2.493972739 | 4.625673136 |
| H | 2.441041674 | -3.188673652 | 4.869566385 |
| H | 3.177463149 | -1.623771186 | 5.298965173 |
| H | 4.210389283 | -3.007956071 | 4.860562000 |
| C | 2.127631373 | -5.978093474 | -0.506697203 |
| H | 2.363011631 | -6.240672918 | 0.543472163 |
| C | 3.372986930 | -6.291871558 | -1.347806066 |
| H | 3.635165707 | -7.361802496 | -1.300275916 |
| H | 4.255021844 | -5.722071065 | -1.012066449 |
| H | 3.204289826 | -6.036922744 | -2.408578978 |

**Table S19:** Cartesian coordinates of the optimized geometry of the Isomers for $[Cp''_2Zr(\mu,\eta^{1:1:1}\text{-}As_4)(Al^iBu_3)]$ at the B3LYP/def2- SVP level of theory.

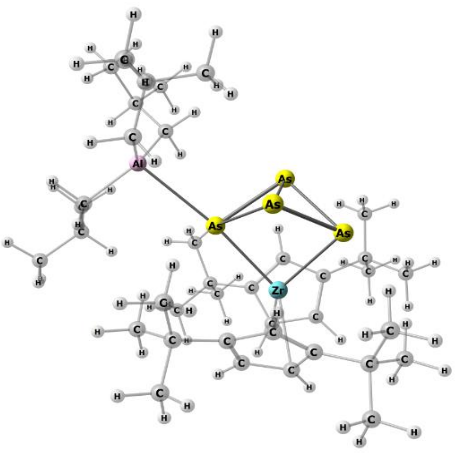


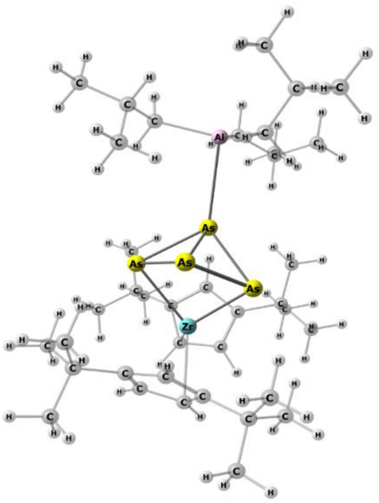

| | | | |
|---|---|---|---|
| As | -1.860524802 | -0.616148292 | -2.145534620 |
| As | 0.160610311 | -1.936888428 | -1.526003022 |
| C | -0.421565877 | -2.422423461 | 3.045922279 |
| C | -3.565271140 | -0.679940652 | 1.908277090 |
| H | -4.419564726 | -0.028870009 | 2.081140554 |
| C | -1.549417105 | -1.768325392 | 2.238653342 |
| C | -3.485879037 | -1.711179070 | 0.928284117 |
| C | -2.379828127 | -0.700901034 | 2.694137309 |
| H | -2.188964636 | -0.073153370 | 3.562788436 |
| C | -4.667958780 | -2.272882421 | 0.129669195 |
| C | -4.211570814 | -3.246824718 | -0.971275196 |
| H | -3.565328318 | -2.755907381 | -1.714527069 |
| H | -5.088191478 | -3.647157282 | -1.504784809 |
| H | -3.661965741 | -4.105408142 | -0.555605610 |
| C | 0.429877809 | -3.381106837 | 2.194728397 |
| H | -0.176557676 | -4.189588193 | 1.757690173 |
| H | 1.199698096 | -3.855056666 | 2.823492820 |
| H | 0.944913152 | -2.859351849 | 1.376274055 |
| C | -1.122025528 | -3.255531811 | 4.152814110 |
| H | -1.718510488 | -2.615002154 | 4.820961518 |
| H | -0.372303929 | -3.782620149 | 4.765554490 |
| H | -1.795881821 | -4.008981681 | 3.715860602 |
| C | -2.220347598 | -2.340539870 | 1.122012209 |
| H | -1.855101338 | -3.189136524 | 0.549043336 |
| C | 0.494244683 | -1.390345831 | 3.724991550 |
| H | 1.054021727 | -0.796552150 | 2.988441729 |
| H | 1.229717591 | -1.901463647 | 4.365748167 |
| H | -0.072080001 | -0.698020865 | 4.366433303 |
| C | -5.527844546 | -1.161247886 | -0.498100016 |
| H | -5.887920824 | -0.445953718 | 0.258238235 |
| H | -6.415815189 | -1.597004157 | -0.983576265 |
| H | -4.970237467 | -0.600192926 | -1.260623939 |
| C | -5.542674104 | -3.060695670 | 1.140184166 |
| H | -4.962528227 | -3.860211419 | 1.626834657 |
| H | -6.399117371 | -3.525852044 | 0.624702559 |
| H | -5.937573525 | -2.402457096 | 1.929625899 |
| Zr | -1.732935156 | 0.218492275 | 0.436173906 |
| As | 0.950175248 | -0.186958944 | 0.092709147 |
| As | 0.431088269 | 0.373719875 | -2.289892162 |
| Al | 3.842047040 | -0.399431938 | 0.112251451 |
| C | -3.280753060 | 2.963409676 | -1.970679121 |
| C | -2.516232882 | 2.285266672 | 1.654701348 |
| H | -2.821609965 | 2.160823386 | 2.691341348 |
| C | -2.646516098 | 2.513080621 | -0.646445721 |
| C | -1.231548507 | 2.701704327 | 1.205971857 |
| C | -3.371628870 | 2.149140502 | 0.527026444 |
| H | -4.433042388 | 1.912170649 | 0.568925647 |
| C | -0.130560587 | 3.296162199 | 2.087888103 |
| C | 3.907393519 | 0.811076103 | 3.039786384 |
| H | 2.826900070 | 1.038192797 | 2.962790153 |
| C | 1.213468542 | 3.391506440 | 1.346181910 |
| H | 1.582495699 | 2.403969796 | 1.032558938 |
| H | 1.977408859 | 3.836495960 | 2.001926598 |
| H | 1.141065960 | 4.026705981 | 0.450064076 |
| C | -2.250359246 | 3.090951982 | -3.106968992 |
| H | -1.444290562 | 3.796153068 | -2.851776725 |
| H | -2.742913894 | 3.474641921 | -4.014474795 |
| H | -1.793226293 | 2.124188239 | -3.359249927 |
| C | 4.222979415 | -0.353128057 | 2.080362356 |
| H | 3.766767454 | -1.280222379 | 2.481038076 |
| H | 5.315228907 | -0.560215797 | 2.132563887 |
| C | 4.096073466 | -2.288406137 | -0.530480348 |
| H | 4.728556571 | -2.730910328 | 0.266436444 |
| H | 3.139707606 | -2.843782576 | -0.444091450 |
| C | 4.161951467 | 1.194232086 | -1.071726214 |
| H | 3.857589467 | 0.923263135 | -2.102801445 |
| H | 3.451521273 | 1.988779967 | -0.772085609 |
| C | -3.865083025 | 4.375188943 | -1.694378047 |
| H | -4.644052314 | 4.342820827 | -0.916853507 |
| H | -4.315802391 | 4.787998469 | -2.612031633 |
| H | -3.080738300 | 5.070398914 | -1.356628620 |
| C | -1.317726774 | 2.790153134 | -0.215705404 |
| H | -0.506878389 | 3.109041134 | -0.866178605 |
| C | -4.431176097 | 2.048468081 | -2.426760180 |
| H | -4.069305987 | 1.047278913 | -2.703174868 |
| H | -4.923959117 | 2.479471896 | -3.312780915 |
| H | -5.200711240 | 1.933572450 | -1.648294540 |
| C | 0.052720030 | 2.498293667 | 3.391295706 |
| H | -0.881911843 | 2.442466298 | 3.971825463 |
| H | 0.805499675 | 2.983411121 | 4.032264453 |
| H | 0.393855499 | 1.473101400 | 3.192974149 |
| C | 5.579404932 | 1.813485373 | -1.131070289 |
| H | 5.844711015 | 2.160917829 | -0.115064759 |
| C | 4.664890155 | 2.094153255 | 2.674358442 |
| H | 5.755983019 | 1.929131335 | 2.714649373 |

| | | | |
|---|---|---|---|
| As | -0.115236709 | 0.415268498 | -1.855085918 |
| As | 0.443525807 | -1.864121641 | -1.003115775 |
| C | -3.492005492 | -3.304631855 | 0.864439755 |
| C | -4.393221181 | -0.005995000 | -0.724437246 |
| H | -5.002505081 | 0.892694158 | -0.788541640 |
| C | -3.495156975 | -1.978751916 | 0.090107588 |
| C | -3.673466302 | -0.607690722 | -1.797300360 |
| C | -4.272101923 | -0.830915773 | 0.427574508 |
| H | -4.780931624 | -0.664768494 | 1.375313773 |
| C | -3.838286558 | -0.267518866 | -3.282207673 |
| C | -2.730899380 | -0.892188656 | -4.149386433 |
| H | -1.733253032 | -0.512116309 | -3.881667043 |
| H | -2.904363634 | -0.651951573 | -5.210380674 |
| H | -2.711211822 | -1.989440762 | -4.062573705 |
| C | -2.422288553 | -4.283080970 | 0.346611535 |
| H | -2.563566019 | -4.516388393 | -0.720128727 |
| H | -2.488061115 | -5.233846441 | 0.898971417 |
| H | -1.404153378 | -3.890615245 | 0.479744182 |
| C | -4.885685806 | -3.942279995 | 0.618584264 |
| H | -5.693395955 | -3.307272349 | 1.014528673 |
| H | -4.949505450 | -4.924587335 | 1.115041907 |
| H | -5.070027490 | -4.090707953 | -0.456920502 |
| C | -3.094655672 | -1.796902699 | -1.264091437 |
| H | -2.500884297 | -2.506684413 | -1.834829923 |
| C | -3.311361477 | -3.109710644 | 2.380184496 |
| H | -2.305341481 | -2.737147000 | 2.622919508 |
| H | -3.443908519 | -4.070507310 | 2.902742801 |
| H | -4.048127589 | -2.405416212 | 2.795576587 |
| C | -3.877643417 | 1.251467114 | -3.524711270 |
| H | -4.676606716 | 1.738762731 | -2.943714350 |
| H | -4.073941877 | 1.461689254 | -4.588221833 |
| H | -2.923914209 | 1.726400097 | -3.257678429 |
| C | -5.197951597 | -0.872167256 | -3.720168438 |
| H | -5.217687448 | -1.961505982 | -3.560237516 |
| H | -5.375659308 | -0.680880587 | -4.791447350 |
| H | -6.032623443 | -0.432980680 | -3.151995319 |
| Zr | -2.026437829 | 0.281332716 | 0.094626351 |
| As | -0.200440160 | -1.307787031 | 1.355075808 |
| As | 1.431104894 | 0.082412404 | 0.085899821 |
| Al | 4.425244914 | -0.164168646 | 0.318863041 |
| C | -1.022390464 | 4.015843983 | -0.640436861 |
| C | -3.220404184 | 1.918867271 | 1.592097372 |
| H | -4.195164584 | 1.620837433 | 1.972462152 |
| C | -1.610769899 | 2.928812232 | 0.268642386 |
| C | -1.978964708 | 1.791106282 | 2.279252540 |
| C | -2.995103181 | 2.598687455 | 0.362886254 |
| H | -3.769539686 | 2.906952904 | -0.336783173 |
| C | -1.817074084 | 1.467430610 | 3.768443316 |
| C | 4.305904734 | 2.971635160 | 0.429368081 |
| H | 3.222019593 | 2.917151351 | 0.210879161 |
| C | -0.354994854 | 1.167537179 | 4.143010075 |
| H | 0.032275714 | 0.282151498 | 3.616778862 |
| H | -0.276661407 | 0.972953773 | 5.224381492 |
| H | 0.308628469 | 2.015899957 | 3.915629591 |
| C | 0.515025705 | 3.981842863 | -0.675488683 |
| H | 0.950672458 | 4.104999554 | 0.327573597 |
| H | 0.893713660 | 4.808508569 | -1.297236697 |
| H | 0.897141168 | 3.043451127 | -1.101762781 |
| C | 4.723563789 | 1.649252245 | 1.104158403 |
| H | 4.270878264 | 1.602429833 | 2.114571670 |
| H | 5.816829777 | 1.689933694 | 1.307528501 |
| C | 4.493520347 | -1.559169693 | 1.747550661 |
| H | 5.316087867 | -1.193094805 | 2.398021955 |
| H | 3.593888612 | -1.470565699 | 2.389958792 |
| C | 4.742076600 | -0.581572157 | -1.610987941 |
| H | 4.427515746 | -1.625847180 | -1.804713133 |
| H | 4.061184334 | 0.039097422 | -2.224682073 |
| C | -1.460522921 | 5.369771735 | -0.020666929 |
| H | -2.557156471 | 5.468293267 | -0.004460355 |
| H | -1.050843674 | 6.208970544 | -0.606739393 |
| H | -1.097711506 | 5.468117104 | 1.014410333 |
| C | -0.995225652 | 2.375722701 | 1.427708200 |
| H | 0.061468349 | 2.459055677 | 1.669679403 |
| C | -1.563840947 | 3.945194306 | -2.078819095 |
| H | -1.237276718 | 3.026409815 | -2.587593736 |
| H | -1.191605134 | 4.801137781 | -2.663979530 |
| H | -2.663564332 | 3.982269549 | -2.108130869 |
| C | -2.707416922 | 0.290252504 | 4.205115100 |
| H | -3.768281371 | 0.469427221 | 3.969117166 |
| H | -2.636245969 | 0.143471310 | 5.294672011 |
| H | -2.403547936 | -0.647066970 | 3.719554715 |
| As | -0.115236709 | 0.415268498 | -1.855085918 |
| As | 0.443525807 | -1.864121641 | -1.003115775 |
| C | -3.492005492 | -3.304631855 | 0.864439755 |
| C | -4.393221181 | -0.005995000 | -0.724437246 |

| | | | |
|---|---|---|---|
| H | 4.431006829 | 2.915382443 | 3.373106137 |
| H | 4.420109365 | 2.437868706 | 1.658230552 |
| C | -0.588040391 | 4.733627438 | 2.449984775 |
| H | -0.736761397 | 5.342561546 | 1.544574784 |
| H | 0.172316713 | 5.230961483 | 3.074418474 |
| H | -1.536377008 | 4.724165394 | 3.009517758 |
| C | 5.622490702 | 3.044498464 | -2.050477859 |
| H | 5.377220645 | 2.765432243 | -3.090353869 |
| H | 6.620027917 | 3.517246310 | -2.057766736 |
| H | 4.892262410 | 3.806799813 | -1.732584269 |
| C | 5.132517989 | -4.106151266 | -1.989342499 |
| H | 4.244932302 | -4.756266366 | -1.892662022 |
| H | 5.833290893 | -4.383714221 | -1.184786373 |
| H | 5.616115968 | -4.342702603 | -2.953152972 |
| C | 6.649543836 | 0.796965036 | -1.551999416 |
| H | 6.703133775 | -0.056642932 | -0.854990279 |
| H | 7.652138875 | 1.255344338 | -1.585029464 |
| H | 6.437266182 | 0.390067777 | -2.555783748 |
| C | 4.182786778 | 0.435107156 | 4.505184102 |
| H | 3.608979318 | -0.456887743 | 4.805688393 |
| H | 3.919927833 | 1.253963741 | 5.197608939 |
| H | 5.252042735 | 0.203240549 | 4.653941547 |
| C | 4.734194478 | -2.623991647 | -1.897321025 |
| H | 5.664540290 | -2.034306515 | -2.000055205 |
| C | 3.827267034 | -2.246973802 | -3.075538901 |
| H | 4.309278980 | -2.464657754 | -4.043612056 |
| H | 3.566036013 | -1.177250609 | -3.065707020 |
| H | 2.882452055 | -2.817756851 | -3.041505644 |

| | | | |
|---|---|---|---|
| H | -5.002505081 | 0.892694158 | -0.788541640 |
| C | -3.495156975 | -1.978751916 | 0.090107588 |
| C | -3.673466302 | -0.607690722 | -1.797300360 |
| C | -4.272101923 | -0.830915773 | 0.427574508 |
| H | -4.780931624 | -0.664768494 | 1.375313773 |
| C | -3.838286558 | -0.267518866 | -3.282207673 |
| C | -2.730899380 | -0.892188656 | -4.149386433 |
| H | -1.733253032 | -0.512116309 | -3.881667043 |
| H | -2.904363634 | -0.651951573 | -5.210380674 |
| H | -2.711211822 | -1.989440762 | -4.062573705 |
| C | -2.422288553 | -4.283080970 | 0.346611535 |
| H | -2.563566019 | -4.516388393 | -0.720128727 |
| H | -2.488061115 | -5.233846441 | 0.898971417 |
| H | -1.404153378 | -3.890615245 | 0.479744182 |
| C | -4.885685806 | -3.942279995 | 0.618584264 |
| H | -5.693395955 | -3.307272349 | 1.014528673 |
| H | -4.949505450 | -4.924587335 | 1.115041907 |
| H | -5.070027490 | -4.090707953 | -0.456920502 |
| C | -3.094655672 | -1.796902699 | -1.264091437 |
| H | -2.500884297 | -2.506684413 | -1.834829923 |
| C | -3.311361477 | -3.109710644 | 2.380184496 |
| H | -2.305341481 | -2.737147000 | 2.622919508 |
| H | -3.443908519 | -4.070507310 | 2.902742801 |
| H | -4.048127589 | -2.405416212 | 2.795576587 |
| C | -3.877643417 | 1.251467114 | -3.524711270 |
| H | -4.676606716 | 1.738762731 | -2.943714350 |
| H | -4.073941877 | 1.461689254 | -4.588221833 |
| H | -2.923914209 | 1.726400097 | -3.257678429 |

**Table S20:** Cartesian coordinates of the optimized geometry for [Cp''$_2$Zr(μ,η$^{1:1:1}$-As$_4$)(B(C$_6$F$_5$)$_3$)] at the B3LYP/def2- SVP level of theory.

| | | | |
|---|---|---|---|
| Zr | 2.490684707 | -0.231908978 | 0.090899804 |
| As | 2.379599655 | 2.225005194 | -1.154775720 |
| As | 0.265911360 | 2.669075850 | 0.105223132 |
| As | -0.263009465 | 0.235677654 | -0.089055036 |
| As | 0.174483336 | 1.607780323 | -2.112172926 |
| F | -0.896871250 | -1.748379114 | -2.111394067 |
| F | -5.259108590 | -0.448143953 | -0.756037859 |
| F | -3.469502870 | 1.515032469 | -2.417197063 |
| F | -2.538968988 | 1.776920681 | 2.235930619 |
| F | -1.947105529 | -3.237601460 | -4.028158101 |
| F | -4.303637215 | 4.030327193 | -2.375474919 |
| F | -1.150339146 | -2.517547083 | 0.768904687 |
| F | -4.785124539 | 0.308759421 | 1.960560135 |
| F | -4.659989947 | -3.384738676 | -4.366390725 |
| F | -6.297477979 | -1.966171436 | -2.698500873 |
| F | -3.379600465 | 4.302841269 | 2.245706994 |
| F | -4.278937581 | 5.473443326 | -0.056241641 |
| F | -5.353407828 | -1.303618009 | 3.983704916 |
| F | -1.737045376 | -4.108413324 | 2.818990029 |
| C | -3.025085703 | -0.987574940 | -1.337953494 |
| F | -3.855899016 | -3.535486732 | 4.460463438 |
| C | -4.407614247 | -1.115611175 | -1.547919337 |
| C | -2.243187454 | -1.745501554 | -2.209816987 |
| C | 3.183734026 | -2.677775789 | -0.555168086 |
| C | -2.763510362 | -2.547822292 | -3.230420001 |
| C | -3.006937701 | 2.274123109 | 1.079298773 |
| C | -4.976296255 | -1.899642880 | -2.551443627 |
| C | -4.143066205 | -2.627178242 | -3.404019720 |
| C | -3.428516425 | 3.602788236 | 1.113563507 |
| C | -3.034659694 | 1.467054338 | -0.069965732 |
| C | -2.892618752 | -0.976223988 | 1.278263624 |
| C | -3.462696274 | 2.135086962 | -1.228065865 |
| C | -3.881259661 | 4.205491726 | -0.060959107 |
| C | 4.364633254 | -0.225249051 | 1.739081576 |
| H | 5.323044008 | -0.614900698 | 1.402412729 |
| C | 4.003400380 | 1.152333329 | 1.823669802 |
| C | 2.696555850 | 1.184562214 | 2.391222282 |
| H | 2.131854350 | 2.090367293 | 2.598402059 |
| C | -2.193411116 | -2.158730289 | 1.550563239 |
| C | -3.981404074 | -0.743513466 | 2.134443001 |
| C | -3.896859298 | 3.464107034 | -1.240992067 |
| C | 4.978398576 | 2.333513629 | 1.745082515 |
| C | 3.589978945 | -1.033298331 | -2.173065876 |
| C | 5.938068518 | 2.228892901 | 0.548260947 |
| H | 6.465371738 | 1.262320863 | 0.525353406 |
| H | 6.703263371 | 3.019008885 | 0.607269042 |
| H | 5.406044613 | 2.351733985 | -0.404501135 |
| C | 4.163161097 | -1.795193113 | -1.102266579 |
| H | 5.223796349 | -1.816038913 | -0.858622303 |
| C | 3.317474575 | -1.004219470 | 2.312289838 |
| H | 3.350597287 | -2.074743028 | 2.481124522 |
| C | 4.390598865 | -0.371789981 | -3.302514104 |
| C | 2.286854854 | -0.127423198 | 2.758398536 |

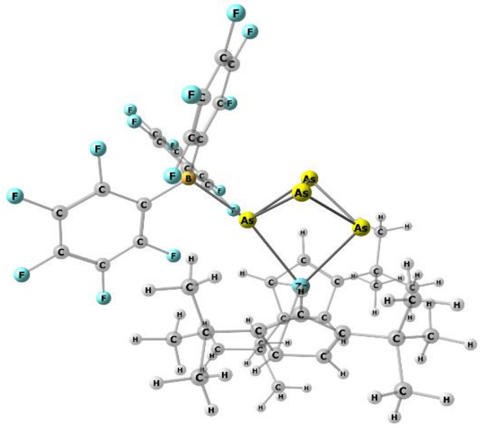

| | | | |
|---|---|---|---|
| C | 4.251411204 | 3.689631175 | 1.700006556 |
| H | 3.616265205 | 3.788270206 | 0.807208621 |
| H | 4.989199613 | 4.507117525 | 1.676356736 |
| H | 3.620443732 | 3.846262275 | 2.588475578 |
| C | 2.211115053 | -1.367867646 | -2.192912277 |
| H | 1.466964553 | -0.991046988 | -2.888544235 |
| C | 3.463102857 | -3.993639890 | 0.183034823 |
| C | 5.814384131 | 2.275143135 | 3.051751293 |
| H | 5.166888406 | 2.328907290 | 3.940729505 |
| H | 6.518245277 | 3.122593977 | 3.090244022 |
| H | 6.398459233 | 1.343960159 | 3.115197471 |
| C | 2.319409585 | -4.397452805 | 1.131300222 |
| H | 2.217404246 | -3.710733023 | 1.980889183 |
| H | 2.503037032 | -5.404876189 | 1.537030124 |
| H | 1.347868408 | -4.424127530 | 0.615291946 |
| C | -2.483768254 | -3.023803289 | 2.604273879 |
| C | -3.557261931 | -2.731322266 | 3.444888867 |
| C | -4.313643782 | -1.584966216 | 3.201911132 |
| C | 5.070768893 | -1.530214284 | -4.079086077 |
| H | 5.784626748 | -2.078202747 | -3.444822582 |
| H | 5.623340561 | -1.133038723 | -4.946220990 |
| H | 4.325003900 | -2.250371681 | -4.450314998 |
| C | 1.955434747 | -2.350894250 | -1.197467762 |
| H | 0.992919503 | -2.831126868 | -1.036307455 |
| C | 4.805935630 | -3.980626840 | 0.938092290 |
| H | 5.652541620 | -3.805511981 | 0.256240160 |
| H | 4.973495198 | -4.958372778 | 1.416293455 |
| H | 4.846979120 | -3.217244361 | 1.727416027 |
| C | 3.480827469 | 0.390071388 | -4.282765014 |
| H | 2.730362984 | -0.271872195 | -4.741761570 |
| H | 4.086144042 | 0.814726023 | -5.099080353 |
| H | 2.952187261 | 1.220890253 | -3.793414306 |
| B | -2.553218477 | -0.082728698 | -0.051304302 |
| C | 1.202609703 | -0.475413481 | 3.781352579 |
| C | 3.563618647 | -5.072406930 | -0.930449378 |
| H | 2.616910489 | -5.163035593 | -1.484160279 |
| H | 3.797009155 | -6.055097442 | -0.488767985 |
| H | 4.356541266 | -4.825501552 | -1.653430296 |
| C | 1.848922686 | -0.253813587 | 5.174874833 |
| H | 2.139755814 | 0.798518187 | 5.317134474 |
| H | 2.749115465 | -0.875345653 | 5.301937350 |
| H | 1.134390268 | -0.520218858 | 5.970745054 |
| C | 5.482639686 | 0.576967310 | -2.780658319 |
| H | 5.043528770 | 1.469128936 | -2.312523020 |
| H | 6.119614521 | 0.916633105 | -3.612844720 |
| H | 6.136837388 | 0.087725087 | -2.042212264 |
| C | -0.032072063 | 0.430958786 | 3.668034658 |
| H | -0.732769162 | 0.218838297 | 4.490581111 |
| H | -0.573327766 | 0.269108678 | 2.727777207 |
| H | 0.225519173 | 1.499204137 | 3.728891211 |
| C | 0.771016342 | -1.947119203 | 3.673653832 |
| H | 1.602302952 | -2.632859896 | 3.897759582 |
| H | 0.388997814 | -2.187536148 | 2.672241510 |
| H | -0.026542699 | -2.163939341 | 4.400604649 |

## 4. References


[1] Rigaku Oxford Diffraction, *CrysAlisPro Software System Version 1.171.38.43*, **2015**.

[2] L. J. Bourhis, O. V. Dolomanov, R. J. Gildea, J. A. K. Howard, H. Puschmann, OLEX2: a complete structure solution, refinement and analysis program *J. Appl. Crystallogr.* **2009**, *42*, 339–341.

[3] G. M. Sheldrick, SHELXT - integrated space-group and crystal-structure determination *Acta Crystallogr., Sect, A* **2015**, *71*, 3-8.

[4] G. M. Sheldrick, Crystal structure refinement with SHELXL *Acta Crystallogr., Sect, C* **2015**, *71*, 3-8.

[5] M. J. Frisch, G.W. Trucks, H.B. Schlegel, G.E. Scuseria, M.A. Robb, J.R. Cheeseman, G. Scalmani, V. Barone, B. Mennucci, G.A. Petersson, H. et al. *Gaussian Inc. Wallingford CT,* **2013**.

[6] A. D. Becke, Density-functional thermochemistry. III. The role of exact exchange *J. Chem. Phys.* **1993**, *98*, 5648.

[7] a) D. Andrae, U. Häußermann, M. Dolg, H. Stoll, H. Preuß, Energy-adjustedab initio pseudopotentials for the second and third row transition elements, *Ther. Chim. Acta* **1990**, *77*, 123;
b) F. Weigend, R. Alrichs, Balanced basis sets of split valence, triple zeta valence and quadruple zeta valence quality for H to Rn: Design and assessment of accuracy, *Phys. Chem. Chem. Phys.* **2005**, *7*, 3297-3305.

[8] a) A. Schäfer, H. Horn, R. Ahlrichs, Fully optimized contracted Gaussian basis sets for atoms Li to Kr, *J. Chem. Phys.* **1992**, *97*, 2571–2577;
b) F. Weigend, Accurate Coulomb-fitting basis sets for H to Rn, *Phys. Chem. Chem. Phys.* **2006**, *8*, 1057–1065;
c) F. Weigend, R. Ahlrichs, Balanced basis sets of split valence, triple zeta valence and quadruple zeta valence quality for H to Rn: Design and assessment of accuracy, *Phys. Chem. Chem. Phys.* **2005**, *7*, 3297–3305;
d) K. Eichkorn, F. Weigend, O. Treutler, R. Ahlrichs, Auxiliary basis sets for main row atoms and transition metals and their use to approximate Coulomb potentials, *Theor. Chem. Acc.* **1997**, *97*, 119.

[9] Chemcraft - graphical software for visualization of quantum chemistry computations. http://www.chemcraftprog.com.

[10] S. Grimme, S. Ehrlich, L. Goerigk, Effect of the damping function in dispersion corrected density functional theory, *J. Comput. Chem.* **2011**, *32*, 1456.
